# Microbial Succession in the Gut: Directional Trends of Taxonomic and Functional Change in a Birth Cohort of Spanish Infants

**DOI:** 10.1371/journal.pgen.1004406

**Published:** 2014-06-05

**Authors:** Yvonne Vallès, Alejandro Artacho, Alberto Pascual-García, Maria Loreto Ferrús, María José Gosalbes, Juan José Abellán, M. Pilar Francino

**Affiliations:** 1 Unidad Mixta de Investigación en Genómica y Salud, Fundación para el Fomento de la Investigación Sanitaria y Biomédica de la Comunitat Valenciana (FISABIO)-Salud Pública/Institut Cavanilles de Biodiversitat i Biologia Evolutiva (Universitat de València), València, Spain; 2 Centro de Biología Molecular “Severo Ochoa” (CSIC-Universidad Autónoma de Madrid), Madrid, Spain; 3 CIBER en Epidemiología y Salud Pública (CIBERESP), Spain; 4 School of Natural Sciences, University of California Merced, Merced, California, United States of America; University of Toronto, Canada

## Abstract

In spite of its major impact on life-long health, the process of microbial succession in the gut of infants remains poorly understood. Here, we analyze the patterns of taxonomic and functional change in the gut microbiota during the first year of life for a birth cohort of 13 infants. We detect that individual instances of gut colonization vary in the temporal dynamics of microbiota richness, diversity, and composition at both functional and taxonomic levels. Nevertheless, trends discernible in a majority of infants indicate that gut colonization occurs in two distinct phases of succession, separated by the introduction of solid foods to the diet. This change in resource availability causes a sharp decrease in the taxonomic richness of the microbiota due to the loss of rare taxa (p = 2.06e-9), although the number of core genera shared by all infants increases substantially. Moreover, although the gut microbial succession is not strictly deterministic, we detect an overarching directionality of change through time towards the taxonomic and functional composition of the maternal microbiota. Succession is however not complete by the one year mark, as significant differences remain between one-year-olds and their mothers in terms of taxonomic (p = 0.009) and functional (p = 0.004) microbiota composition, and in taxonomic richness (p = 2.76e-37) and diversity (p = 0.016). Our results also indicate that the taxonomic composition of the microbiota shapes its functional capacities. Therefore, the observed inter-individual variability in taxonomic composition during succession is not fully compensated by functional equivalence among bacterial genera and may have important physiological consequences. Finally, network analyses suggest that positive interactions among core genera during community assembly contribute to ensure their permanence within the gut, and highlight an expansion of complexity in the interactions network as the core of taxa shared by all infants grows following the introduction of solid foods.

## Author Summary

Although knowledge of the complex community of microbes that inhabits the human gut is constantly increasing, the successional process through which it develops during infancy remains poorly understood. Particularly, although gut microbiota composition is known to vary through time among infants, the effect of this variability on the functional capacities of the community has not been previously explored. We simultaneously analyze the taxonomic and functional development of the gut microbiota in a birth cohort of healthy infants during the first year of life, showing that individual instances of gut colonization vary in their temporal dynamics and that clear parallelisms exist between functional and taxonomic change. Therefore, taxonomic composition shapes the functional capacities of the microbiota, and, consequently, successional variability may affect host physiology, metabolism and immunity. Nevertheless, we detect some overarching trends in microbiota development, such as the existence of two distinct phases of succession, separated by the introduction of solid foods, and a strong directionality of change towards the taxonomic and functional composition of the maternal microbiota. Understanding the commonalities and differences among individual patterns of gut colonization in healthy infants will enable a better definition of the deviations in this process that result in microbiota imbalances and disease.

## Introduction

The gastrointestinal tract (GIT) is a complex ecosystem where many factors, biotic and abiotic, play essential roles in reaching and maintaining a homeostatic equilibrium. The gut is endowed with the most diverse and dense microbiota of the human body, which plays fundamental roles in gut maturation, angiogenesis, immune system modulation, digestion, and protection from pathogens [1], [2]. Given such important roles for health, the inter-individual variability of the human gut microbiota in adulthood and at any stage of development still defies our expectations. This variability is shocking in light of the ecological assumption that community composition and dynamics respond to and are structured mostly by the environment, “everything is everywhere but the environment selects” [3], [4]. The GIT environment, although subject to inter-individual variation in diet and physiological parameters such as motility and transit time, presents a number of physical, chemical and mechanical properties that are mostly similar across individuals, including temperature, pH and surface tension values bound within limited ranges [5]. Consequently, we would expect a substantial degree of inter-individual convergence of GIT bacterial communities as a response to common selective pressures. Therefore, many studies have concentrated their efforts in the detection of a taxonomic core that would be shared by all individuals [6]–[10]. In that this search has been difficult, this view has recently evolved towards defining a few types of compositional profiles for the GIT microbiota. For example, Arumugam *et al.* [11] have stipulated that there are three universally distributed clusters of well-balanced host-symbiont states named enterotypes, driven mainly by bacterial composition, and that every individual's microbiota pertains to one of these enterotypes. However, the existence of such well-defined clusters of microbiota composition has been contested because their detection is highly dependent on the methodology employed. Instead, Koren *et al.* [12] propose that gut microbiota composition across individuals is better represented by a series of gradients of taxon abundances that result in a bimodal distribution, where the ends of the spectrum harbor markedly different relative abundances of taxa.

Moreover, theoretical and experimental community ecology indicate that different communities can assemble under identical selective pressures. With basis on the neutral theory of community ecology [13] and on the metacommunity concept [14], each human gut can be considered to harbor a local microbial community, the composition of which will be driven essentially by the stochastic processes associated with resampling from the metacommunity of all gut microbiotas to which it is linked by organismal dispersal. If there are limitations to the dispersal capacities of different species, such processes could result in the assemblage of substantially different communities in spite of the physical, chemical and mechanical characteristics shared by all guts. In addition, within the context of neutral theory, the functional equivalence hypothesis proposes that multiple species may possess similar functional attributes, and it has been shown that species-rich communities are particularly prone to the evolution of functionally equivalent species [15]. This functional equivalence hypothesis is appealing in regards to the inter-individual variation in composition of the GIT microbiota. Under functional equivalence, the taxonomically different assemblages in different individual guts could present similar overall functional profiles, so that the inter-individual variation would have no impact on the host. Metagenomic and metatranscriptomic functional studies indicate that this scenario is plausible, since, in contrast to taxonomic variability, there seems to be conserved functional profiles among the microbiotas of different individuals [8], [16]–[18]. Furthermore, this adds to the growing consensus that ecological community structure and function are better described by functional diversity (*i.e.* diversity of species traits [19]) rather than by taxonomic diversity [20], and that it is the alteration of functional diversity that will perturb the functioning of the ecosystem. The latter is further strengthened for microbial communities, as quantitative gene content analysis reveals specific fingerprints characterizing particular environments in spite of the substantial number of shared essential functions among bacteria [21].

In the case of human-associated microbiotas on which the host relies for specific functions, the alteration of functional diversity within the community can affect health status. Following this, several metagenomic studies have emphasized assessment of the functional diversity present in the GIT microbiota of healthy individuals, so as to be able to detect potential deviations in individuals affected by different diseases [2], [8], [9], [22]–[25]. Most of these efforts have concentrated on adult individuals, while the assessment of functional capabilities in the GIT microbiota of infants has remained underexplored. However, infancy is the critical period for gut microbiota assembly, during which a constant dialogue with immune and metabolic development is established. Consequently, epidemiological and experimental lines of evidence indicate that the microbe-host interactions set in place during infancy represent a main determinant of life-long health or disease [26]–[28]. Despite its importance, the process of gut microbiota development in infants is still poorly understood, and has been mostly surveyed at the level of taxonomic succession by means of culture or of molecular analyses based on the 16S rRNA gene. These studies have shown that the differential exposure of the infant to vaginal, fecal and skin bacteria from the mother depending on the mode of birth (*i.e.*, vaginal *vs.* C-section), as well as the type of feeding during the first months of life (*i. e.*, breastmilk *vs.* formula), are main factors influencing the richness, diversity and composition of the gut microbial community [29]–[32]; that the earlier stages of infant gut microbiota development are characterized by high levels of inter-individual variability and a very uneven distribution of taxa; and that, as infant development progresses, microbial assemblages converge towards an adult-like composition with a more even taxa distribution [6], [33], [34]. On the other hand, to date, functional diversity in infants has mostly been explored in cross-sectional studies [23], [25], and in a few longitudinal studies that have been limited to one [16], [35] or a handful of infants [18]. It is important to keep in mind that cross-sectional studies do not follow individuals through time, but rather reflect single snapshots of the microbiota of different individuals of varying ages, and, therefore, cannot inform on the extent of inter-individual variation in microbiota dynamics. Thus far, the functional capabilities of the microbiota in infants have been shown to broadly mirror those of the mother from very early on, in spite of large taxonomic differences, although functions such as vitamin biosynthesis and xenobiotic degradation increase with time. However, much remains to be learnt about the process of functional development of the microbiota during colonization of an infant's GIT.

Taking into account all of the above, the present study explores the patterns of taxonomic and functional change along time during GIT microbiota development in a birth cohort of 13 infants. With this aim, we have collected fecal samples from healthy infants throughout the first year of life, and have obtained metagenomic sequence to characterize the phylogenetic composition and genetic repertoire of the microbiota present in each sample. In addition, in order to assess the progression of the infant's microbiota towards an adult-like state, we have also collected and sequenced the microbiota present in the mother before and one-year after childbirth. Because we obtain both taxonomic and functional data, we can evaluate the functional development of the GIT microbiota and its interactions with taxonomic community assembly, in the context of the dietary and physiological changes that characterize the first year of life. Furthermore, because our analyses involve the prospective follow up of 13 infants, they allow us to evaluate several previously unexplored aspects of the GIT microbial succession process. Specifically, the availability of longitudinal data for several individuals sheds light on basic questions such as 1) whether taxonomic composition and functional development follow similar trends across individuals, 2) whether succession follows a strictly deterministic course, whereby early microbial assemblages set the stage for the next ones to come, 3) whether taxonomic variation among individuals during succession has an impact on the functional capabilities of the microbiota, and 4) whether community assembly is shaped by relationships of co-occurrence among taxa and how these evolve throughout succession. Overall, our data enable the characterization of microbial succession in the infant gut at unprecedented levels and, in particular, allow us to investigate whether the functional equivalence hypothesis can explain the inter-individual variability observed for this process.

## Results/Discussion

### Cohort, samples and sequencing

Given that our goal was to investigate the inherent variation in the process of microbial succession in the gut, rather than the specific alterations caused by factors such as type of delivery or infant feeding, we recruited to the study women having healthy pregnancies and stating their intention to exclusively breastfeed their infants during at least three months. We initially recruited 21 women, all residents of the city of Valencia, who were contacted during midwife visits. Due to various factors, we were able to obtain series of 4–5 infant fecal samples during the first year for only 13 of the enrolled women. At the moment of delivery, these women were between 29 and 42 years of age and had not taken antibiotics in at least three months before the onset of labor. Seven women received antibiotic during delivery and an eighth woman did so during the first week after. All 13 infants were born at term (>37 weeks of gestation), ten of them by vaginal delivery and three by C-section. Nine infants were exclusively breastfed during at least three months, three received a few formula feedings during the first days of life, and one was partially breastfed during the first month and formula-fed thereafter (Table 1). In addition to fecal samples, throughout the 12-months sampling period we obtained information regarding the infants' diet, general health and intake of antibiotics and other drugs (Table 1, Table S1), by means of specifically designed questionnaires that were given to the infants' parents. This information allowed us to establish that all infants remained healthy throughout most of the sampling period and that solid foods were introduced into their diets between the 3- and 7-months samplings, following patterns typical of Spanish Mediterranean infant diets [36].

**Table 1:** Information regarding mothers and infants obtained from questionnaires answered by the infants' parents.

Sample	Age	Sex	Delivery	Antibiotics Mother^a^	Antibiotics Infant	Diet
***MIP01*** **-MA**	29	-	-	No	-	-
***MIP01*** **-I1**	1 Week	Male	Vaginal	-	-	Breast milk
***MIP01*** **-I2**	1 Month	-	-	-	-	Breast milk
***MIP01*** **-I3**	3 Months	-	-	-	-	Breast milk
***MIP01*** **-I4**	7 Months	-	-	-	-	Solid foods
***MIP01*** **-I5**	1 Year	-	-	-	-	Solid foods
***MIP02*** **-MA**	36	-	-	No	-	-
***MIP02*** **-I1**	1 Week	Female	Vaginal	-	-	Mixed
***MIP02*** **-I2**	1 Month	-	-	-	-	Breast milk
***MIP02*** **-I3**	3 Months	-	-	-	-	Breast milk
***MIP02*** **-I4**	7 Months	-	-	-	-	Solid foods
***MIP02*** **-I5**	1 Year	-	-	-	-	Solid foods
***MIP03*** **-MA**	30	-	-	No	-	-
***MIP03*** **-I1**	1 Week	Female	Vaginal	Amoxicillin	Oftalmowell^b^	Breast milk
***MIP03*** **-I2**	1 Month	-	-	Amoxicillin	-	Breast milk
***MIP03*** **-I3**	3 Months	-	-	Cefuroxime	-	Breast milk
***MIP03*** **-I4**	7 Months	-	-	-	-	Solid foods
***MIP03*** **-I5**	1 Year	-	-	Amoxicillin	Cefuroxime	Solid foods
***MIP06*** **-MA**	42	-	-	Amoxicillin	-	-
***MIP06*** **-I1**	1 Week	Female	C-section	-	-	Breast milk
***MIP06*** **-I2**	1 Month	-	-	-	-	Breast milk
***MIP06*** **-I3**	3 Months	-	-	-	-	Breast milk
***MIP06*** **-I4**	7 Months	-	-	-	-	Solid foods
***MIP06*** **-I5**	1 Year	-	-	-	Amoxicillin	Solid foods
***MIP07*** **-MA**	31	-	-	Amoxicillin	-	-
***MIP07*** **-I1**	1 Week	Male	C-section	-	-	Breast milk
***MIP07*** **-I3**	3 Months	-	-	-	-	Breast milk
***MIP07*** **-I4**	7 Months	-	-	-	-	Solid foods
***MIP07*** **-I5**	1 Year	-	-	-	-	Solid foods
***MIP08*** **-MA**	30	-	-	No	-	-
***MIP08*** **-I1**	1 Week	Female	Vaginal	-	-	Breast milk
***MIP08*** **-I2**	1 Month	-	-	-	-	Breast milk
***MIP08*** **-I3**	3 Months	-	-	-	-	Breast milk
***MIP08*** **-I4**	7 Months	-	-	-	-	Solid foods
***MIP08*** **-I5**	1 Year	-	-	-	-	Solid foods
***MIP09*** **-MA**	30	-	-	No	-	-
***MIP09*** **-I1**	1 Week	Male	Vaginal	Amoxicillin	-	Mixed
***MIP09*** **-I2**	1 Month	-	-	Amoxicillin	-	Mixed
***MIP09*** **-I3**	3 Months	-	-	-	-	Formula
***MIP09*** **-I4**	7 Months	-	-	-	-	Solid foods
***MIP09*** **-I5**	1 Year	-	-	-	-	Solid foods
***MIP12*** **-MA**	31	-		Amoxicillin	-	-
***MIP12*** **-I1**	1 Week	Female	C-section	-	-	Mixed
***MIP12*** **-I2**	1 Month	-	-	-	-	Breast milk
***MIP12*** **-I3**	3 Months	-	-	Cefixime	-	Breast milk
***MIP12*** **-I4**	7 Months	-	-	-	-	Solid foods
***MIP12*** **-I5**	1 Year	-	-	-	-	Solid foods
***MIP13*** **-MA**	31	-	-	Benzylpenicillin	-	-
***MIP13*** **-I1**	1 Week	Male	Vaginal	Amoxicillin	-	Mixed
***MIP13*** **-I3**	3 Months	-	-	-	-	Breast milk
***MIP13*** **-I4**	7 Months	-	-	-	-	Solid foods
***MIP13*** **-I5**	1 Year	-	-	-	-	Solid foods
***MIP16*** **-MA**	39	-	-	Amoxicillin	-	-
***MIP16*** **-I1**	1 Week	Male	Vaginal	-	-	Breast milk
***MIP16*** **-I2**	1 Month	-	-	-	-	Breast milk
***MIP16*** **-I3**	3 Months	-	-	-	-	Breast milk
***MIP16*** **-I4**	7 Months	-	-	-	-	Solid foods
***MIP16*** **-I5**	1 Year	-	-	-	-	Solid foods
***MIP17*** **-MA**	39	-	-	No	-	-
***MIP17*** **-I1**	1 Week	Male	Vaginal	-	-	Breast milk
***MIP17*** **-I3**	3 Months	-	-	-	-	Breast milk
***MIP17*** **-I4**	7 Months	-	-	-	-	Solid foods
***MIP17*** **-I5**	1 Year	-	-	-	-	Solid foods
***MIP19*** **-MA**	33	-	-	No	-	-
***MIP19*** **-I1**	1 Week	Female	Vaginal	-	-	Breast milk
***MIP19*** **-I3**	3 Months	-	-	-	-	Breast milk
***MIP19*** **-I4**	7 Months	-	-	-	-	Solid foods
***MIP19*** **-I5**	1 Year	-	-	-	-	Solid foods
***MIP21*** **-MA**	35	-	-	Amoxicillin	-	-
***MIP21*** **-I1**	1 Week	Male	Vaginal	-	-	Breast milk
***MIP21*** **-I2**	1 Month	-	-	-	-	Breast milk
***MIP21*** **-I3**	3 Months	-	-	-	-	Breast milk
***MIP21*** **-I4**	7 Months	-	-	-	-	Solid foods
***MIP21*** **-I5**	1 Year	-	-	-	-	Solid foods

MIP: Mother Infant Pair.

a For MA samples we report whether antibiotics were given during childbirth and the specific antibiotic given. In the case of C-sections, we report administration of amoxicillin, which is the standard practice in Spanish hospitals. None of the mothers had taken antibiotics before childbirth for at least three months.

b Oftalmowell is a combination of gramicidin, neomycin and polymyxin B.

Infant samples were collected at one week (I1), one month (I2), three months (I3, before introduction of solid foods), seven months (I4, after introduction of solid foods) and one year after birth (I5), and maternal samples were collected within one week prior to delivery (MA) and one year after (MB). We obtained 13 samples at each infant and maternal timepoint except for I2, for which only 9 samples were available, for an overall total of 87 samples that were processed for metagenomic pyrosequencing. After quality filtering, we obtained a total of 5,500,784 reads with a mean of 64,119 reads per sample and an average length of 348 bp (range 263–446 bp). For many reads, more than one Open Reading Frame (ORF) was recovered with a total of 9,968,776 ORFs and an average of 114,584 ORFs per sample. Annotation allowed for taxonomic assignment of 9,014,059 ORFs (103,610 per sample) and functional assignment of 675,141 ORFs (7760 per sample). Sequencing and annotation details as well as abundance tables for taxa and functions on a per sample basis are provided in the Supporting Information (Table S2, Table S3, Table S4). All sequences have been deposited in the IMG/M database [37] under the project name “Gut Microbiota of Spanish Mother-Infant Pairs”.

### The maternal microbiota changes between the perinatal period and one year after childbirth

**Figure 1:**
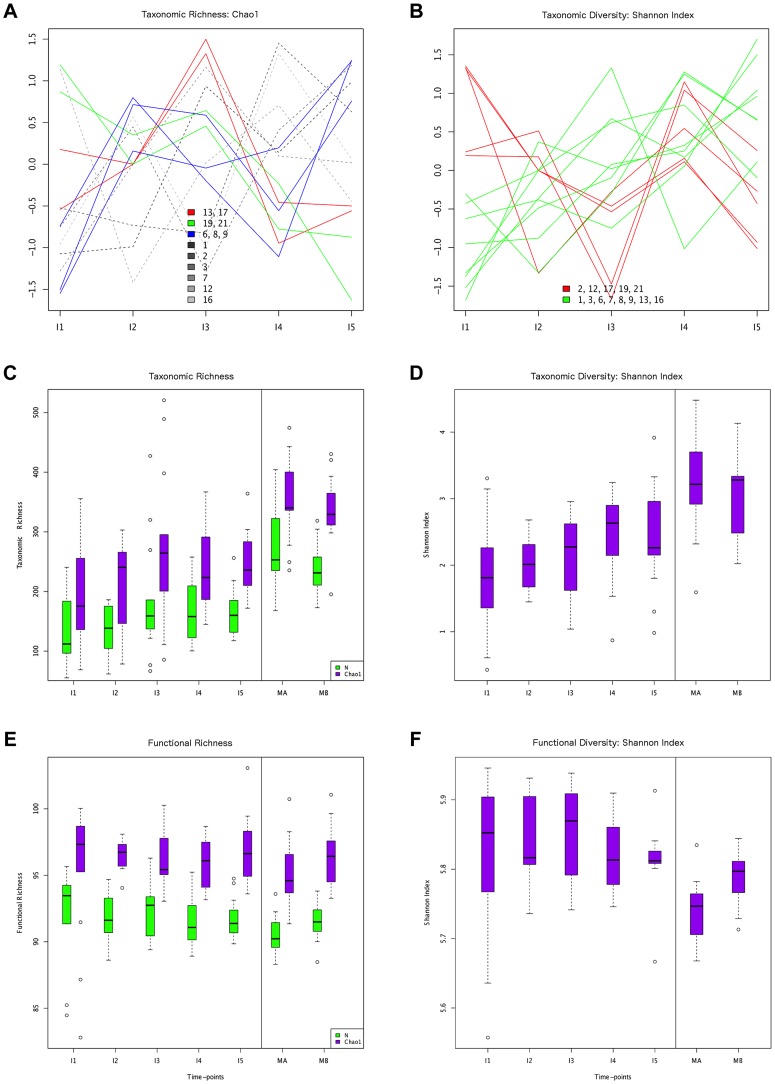
Different behaviors of taxonomic and functional richness and diversity through infant gut microbiota development. Hierarchical clustering of temporal profiles for (A) taxon richness (Chao1 estimator) and (B) taxon diversity (Shannon index), showing the extent of variation among the 13 infants. Values are centered at the mean of all samples and scaled by the standard deviation. Colored profile clusters have >95% support based on multiscale bootstrap resampling. The boxplots in (C) and (D) summarize the general behavior of taxon richness and diversity for all infants. Taxon richness (C) shows an increase in median values with time interrupted by the introduction of solid foods (I4), when a decrease in richness is observed. Taxon diversity (B) shows an increase in median values from I1 to I4 followed by a decrease between I4 and I5. Functional richness (E) and diversity (F) show no specific pattern but rather fluctuate with time.

**Figure 2:**
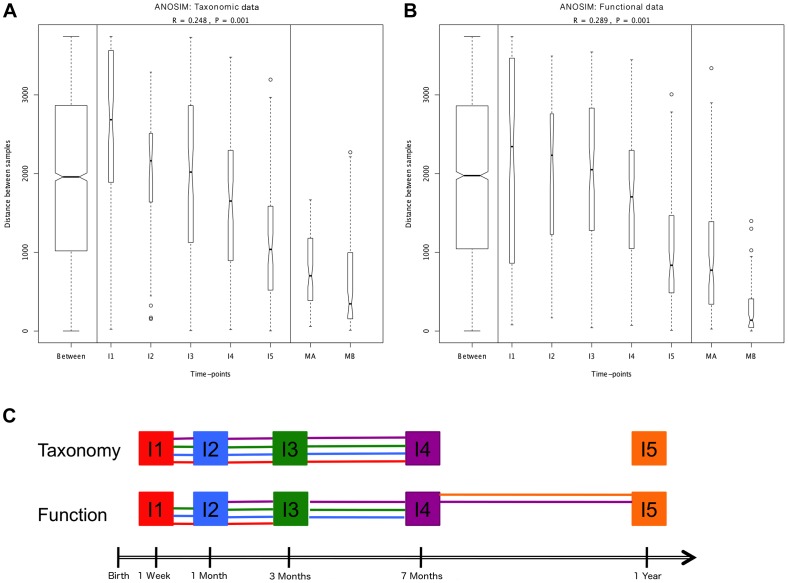
ANOSIM comparison of timepoints. Overall analyses for taxonomic (A) and functional (B) Bray-Curtis distances among all samples. The length of the bows indicates the level of heterogeneity and the width the number of compared samples. Statistically significant differences among timepoints are detected for both taxonomic and functional data. Note the decrease in heterogeneity with time in infants and the larger heterogeneity in MA compared to MB samples. (C) Representation of pairwise ANOSIM analyses between timepoints. Each timepoint is represented by a color and is linked by lines of this color to all timepoints from which it is not significantly different. For functional composition, significant differences appear between timepoints that are more separated in time, indicating directionality along infant development, but no such pattern is detected at the taxonomic level.

Several changes are detected between the mother's gut microbiota days before childbirth and that present one year later. MA samples show a higher taxonomic richness (p = 0.002), due to a higher representation of rare taxa (abundance under 1% in all samples), but their functional diversity is lower (p = 0.009), indicating that they are functionally more redundant than MB samples (Figure 1C–1F). In addition, in clustering analyses based on similarity of microbiota composition, arbitrary clustering patterns are obtained where the MA and MB samples of the same woman do not group together, neither for taxonomic nor for functional composition (Figure S1). MA samples also present a larger range of inter-individual variability at both the taxonomic and functional composition levels (Figure 2A, 2B). These changes suggest a decrease in the host's capacity to regulate microbiota composition and function during late pregnancy, perhaps related to the low-grade inflammation of GIT mucosal surfaces and to the other immune, physiologic, hormonal and metabolic changes that occur during this period. Moreover, our results are in agreement with the recent demonstration that the maternal gut microbiota is dramatically altered between the first and third trimesters of pregnancy [38].

The composition of the maternal GIT microbiota during the perinatal period could be of great importance to the microbial colonization of the infant. Although the *in utero* environment has been considered sterile under normal conditions [30], culture-dependent and 16S rRNA gene pyrosequencing analyses have detected microorganisms in human meconium, amniotic fluid and umbilical cord, even when no rupture of membranes has occurred and in elective Cesareans [39]–[44]. The suite of changes that occur during late pregnancy [45], [46] may facilitate the transport of maternal bacteria to the fetal GIT. In mice, translocation of live intestinal bacteria to mesenteric lymph nodes increases in late pregnancy [47], [48], and dendritic cells have been shown to mediate increased bacterial translocation from the gut to blood and adipose tissue in obesity and diabetes [49], conditions similar to late pregnancy in terms of metabolic changes and the presence of a low-grade inflammatory state. Following translocation, intestinal bacteria could be transported in a controlled manner through lymph and blood, potentially reaching sites from which they could be transferred to the offspring, such as the placenta and the mammary glands. In support of this possibility, 16S rRNA gene pyrosequencing has detected very similar communities of organisms in meconium and in colostrum [41], [50]. The taxonomic composition of these communities, which are most often dominated by Lactic Acid Bacteria (LAB), does not correspond to the bacterial abundances in maternal perinatal fecal samples [41], suggesting that the mother is able to regulate which bacteria reach the fetus, and/or that a restricted set of bacteria can survive in the fetal GIT to serve as a first inoculum and initiate the GIT colonization process.

### Early colonizers and role of maternal transmission in the initial development of the GIT microbiota

The taxonomic composition detected in infants at the first timepoint analyzed, I1, is shown in Figure S2A. At this timepoint, the GIT microbiota of different infants is quite divergent, since in each one of them a single genus dominates extensively. *Bacteroides* dominance is the most prevalent, being detected in 5 of the neonates, followed by *Clostridium* (3 neonates), *Veillonella* (2 neonates), *Bifidobacterium* (2 neonates), and *Escherichia* (1 neonate). Among the 9 infants who were born vaginally and were breastfed exclusively (MIPs —Mother-Infant Pairs— 1, 3, 8, 16, 17, 19 and 21) or received a little amount of formula early on (MIPs 2 and 13), all five dominance patterns can be found, although *Bacteroides* is the most common. *Bifidobacterium* dominates in one exclusively breastfed infant (MIP17) and in the infant who was only partially breastfed (MIP9), both of whom were vaginally born. On the other hand, the three infants born by C-section had I1 microbiotas dominated by a Firmicutes genus, *i. e.*, *Clostridium* (MIPs 6 and 12) or *Veillonella* (MIP7). This is in agreement with previous studies indicating that C-section delays the establishment of *Bacteroides*, *Bifidobacterium* and *E. coli* [31], [51]. The Canonical Correspondence Analysis (CCA) in Figure S3 shows that C-section does influence the taxonomic composition of the infant microbiota at I1, although it only explains 16% of the total variability. Antibiotic use during delivery and supplementation of the infant's diet with formula (Table 1) play a more limited role, as they explain 11% and 7% of the total variability at this timepoint, respectively (Table S5).

The five genera that dominate the I1 microbiota in different neonates may have had an important head start for GIT colonization, as all of them have been identified in meconium, although they were not the most common taxa revealed by 16S rRNA pyrosequencing in term infants [41]. Moreover, we have previously shown that the meconia passed by two of the infants in this cohort (MIPs 2 and 21) contain 16S rRNA gene sequences, including sequences from *Bacteroides* and *Clostridium*, that are also recovered at 100% identity from the corresponding maternal samples and infant samples from different timepoints [41]. This suggests that these bacteria can be acquired *in utero* and then maintained in the infant for long periods of time. In addition, here we detect that one-week-old infants share a substantial, but highly variable among individuals, percentage of GIT microbiota genera with their respective mothers prior to giving birth (between 26% and 88%, average 71%). These taxa could have been acquired *in utero*, during delivery or through breast milk.

The early colonizers of a given environment can have crucial consequences for the further development of the community. Theoretical models of succession differ on whether they consider that those organisms able to establish themselves in a long-term manner in a given environment will be able to colonize it from the start, or, rather, that early succession will be dominated exclusively by “opportunists” or “pioneers” adapted to the transient conditions common to all recently opened spaces. Pioneers are expected to have cosmopolitan distributions, broad dispersal and rapid growth capabilities in order to arrive first and quickly occupy an empty space [52], [53]. Most of the genera that we find dominating at I1 hardly correspond to this definition. Except for *Clostridium* and *Escherichia*, the remaining genera (*Bacteroides*, *Veillonella* and *Bifidobacterium*) are intermediate or slowly growing species with known optimal generation times ranging from one to three hours [54], [55]. Moreover, their metabolism is strictly anaerobic, their environmental distribution is not cosmopolitan but host-associated [56], and they can be found at high abundances in later stages of succession. These observations suggest that these organisms are not opportunists taking advantage of a newly available habitat, but rather GIT-specialists, highly competitive in this particular environment. Therefore, the GIT microbial succession does not seem to follow a “facilitation” model, in which pioneers colonize an open space and create the necessary conditions for more specialized late-coming organisms [52]. Although it is possible that a facilitation phase may have taken place at a very rapid pace during the first days after birth, it is still noteworthy that, with the exception of one infant whose microbiota consisted almost exclusively of *Escherichia* and other enterobacteria, all infants at I1 had a microbiota that was already dominated by a strict anaerobe, contrary to the common assumption that early colonizers must be facultative anaerobes [57]. Rather, it suggests that anaerobic conditions are quickly established, and that the strict anaerobes have strong competitive advantages that allow them to rapidly dominate over any facultative anaerobes that could have been present during the very first days after birth, such as the vaginal *Lactobacillus* acquired through the birth canal [29].

If the I1-dominating genera were present in the GIT before the moment of birth, even if at very low abundances, a rapid expansion may have occurred as soon as conditions became favorable. The start of breastfeeding should select for organisms able to grow in its main constituents, such as lactose and Human Milk Oligosaccharides (HMOs). These oligosaccharides are the main growth factors for *Bifidobacterium* [58], but recent work has shown that they can also sustain the efficient growth of *Bacteroides* [59]. Remarkably, although *Bacteroides* has often been reported to be uncommon during the neonatal period, we detected a microbiota dominated by this genus in five of the 13 one-week-old infants. In fact, it should not be surprising that *Bacteroides* might quickly establish, given that it is the only genus besides *Bifidobacterium* known to efficiently grow on HMOs and that it is also one of the most efficient utilizers of the mucin molecules that line the intestinal epithelium [60]. Many species of *Bifidobacterium*, *Escherichia* and *Clostridium* can also utilize mucin, in addition to lactose [61]. *Veillonella*, on the other hand, can't metabolize carbohydrates and requires short-chain fatty acids (SCFA), such as lactate or pyruvate, for growth [62]. Its dominance in two of the one-week-old infants suggests that a short food chain had already been established whereby *Veillonella* could have access to SCFA produced by other GIT genera, for instance by lactose fermentation. In this regard, it can be noted that, in the infants having a high abundance of *Veillonella*, genera that can ferment lactose to SCFA, such as *Clostridium* or *Streptococcus*, were indeed also abundant.

### Dynamics of taxonomic and functional richness and diversity during the first year of life

In order to characterize the dynamics of richness and diversity in the infant microbiota from the first week to the one-year mark, we computed the Chao1 estimator [63] and the Shannon index [64], for both taxa and functions (Table S6). Chao1 estimates richness, *i. e.*, the number of taxa or functions present in a community, whereas the Shannon index of diversity takes into account both richness and evenness, *i. e.*, how similar the abundances of the different taxa or functions are. The dynamics of taxon richness along time are presented in Figure 1 for individual infants (A) and across all individuals (C). Chao1 values increase overall between I1 and I5 (p = 6.18e-18), an increase that is present in most of the infants. However, the increase is not linear (linear regression p = 0.205, Figure S4A), nor continuous. In most infants, richness is under two thirds of the maternal value (MB) at I1, and then increases from I1 to I2. Although change across all infants is not significant for this first interval (p = 0.139), the tendency to increase is reflected in median values (Figure 1C). In the I2–I3 interval, even though richness increases or decreases in similar numbers of infants, overall it is higher at I3 than at I1 (p = 2.33e-38) and I2 (p = 3.70e-24), partly due to the presence of three outliers having very high I3 values (MIPs 7, 13 and 17). Then, from I3 to I4, the interval in which solid foods were introduced, most infants present a decrease in richness, which is significant across individuals (p = 2.06e-09). This decreasing trend may or may not reverse from I4 to I5, so that the change in this interval does not reach significance (p = 0.107) and richness values at I5 remain significantly lower than those that had been attained by I3 (p = 1.13e-05), before the introduction of solid foods. Richness at I5 is also significantly lower than that of the mothers (p = 2.76e-37 *vs.* MB), although by this final timepoint most infants have already surpassed two thirds of the MB richness value. Hierarchical clustering analysis of the temporal profiles of richness change for individual infants retrieves three significant clusters, one including infants 6, 8 and 9, another including infants 13 and 17, and a last cluster including infants 19 and 21 (Figure 1A). These clusters do not associate with delivery type, antibiotic use or formula supplementation.

The taxon diversity changes undergone by the different infants during the year are as variable as those seen for taxon richness, but some trends can also be discerned (Figure 1B, 1D). These trends mirror the behavior of richness in some time intervals, but not in others. As seen for richness, taxon diversity increases significantly between I1 and I5, and, in this case, regression analysis indicates that the increase can be considered linear when this entire period is considered (p-value = 0.024; Figure S4B). The pattern of change is similar to that of richness throughout the first three months; however, trends that are opposite to those observed for richness are present after the three months mark, as, in most cases, the Shannon index increases in I3–I4 and decreases in I4–I5. In terms of median values, there are increases between all consecutive timepoints except I4–I5, when the median diversity decreases to a value similar to that attained by I3 (p = 0.053; Figure 1D). By I5, taxon diversity is still significantly lower than that of MB (p = 0.016) but most infants have reached a Shannon index value that surpasses two thirds of that of their mother, a situation that is again comparable to that of taxon richness. Hierarchical clustering groups the diversity temporal profiles of the infants into two significant clusters, one including infants 2, 12, 17, 19 and 21, and the other, in which the trend towards a linear increase in diversity with time is more pronounced, including infants 1, 3, 6, 7, 8, 9,13 and 16 (Figure 1B). Clustering patterns differ then for taxonomic richness and diversity, with only infants 6, 8 and 9, on one hand, and infants 19 and 21, on the other, clustering together for both parameters.

The opposite trends in taxon richness and diversity from I3 to I5 suggest that changes in richness correspond to the appearance and disappearance of rare taxa, which, if substantial, would respectively result in lower and higher degrees of evenness in the distribution of taxa abundances in the community, captured in the Shannon diversity index. Indeed, rank abundance curves confirm that richness changes are driven mainly by the removal of rare genera in I3–I4, followed by the addition of different rare genera in I4–I5 (data not shown).

Regarding functional richness and diversity in the infant samples (Figure 1E, 1F), their behavior is in sharp contrast to that observed at the taxonomic level, as these functional parameters fluctuate through time across a relatively narrow range of values, with no clear trends to increase or decrease along development (Table S6, Figure S4C, S4D). In fact, even at the earliest sample collection times, the median values of functional richness and diversity in infants are already similar or higher than those obtained for the mothers, particularly in the perinatal samples (MA), which present the lowest values. This indicates that the infant microbiota attains a level of functional complexity similar to that of the mothers from very early on, possibly due in part to the general presence of essential bacterial functions and of those specifically needed for survival in the gut environment.

### Succession in infants does not follow a strictly deterministic course

Whether ecological successions are deterministic processes is still a matter of contention. In microbial communities, this question has rarely been explored. Our prospective cohort analysis enables us to address this issue in several complementary ways. We have already described the lack of a common successional pattern across individuals in terms of the magnitude and direction of taxon richness and diversity changes between timepoints. Clustering analysis of individual samples using the Bray-Curtis distance [65] provides other means of assessing the degree of determinism in the infants' successional paths. Firstly, we analyzed the clustering patterns within each MIP to determine whether they all share the same topology. For both taxonomic and functional composition, MIPs have seemingly idiosyncratic clustering patterns (Figure S5). The most marked tendency is the grouping of I5 with maternal samples, observed in 31% and 62% of the MIPs at the taxonomic and functional levels, respectively, independently of the mode of birth. The lack of a common clustering pattern across the different MIPs reinforces the notion that the infants' successional paths follow non-deterministic dynamics, although a trend of convergence towards the maternal functional composition by the end of the year is suggested.

**Figure 3:**
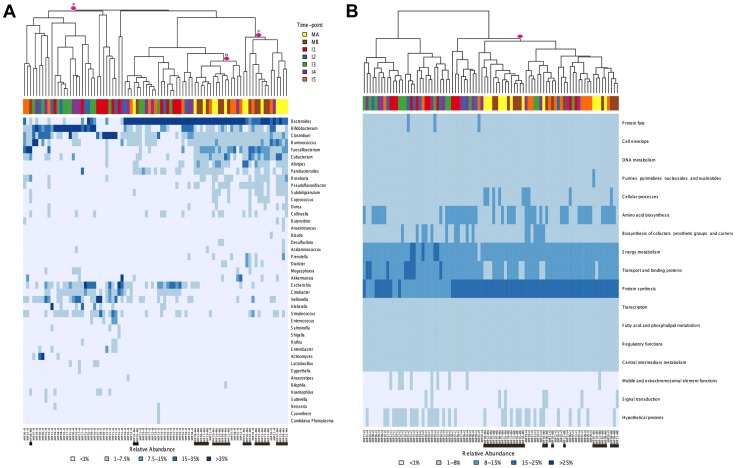
Heatmaps and clustering of individual gut microbiota samples for taxonomic (A) and functional composition (B). Clustering was based on Bray-Curtis distances. (A) Only the genera above 1% abundance in at least one sample are depicted. (B) Functional composition was established based on TIGRFAM main functional roles. Each sample is identified at the bottom of the heatmaps by a code that specifies the MIP to which it belongs and the corresponding timepoint. Maternal samples are additionally highlighted by means of black bars. Colors on top of each heatmap represent the timepoints to which samples belong. Pink circles identify specific clusters referred to in the text.

Global comparisons of all infant and maternal samples at the taxonomic and functional levels also point in this direction. Such comparisons reveal no clear pattern of sample clustering, neither by individual nor by timepoint (Figure 3A–B). The fact that samples from the same timepoint do not cluster together indicates that the microbiota present at each timepoint can not be defined as a well-differentiated, discrete and predictable community, such as the seral communities postulated in some models of vegetational succession [66]. Nevertheless, some degree of unevenness can be observed in the distribution of samples across clusters, pointing towards an effect of age on microbiota composition. The taxonomic heatmap in Figure 3A shows a large cluster (a) that contains 30 infant samples but only one maternal sample, as well as two clusters (b and c) that contain nearly all of the maternal samples and some I4–I5 infant samples. A similar effect can be seen in the functional heatmap (Figure 3B), where a single cluster contains the 26 maternal samples, most of the I5 samples and only a few of the samples from other infant timepoints. In other words, as in the MIP-based analyses, I5 shows here a clear tendency to cluster with the maternal samples, for both taxonomy and function.

Finally, comparison of the heatmaps corresponding to each timepoint in the series (Figure S2) enables us to evaluate whether early microbial assemblages determine the nature of the next ones to come. Rather, it can be seen that the patterns of association among samples from different individuals change through time. For instance, infants 2 and 16 have very similar taxa composition profiles at I1, while they differ widely at all subsequent timepoints. Conversely, infants 17 and 19 are the most similar one-year-olds, whereas at earlier timepoints they had microbiotas dominated by *Bifidobacterium* and *Bacteroides*, respectively. The varying patterns of association among samples through time indicate that early similarity among infants does not predict similar developmental paths or one-year mark outcomes.

### Is there directionality in taxonomic and functional change along development?

We next set out to investigate whether, in spite of the lack of determinism in successional paths, an overall pattern of directional change through time towards an adult-like microbiota can be discerned, as suggested by global and MIP-based clustering analyses. To this aim, we employed several multivariate analyses based on the Bray-Curtis distances among samples. We first examined whether there are significant overall differences among the entire set of analyzed timepoints. Comparison of distances between and within timepoints revealed that significant differences exist at both the taxonomic and functional levels (ANOSIM: taxonomic R = 0.30, p = 0.001 & functional R = 0.27, p = 0.001). The plots in Figure 2A–B display the amount of variation among samples within and between timepoints and allow us to appreciate the wider divergence between samples in earlier timepoints and the progressive increase in homogeneity as the gut microbiota develops, as previously noted [6]. This can be considered a first clearly directional trend in the data, observable at both the taxonomic and functional levels. We then performed a series of pairwise ANOSIM analyses in order to detect where the main differences among timepoints lie. All infant samples, including those from the one-year timepoint, are distinguishable from maternal samples both by taxonomy and by function (Table S7). The results obtained for comparisons between infant samples are illustrated in Figure 2C, where each timepoint is represented by a color and is linked by lines of this color to all timepoints from which it does not differ significantly. At the taxonomic level, no pairwise comparison for timepoints I1 to I4 identifies significant differences, whereas each of these timepoints is distinguishable from the I5 timepoint. In other words, this analysis reveals no progressive increase in taxonomic composition distance along time. In contrast, at the functional level, although none of the infant timepoints is significantly different from its immediate neighbor, differences become significative for timepoints that are separated by one or two intermediate timepoints. That is, in this case, larger functional differences appear between timepoints that are more separated in time, indicating a clear directionality along infant development. Nevertheless, this type of analysis does not show a progression in microbiota composition towards the adult state, as all infant timepoints remain distinguishable from those of the mothers, for both taxonomy and function.

**Figure 4:**
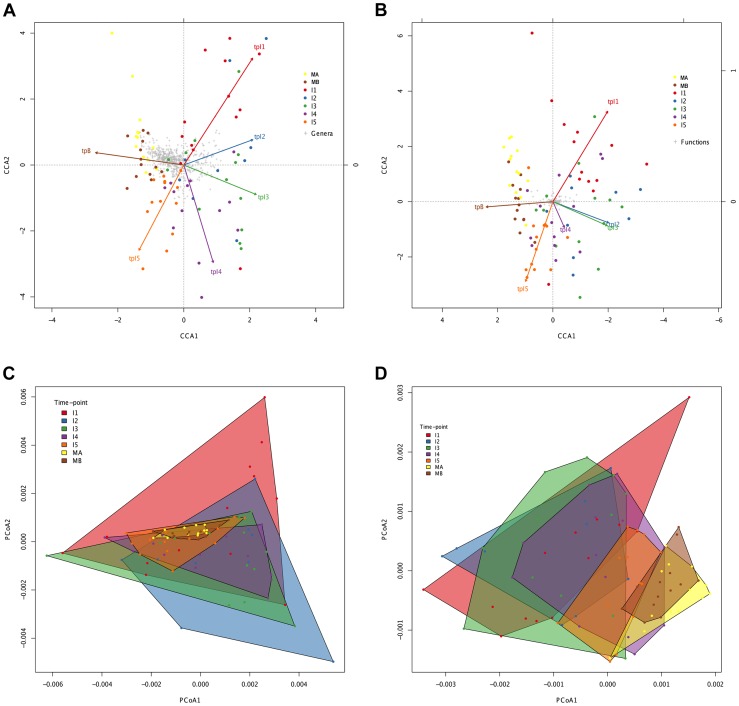
Directionality in taxonomic and functional change through time. Canonical Correspondence Analysis (CCA) of taxonomic (A) and functional (B) data, showing that the main axis (CCA1) separates infant timepoints I1, I2, I3 and I4 from I5, MA and MB. The percent variation explained by the main axis is 60.22% in A and 81.57% in B, while CCA2 explains 14.20% variation in A and 6.99% in B. The direction of the timepoint arrows indicates the main axis of deviation from the reference maternal timepoint (MA). Taxonomic (C) and functional (D) Principal Coordinates Analyses (PCoA) depicting convex hulls enclosing all samples pertaining to a determined timepoint. The percent variation explained by the main axis is 46.60% in C and 30.28% in D, while PCoA2 explains 23.00% variation in C and 16.04% in D. Heterogeneity within timepoints is represented by arrow length (CCA) or convex hull area (PCoA). All analyses identify a progressive change from timepoint to timepoint with clear directionality towards the composition of the mothers.

In order to further visualize how the compositional variation among samples is distributed, we performed multivariate statistical techniques that provide the coordinates of the samples in a reduced space representing the main variation components. In contrast to the ANOSIM analyses, Canonical Correspondence Analyses (CCA; Figure 4A, 4B) identify a progressive change from timepoint to timepoint with clear directionality towards the adult state for both taxonomy and function. The taxonomic and functional CCAs recover the same pattern, with a slight difference in terms of the proximity between timepoints I2 and I3, which are closer for the functional data set. Although discrete clusters of samples by timepoint are not present, the CCA plots show an orderly displacement from I1 to I5, clearly observed in the changing direction of the timepoint arrows, which indicates the main axis of deviation from the reference maternal timepoint (MA). Moreover, in both cases the first axis of the CCA graph separates the majority of infant samples (I1, I2, I3 and I4) from the one-year-old and maternal samples (I5, MB and MA), indicating that progressive change throughout the first year has resulted in a microbiota that is more similar to that of the mothers. We also analyzed the taxonomic and functional datasets with Principal Coordinates Analyses (PCoA) performed on matrices of Gower distances [67], followed by the drawing of convex hulls enclosing all samples pertaining to a particular timepoint [68]. It can be seen in Figure 4C and 4D that, for both taxonomy and function, there is a general decrease of the area of the convex hulls with age, indicating again a decrease in heterogeneity among coetaneous samples, as well as a time-ordered displacement of the infants' convex hulls towards those of the mothers. We calculated the taxonomic and functional dissimilarities between two timepoints by estimating the non-overlapping areas of their convex hulls (Table S8). As expected, in both cases dissimilarity is lowest between maternal samples and between infant timepoints that are close in time, and is at its peak when I1 convex hulls are compared to the maternal ones. So, both CCA and PCoA coincide in showing a clear time-ordered displacement of taxonomic and functional composition whereby each successive infant timepoint becomes more similar to the mothers.

However, the convex hulls in Figure 4C and 4D point out an interesting difference between taxonomic and functional compositional change. In the case of taxonomic composition, the maternal convex hulls are enclosed within the space occupied by the infant timepoints, which seem to close in around the maternal hulls as time progresses. In contrast, in the case of function, the maternal hulls occupy the rightmost part of the graph and the infant samples progressively shift in that direction, so that some degree of overlap with the maternal hulls is only observed from the I3 timepoint onwards. This suggests that the GIT microbiota undergoes a more pronounced directional shift during succession at the functional than at the taxonomic level.

### Parallelisms between taxonomy and function counter the functional equivalence hypothesis

**Figure 5:**
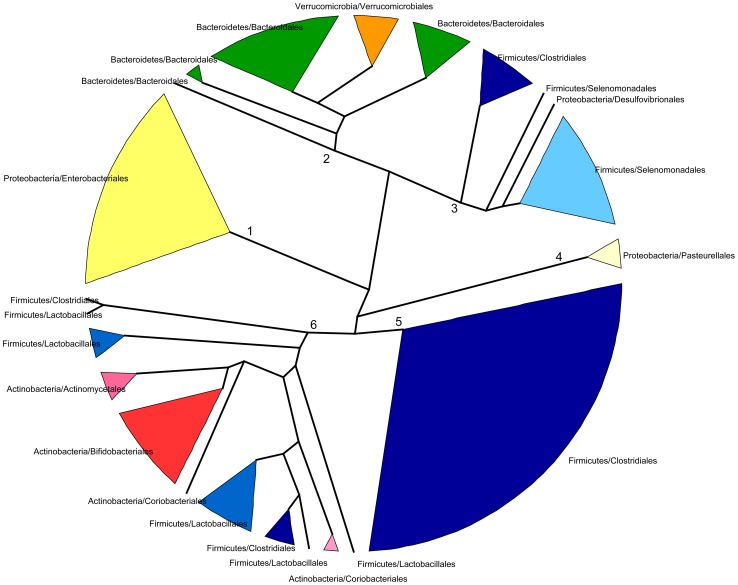
Dendrogram showing six main groups of gut microbiota genera based on functional profile clustering. Functional profiles were defined as the relative abundances of TIGRFAM subroles in a given genus. Only genera present in any sample at >1% abundance and having genes representing at least 50% of the 108 subroles detected in our complete data set were included. Clustering was based on the complete linkage method applied to a matrix of pairwise Bray-Curtis distances between the functional profiles of genera. Branches in the resulting dendrogram were collapsed when genera on the tips pertained to the same order. Orders of the same phylum have different shades of the same color.

In spite of some differences, we have just shown that the changes in taxonomic and functional microbiota composition with time are similar both in terms of the directionality of change toward the maternal profile and of the progressive reduction of heterogeneity among individual samples. This argues for an effect of the taxonomic composition of the microbiota on its functional gene repertoire. In order to further investigate the relationship between taxonomy and function, we analyzed the functional similarities among GIT microbiota genera. For this, we determined and compared the functional profiles of all genera that reached 1% abundance in at least one sample. Because not enough information was available in a sample per sample basis for each genus, functional profiles were established after pooling all samples for a given timepoint. Functional profiles were defined as vectors containing the relative abundances of each TIGRFAM subrole within a particular genus and timepoint. We then constructed a dendrogram clustering genera by functional profile similarity as measured by the Bray-Curtis distance (Figure 5). The resulting dendrogram mainly follows phylogenetic relationships, suggesting that each phylogenetic group has a characteristic set of functional profiles. At the genus level, the functional profiles computed for the different timepoints generally form an exclusive group, suggesting that either the same species of the genus are present along development or that all members of the genus share similar sets of genes. Moreover, clustering by phylogenetic affiliation also occurs at higher taxonomic ranks, as functional groups comprising only members of specific families and orders are recovered. Six major functional groups are obtained: Group 1, enclosing all Enterobacteriales; Group 2, enclosing all Bacteroidales and Verrucomicrobiales; Group 3, comprising all Selenomonadales, plus the Clostridiales genera *Pseudoflavonifractor* and *Subdoligranulum* and the δ-proteobacteria *Desulfovibrio*; Group 4, enclosing all Pasteurellales; Group 5, comprising most of the Clostridiales, and Group 6, enclosing the Clostridiales genera *Anaerostipes* and *Faecalibacterium*, the Lactobacillales and all Actinobacteria. Interestingly, only members of the phyla Firmicutes (Clostridiales, Lactobacillales and Selenomonadales) and Proteobacteria (Enterobacteriales, Pasteurellales and the genus *Desulfovibrio*) are present in multiple major functional groups. In particular, the order Clostridiales is the most functionally diverse, as it is the only order split into several of the major groups, even though a large majority of genera are found in functional group 5.

Although the general topology of the dendrogram in Figure 5 implies that the functional profile of taxa is strongly related to phylogenetic affiliation, some particular groupings indicate that functional convergence may occur among distantly related taxa. Most remarkable is the clustering in functional group 6 of the Bifidobacteriales and other less abundant Actinobacteria with the Firmicutes order Lactobacillales, which comprises the Lactic Acid Bacteria (LAB). Bifidobacteria are known to share many metabolic properties with the LAB, notably the production of lactic acid as a main endpoint of carbohydrate fermentation. In addition, group 6 also contains two Clostridiales genera, the acetate-requiring butyrate-producers *Faecalibacterium* and *Anaerostipes*. *Faecalibacterium* can also produce lactate, whereas *Anaerostipes* rather consumes it to produce butyrate. Another interesting grouping is that of the Verrucomicrobiales and the Bacteroidales, mainly represented by *Akkermansia* and *Bacteroides*, two genera that share important metabolic functions in the gut, as both are acetate and propionate producers and highly adept at mucin degradation [60].

Nevertheless, the observed groupings among phylogenetically distant taxa do not indicate functional equivalences that could account for the inter-individual variation in patterns of taxon dominance. This is most evident for timepoint I1, in which the taxonomic discrepancy among samples is maximal and the microbiotas of each individual are mostly dominated by a single genus (Figure S2A). Under the functional equivalence hypothesis, we would expect that the most abundant taxa present in the different samples would have similar functional profiles, independently of their phylogenetic lineage affiliation, and would cluster together into specific functional groups. Rather, the five genera that dominate the microbiota in different I1 infants (*Bacteroides*, *Clostridium*, *Veillonella*, *Bifidobacterium* or *Escherichia*) are found in deeply separated groups of the functional profile tree. This suggests that their functional capabilities are vastly different, and therefore that functional similarity and the functional equivalence hypothesis can't explain their presence as dominating taxa in the microbiotas of different infants.

### Dynamics of specific taxa and functions along development

Figures 3A and S2A show that, overall, the infants' samples can have high abundances of bacteria such as *Escherichia*, *Citrobacter*, *Bifidobacterium*, *Veillonella* and *Streptococcus*, in addition to *Clostridium* and *Bacteroides*, which are also common in adults. Venn diagrams allowed us to visualize details of the dynamics of taxa acquired or lost at each particular timepoint and of those that were maintained throughout the whole process of development. We identified a small core of ten genera that are present at all timepoints, in all infants and adults, although at very different abundances, comprised of *Bacillus*, *Bacteroides*, *Clostridium*, *Enterococcus*, *Escherichia*, *Eubacterium*, *Lactobacillus*, *Prevotella*, *Streptococcus* and *Vibrio*. Of note, this global core of 10 genera includes members of four of the functional groups defined above (Group 1: *Escherichia*; Group 2: *Bacteroides* and *Prevotella*; Group 5: *Clostridium* and *Eubacterium*; and Group 6: *Enterococcus*, *Lactobacillus* and *Streptococcus*). *Bacillus* (order Bacillales) and *Vibrio* (Vibrionales) are not represented in the functional profiles dendrogram because their low abundances precluded the computation of reliable functional profiles.

**Figure 6:**
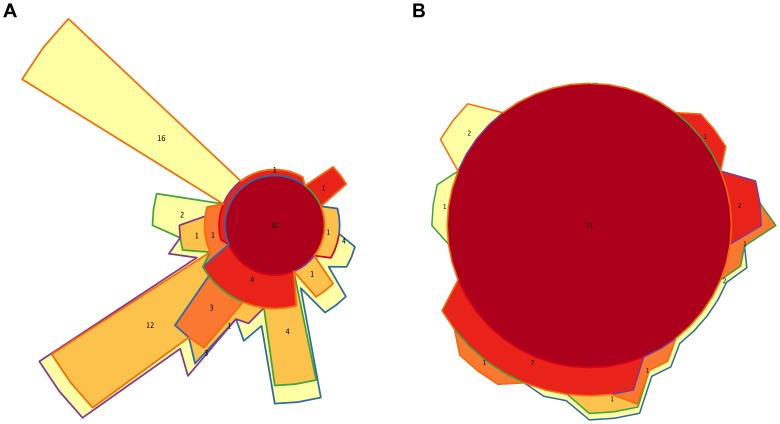
Timecore Venn diagrams. Changes in the core sets of genera (A) or functions (B) present at each infant timepoint. In both cases, areas representing the different timecores are enclosed by lines of the corresponding colors. The red central circles represent the genera or functions present in all five infant timecores; areas filled in dark orange, medium orange, light orange and yellow represent features present in four, three, two or one infant timecores. The number of features included in each section of the diagram is shown and areas are approximately proportional to these numbers.

We also identified separately the core genera of every timepoint (Table S9), and the Venn diagram in Figure 6A shows the intersections of the different infant “timecores”. New genera appear at every timecore, some of which remain in all subsequent timecores and are also present in those of the mothers. This is the case of *Bifidobacterium and Ruminococcus*, which join the core at timepoint I2, and of *Pseudoflavonifractor*, which joins at I3. At I4 there is an input of 12 new core taxa that will remain in the I5 timecore, including *Anaerostipes*, *Blautia*, *Coprococcus*, *Dorea*, *Fusobacterium* and *Roseburia*, and 16 new core genera make their appearance at I5, including *Acidaminococcus*, *Alistipes*, *Butyrivibrio*, *Parabacteroides* and *Subdoligranulum*. All of the core genera that are introduced in I4 and I5 are also present in the MB, and, with few exceptions, in the MA maternal timecores. In contrast, several genera of enteric bacteria appearing in the I2 timecore only remain through I3, or are maintained until I5 but are not present in the maternal timecores. Furthermore, all infant timecores except I1 include genera not present in any other infant timecore (in pale yellow in Figure 6A), pointing towards a continuous acquisition and loss of taxa throughout succession. Finally, *Desulfovibrio* and *Dialister*, as well as 17 rare genera, are present in both the MA and MB cores but not in those of any of the infant timepoints.

We also analyzed taxon dynamics by means of abundance plots of specific genera through time (data not shown) and with a Self-Organizing Map approach (SOM) that classified genera into groups with distinct abundance profiles along development. Figure S6A shows the three clusters of distinct temporal profiles (decreasing, increasing or peaking at I3) with >80% support in a bootstrapped SOM procedure. Only 18 genera, including *Klebsiella* and 10 other Proteobacteria, significantly grouped in the decreasing profile cluster, although the individual profiles of numerous other genera, such as *Bifidobacterium*, *Citrobacter*, *Clostridium*, *Enterococcus*, *Escherichia* and *Streptococcus*, also followed decreasing trendlines. A cluster including 11 genera whose abundances significantly peaked at I3 was also recovered. These genera were all rare, even at I3. Finally, the largest cluster grouped 31 genera that significantly increased after the I3 timepoint, mainly belonging to the Firmicutes.

At the functional level, Figure 3B shows that, for the TIGRFAM main functional roles, all samples have rather similar profiles, reflecting the fact that substantial functional requirements are likely shared among the different bacterial communities. Nevertheless, chi-square tests identify highly significant differences in the distribution of all main functional roles across timepoints (p≤0.001), except for “central intermediary metabolism” (p = 0.02) and “unclassified proteins” (p = 0.4). “Protein synthesis”, “transport and binding proteins” and “energy metabolism” predominate across all samples, with “protein synthesis” being the most abundant role in most cases and one or the other of the latter two roles being the most abundant in a small fraction of the infants' samples. Analyses at the TIGRFAM subrole level enable a better differentiation of the functional capacities present in the microbiota at different timepoints. Of the 116 subroles established in the TIGRFAMs database, 108 are detected in at least one of the samples, and 69 represent core functions detected in all. Two additional functions, “nitrogen metabolism” and “one-carbon metabolism”, are only absent in some maternal samples, elevating the number of core functions present in all infants to 71. In contrast, there are no functions that are absent from all infant timecores but present in all MA or MB samples. The Venn diagram in Figure 6B displays the intersections of the different infant timecores (Table S10), showing that very few functions beyond those of the common core are present in individual infant timecores or combinations thereof. The timecore of I1 is the most reduced, but is lacking only seven functional subroles that appear in the I2 timecore and remain thereafter. These subroles are those involved in the biosynthesis of polyamines, biotin and pyridoxine, in the transport and binding of nucleosides, purines and pyrimidines, in the tricarboxylic acid cycle of aerobic metabolism, and in cellular chemotaxis and motility, as well as one of the subroles related to mobile and extrachromosomal element functions. In addition, only 12 more functional subroles are present in one or a few of the infant timecores, including “cell envelope surface structures”, which is present in timecores I1 to I3, and “nitrogen fixation” and “DNA restriction/modification”, which only appear in the I5 and maternal timecores.

The SOM approach also identifies a few temporal trends in the abundance dynamics of TIGRFAM subroles, although with a bootstrap support lower than that obtained for the clustering of taxonomic profiles (Figure S6B). In particular, several subroles follow a sustained decrease from I1 to I5. These include several aerobiosis-related functions, such as the biosynthesis of lipoate and heme, essential cofactors of aerobic metabolism, and the Entner-Doudoroff pathway, an alternative to glycolysis used mostly by *Enterococcus, Escherichia* and other Proteobacteria during aerobic conditions. The decrease in this pathway is then concordant with the taxonomic trends described above. Other decreasing subroles are related to cell envelope surface structures and to pathogenesis, although toxin production and resistance functions fluctuate throughout the year without an increasing or decreasing trend.

### Potential patterns of association during community assembly based on presence/absence of taxa in diverse environments

**Figure 7:**
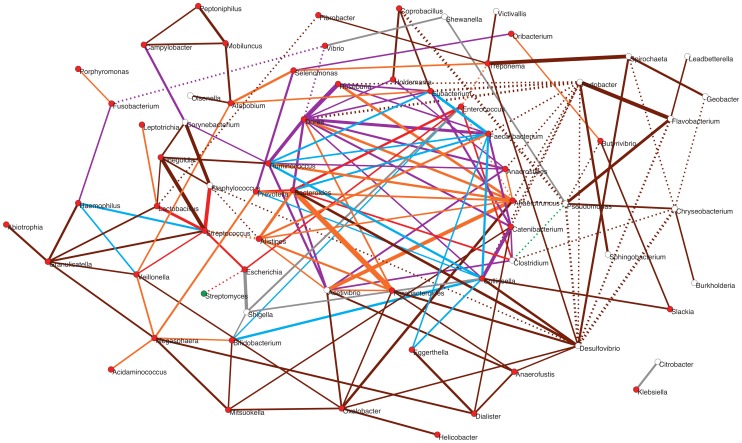
Potential taxon interactions during assembly of the gut microbiota. The represented subnetwork links all genera present in the different infant timecores and in the MB timecore, showing relationships inferred in a parent network based on presence/absence of taxa in multiple environments. We show with continuous lines those relations that have been identified as significant aggregations in the parent network, and with dotted lines the significant segregations. Relations are color-coded according to whether or not they are present in the maternal MB timecore and, for the relationships that are present at MB, according to the first timepoint in which they appeared. Relations that are not observed in the MB timecore are shown in grey; relations present only in MB are colored brown; relations appearing at I5 are colored orange; relations appearing at I4 are purple; relations appearing at I3 are green; relations appearing at I2 are blue; and relations appearing at I1 are red. Nodes are additionally colored according to their dominant environment in the original classification of Tamames *et al.* [56]. A dominant environment was assigned for a given genus when more than half of the samples where it was detected belonged to that environment. Red: host environments; Green: terrestrial environments; White: no particular preference for any environment (*i.e.*, cosmopolitan taxa). The thickness of the network edges represents the significance of the association (z-score).

To explore how positive and negative associations among taxa may have contributed to shape the gut's ecological succession, we investigated how the main genera detected in the infant and maternal gut microbiota relate within a network based on a wider environmental framework. We employed a previously constructed network based on presence/absence of taxa across a large variety of environments [56], the significance of which has been assessed by means of an appropriate null model (see Materials and Methods; Pascual-García A, Tamames J, Bastolla U, personal communication). For each infant and maternal timepoint, we extracted from this parent network the relationships of the timecore taxa. The subnetwork in Figure 7 represents the ensemble of these relationships for MB and all of the infant timepoints, color-coded according to whether or not they are present at MB and, for those that are, according to the first timepoint in which they appeared (see Figure Legend).

The overall topology of the subnetwork clearly delineates a central cluster populated by numerous links representing significant aggregations, surrounded by a much sparser peripheral “shell”. Remarkably, the central cluster exclusively contains taxa and relations that appeared from I1 to I5 and that are also present in the MB timecore (links colored in red, blue, purple or orange), while the outer shell is mainly formed by taxa and relations restricted to the MB timecore (links colored in brown). Network theory indicates that the existence of a central and densely connected set of nodes in a network facilitates system robustness and evolvability, helping adaptation to large fluctuations of the environment and to noise of intrinsic processes [69]. Regarding the temporal assembly of this central cluster, examination of the time of appearance of the different aggregations reveals that few of them existed at I1 (in red), although *Bacteroides*, *Clostridium* and *Enterococcus* formed a transitive aggregation already at this point. Transitive aggregations, where three or more taxa are linked to one another, are highly unlikely to occur by chance and their existence suggests that the involved taxa may sustain mutualistic relationships. In addition to this main triangle, a single other aggregation appears within the central cluster at I1, linking *Prevotella* to *Bacteroides*.

Following with the assembly of the central cluster, several new aggregations are formed at I2 (in blue) enabled by the appearance of *Ruminococcus*, *Faecalibacterium* and *Collinsella*, which are linked into a triangle. In addition, *Ruminococcus* and *Faecalibacterium* form another triangle with *Eubacterium* – which was already present at the I1 timecore without being linked to other genera. These two new triangles are linked to the *Bacteroides-Clostridium-Enterococcus* triangle through a single aggregation between *Faecalibacterium* and *Bacteroides*. Remarkably, in contrast to I2, no new aggregation is formed within the central cluster, or in the surrounding shell, at the I3 timepoint. Although this difference could be influenced by the fact that only 9 samples were available for the I2 timepoint, which could artefactually inflate the I2 timecore, the same result is obtained in a subnetwork based on timecores for the 9 infants who were sampled at all timepoints. This suggests that a stable stage of community assembly had been reached in the infants' gut by one month of age, at least with respect to the core taxa of the microbiota, which was not altered during the remaining months of exclusive milk feeding.

At I4, after the introduction of solid foods, a large number of novel aggregations (in purple) are again enabled by the appearance in the timecore of several Firmicutes genera. In particular, *Dorea* establishes a large number of links at this point, including numerous triangles and several larger cliques (subgraphs in which all nodes are connected to each other) that link different Firmicutes genera, as well as a triangle formed by *Dorea*, *Faecalibacterium* and *Bacteroides*. At I5, another Clostridiales genus, *Anaerotruncus*, and two Bacteroidales, *Parabacteroides* and *Alistipes*, join the central cluster forming numerous aggregations. *Anaerotruncus* links with the Bacteroidales genera *Alistipes* and *Prevotella*, and with nearly all of the Firmicutes genera that appeared at I4, forming numerous triangles and one clique. On their part, *Parabacteroides* and *Alistipes* are also involved in several links and transitive aggregations, including a clique with *Acetivibrio* and *Bacteroides*.

It is worth noting the abundance of transitive relations that are enabled in the central cluster at I4 and I5, consolidating its structure and indicating that the introduction of solid foods to the infants' diet likely promoted an increase in the complexity of community assembly. Moreover, as already mentioned, the genera restricted to the MB timecore do not join the central cluster of the subnetwork, and rather form a surrounding “shell” that is connected with this cluster through a moderate number of aggregations. This suggests that, although community assembly was still not complete by the one-year mark, the main nucleus of the gut community was already established at this point. Interestingly, network theory indicates that core/periphery structures may form at times of environmental stress, leading to the development of more condensed network structures and the segregation of a network core [69]. In the infant gut, the introduction of solid foods between I3 and I4 represented a major disturbance that must have permanently altered the conditions of the gut environment, and the resulting stress in the subsequent months may have promoted the consolidation of the central cluster.

Besides those that form the central cluster of the subnetwork, several genera appear during infant development that only connect to this cluster through a limited number of direct links or longer paths. In fact, most of the genera present at the I1 timecore are located outside of the central cluster. Remarkably, these include a small (red) star-shaped subgraph with the global-core genus *Streptococcus* at its center, which aggregates *Escherichia* and *Lactobacillus*, also members of the global core, and *Staphylococcus* and *Veillonella*, which appear at I1 but are not maintained in all infant timecores. This subgraph is only connected to the central cluster through a (red) link between *Escherichia* and *Enterococcus*. Several other peripheral genera directly join this subgraph at other timepoints. These additional genera enable a few more aggregative links with the central cluster, as well as several paths linking the subgraph to other peripheral genera, mostly appearing at I5 or MB. On the other hand, the subgraph genera are also involved in several segregative relations appearing at different timepoints, with *Streptomyces*, *Desulfovibrio*, *Fibrobacter* and the central cluster genus *Alistipes*.

Other peripheral genera appearing early on during infant development include *Bifidobacterium*, which is only connected to the central cluster through links to *Collinsella* and *Enterococcus* (in blue), and a series of Proteobacteria. Among these, we find the global core genus *Vibrio*, which never connects to the central cluster, and directly segregates from it through the genus *Dorea*. Several other peripheral genera appear at the I4 and I5 timecores, connecting to the central cluster through direct links (in purple or orange) or aggregative paths. Interestingly, these include the spirochaete *Treponema*, which is considered atypical in urban populations and had until now mostly been detected in rural populations of Africa and South America and in ancient mesoamerican remains [25], [70]. In addition, two enteric Proteobacteria, *Citrobacter* and *Klebsiella*, are present as a separate component of the subnetwork, linked to each other but involved in no other relationship. *Citrobacter* and *Klebsiella* are restricted to the I2–I5 and I2–I3 timecores (Table S9), respectively, although they can also reach high abundances in individual infants at other timepoints (Figure S2). This scenario suggests that these taxa, along with *Shigella* and *Shewanella* that only momentarily join the subnetwork, are not permanent residents of the microbiota, but perhaps opportunists that take advantage of transient conditions in the infant gut.

Regarding the genera that join the periphery of the subnetwork as part of the MB timecore, *Desulfovibrio* and *Oxalobacter* are the ones showing the larger number of aggregations to the central cluster. The aggregations detected for *Desulfovibrio* are of note, since they reflect experimentally established cross-feeding relationships of this H_2_-consuming, sulfate-reducing bacterium with the H_2_-producer *Collinsella* and the sulfatase-encoding Bacteroidales genera [71]. On the other hand, there is on the right side of the subnetwork a series of MB genera that clearly segregate from the central cluster, mainly through direct segregative links sustained by *Pseudomonas* and *Pedobacter*. Interestingly, most of these genera have been classified as cosmopolitan rather than mainly host-associated [56], and none of them ever reaches abundances of 1% in any sample. In addition, approximately half of these genera are absent from the MA timecore (Table S9), suggesting that they can be easily displaced when there are alterations of the gut environment. This scenario suggests that these late-appearing taxa might be facultative members of the gut microbiota or allochthonous species that frequently make their way to the gut without establishing as main components of the community.

### Putting it together: Overall patterns of microbiota development delineate a successional process redirected by the introduction of solid foods

The various patterns of microbiota development described in the preceding sections suggest that during the time-course analyzed we are likely observing two major, distinct colonization phases, separated by the introduction of solid foods to the infants' diets. The first colonization phase would encompass the period during which infants were fed only milk, *i. e.*, timepoints I1 to I3. During this period, the richness, diversity and complexity of interactions among taxa tend to increase in I1–I2, indicating that the relatively simple bacterial communities present by one week can tolerate the arrival and establishment of new species, to which the infants would undoubtedly be exposed during their first weeks of life. The variable behavior of taxon richness and diversity observed across infants during the I2–I3 period suggests that, by three months of age, different infants were at different stages of community development, with some still incorporating new species while others were starting to loose species, most likely due to interspecific competition. In the infants that underwent decreases in richness during this period, changes in Shannon values were not concomitant (Table S6), supporting the notion that interspecific competition purged the community of rare taxa, presumably not well adapted to thrive during this milk-feeding period. Accordingly, *Bifidobacterium* or *Bacteroides*, the only genera capable of thriving on both lactose and HMOs [59], dominated the I3 microbiota in nearly all infants, with the exception of those born by C-section (Figure S2), which supports the notion that C-section delays the establishment of these genera [51]. Moreover, among vaginally delivered infants, *Bacteroides* or *Bifidobacterium* dominated when mothers did or did not receive antibiotics during delivery, respectively. The I3 CCA (Figure S3) confirms that delivery type and use of peripartum antibiotics explain 22% and 12% of the taxonomic composition variation of the infant microbiota at this timepoint (Table S5).

Classical models of succession posit that, after a period of competition leading to species loss, community stability will eventually increase in late successional stages, after which major community shifts will not occur unless a significant disturbance affects the ecosystem [52]. In our data, no such stabilization is observed, as the variable I2–I3 period is followed by a strong decrease in richness in I3–I4, followed by a trend towards richness recovery in I4–I5 (Table S6, Figure 1A, 1C). As stated earlier, these richness changes are mainly due to the loss and gain of rare genera, and are accompanied by opposite trends at the level of diversity (Table S6, Figure 1B, 1D). In addition, the number of core microbiota genera shared by all individuals increases importantly at I4 and I5 (Table S9, Figure 6A), with substantial repercussions on the configuration of relationships among taxa (Figure 7). Most likely, the introduction of solid foods between I3 and I4 contributed importantly to prevent the stabilization of the community, as this chronic disturbance altered the resources present in the gut environment. With solid foods, the variety of nutrients that become available to the infant gut microbiota clearly expands, potentially providing a larger number of niches for different organisms and contributing to the increase in diversity observed at I4. In particular, carbohydrates will now be available in a larger variety of forms, including numerous complex molecules found in cereals, fruits, vegetables and tubers (Table S1), providing a selective challenge for the milk adapted resident community.

Our observation that solid food introduction is followed by a purge in rare taxa is consistent with the idea that fewer species will persist in the face of intense disturbances [72]. In the 7-months infant, the genera that thrived in the milk-adapted microbiota - *i. e.*, *Bifidobacterium* and *Bacteroides* - continue to dominate, with the latter genus being now the most abundant in a majority of individuals. The rise of *Bacteroides* following the introduction of solid foods has been observed in previous studies [34] and is likely due to its large versatility for complex carbohydrate degradation. Nevertheless, some genera that had not been previously detected at high abundances (or only in very few individuals) expand now in the gut microbiota, in agreement with the notion that disturbance should facilitate invasion of the community by new species [73]. This is the case of *Ruminococcus*, which is now found among the most frequent genera in nearly all infants. *Ruminococcus* thrives on oligosaccharides such as raffinose and sucrose that constitute the most abundant soluble saccharides in plant tissues and is capable of partially degrading insoluble plant fibers such as lignin and cellulose [74], which likely explains its competitive advantage after the introduction of cereals, fruits and vegetables into the diet. Another genus that reaches high abundances for the first time in some 7-months-olds is *Akkermansia*, one of the main mucin-degraders in the gut microbiota [60], [75]. Mucin production is dependent on the availability of dietary amino acids and should increase with the higher protein content of solid foods, enabling the growth of mucin-specialized bacteria. On the other hand, the disturbance created by solid foods does not seem to enable invasion of the gut community by opportunistic species, as fast growers such as *Escherichia* rather decrease in abundance from I3 to I4. Moreover, the “pathogenesis” functional subrole also decreases markedly after the I3 timepoint, indicating that opportunistic pathogens are not taking advantage of the disturbance.

In the last time interval analyzed, I4–I5, taxon richness tends to increase again mainly due to the acquisition of new rare taxa. This indicates that succession has now entered a second period of net species recruitment, although most incoming taxa have not been able to reach substantial frequencies, suggesting that the pre-established populations retain a competitive advantage. Nevertheless, substantial shifts occur during this period in relative taxon abundances. Several of the most abundant genera at I4 - *i. e.*, *Bifidobacterium*, *Veillonella*, *Escherichia* - decrease substantially in I5. At the same time, the main butyrate producers of the gut microbiota, *i. e.*, *Faecalibacterium*, *Eubacterium* and *Roseburia*, rise in abundance at this timepoint, with *Faecalibacterium* becoming the second most abundant genus overall (after *Bacteroides*). In addition, other SCFA producers, such as *Blautia* and *Butyrivibrio*, reach frequencies above 1% for the first time in I5. Between I4 and I5, the diet of Spanish Mediterranean infants changes substantially, as it becomes progressively similar to that of adults [36]. During this period the general consumption of animal protein increases importantly, as meats, fish, eggs and dairy products become more prevalent (Table S1). At the same time, the contribution of cereals continues to increase, probably enabling the rise of genera adept at fermenting starches and fiber, such as *Bacteroides* and the butyrate producers *Faecalibacterium*, *Eubacterium* and *Roseburia*.

As a result of these changes, the ranking of taxon abundances observed in the one-year-old infants becomes remarkably similar to that of the mothers, with *Bacteroides*, *Faecalibacterium*, *Clostridium* and *Ruminococcus* present among the five top genera in I5, MA and MB (Figure S2). However, differences exist in the relative abundances of *Bifidobacterium* and *Eubacterium* between mothers and one-year-olds, with the first genus remaining more common in I5 while the latter has not yet reached the high levels at which it is found in MA and MB. Moreover, the richness (Figure 1C), diversity (Figure 1D) and complexity of interactions among taxa (Figure 7) at the one-year mark are still far from those observed in the maternal samples. Similarly, pairwise ANOSIM analyses (Table S7) and ordination techniques (Figure 4) detect differences in taxonomic and functional composition between I5 and the maternal samples, further corroborating that succession was incomplete at the one-year mark. In agreement, recent cross-sectional studies have suggested that an adult-like gut community may not be reached before three years of age [25].

In conclusion, our analyses of GIT microbiota development during the first year of life reveal an incomplete successional process, strongly marked by the introduction of solid foods to the infants' diets. Therefore, important questions regarding microbial succession in the infant GIT still remain for further analysis. A longer sampling period would be necessary to reveal the final progression of the gut microbiota towards an adult-like stage, and a tighter sampling around the time of introduction of solid foods would be required to clarify the transition that accompanies this event. On the other hand, in order to gain an in depth understanding of the ecological and evolutionary processes at play in this environment, we will need to focus on the genetic structure and demographic dynamics of microbial populations as they settle within the gut.

## Materials and Methods

### Ethics statement

This study was approved by the Ethics Committee of the Center for Public Health Research (CSISP), Valencia, Spain. All women participating in the study read and signed forms of informed consent specifically approved for this project by the Ethics Committee.

### Sample collection, pyrosequencing and initial processing of sequencing reads

Fecal samples were collected by the mothers and stored in home freezers until brought to the laboratory, where they were stored at −80°C until processing. Samples were homogenized in a 50% RNA later/phosphate saline buffer solution and centrifuged for two minutes at 2000 rpm. Only the supernatant resulting from the latter spin was used for further processing. DNA was extracted using the Epicenter Master Pure Complete DNA & RNA Purification kit following manufacturer's specifications, except for an additional digestion step at the beginning of the extraction protocol with lysozyme for 30 minutes at 37°. Samples were then prepared for 454 pyrosequencing by adding a barcode and pooling them in groups of 20 samples per run, which provided between 35000 and 70000 reads per sample. Only reads that passed quality controls (average base score quality per read >20) were further analyzed after elimination of read replicates by means of CD-HIT-454 [76]. We addressed downstream analysis at read level rather than at contig level based on the prior assessing of the complexity of our communities, as simulation studies have determined that chimeras are particularly prevalent among contigs lower than 10 kbp in size [77], [78]. High-complexity microbial communities lacking dominant populations rarely produce contigs larger than 10 kbp, prompting the recommendation that such data sets should not be assembled at all.

### Gene calling, taxonomic assignment and functional annotation

We used a combination of evidence-based and *ab initio* gene calling. In the first step, coding regions were identified based on homology searches at read level via BLASTX [79] against the NCBI-nr protein database considering an e-value cutoff of 0.001. Subsequently, we used GLIMMER3 [80] to identify any coding regions that were missed in the previous step by means of a fine-tuned IMM (Interpolated Markov Model). We used the ‘-X’ GLIMMER3 option, allowing fragmented ORF (Open Reading Frame) identification, and default settings for other options. In order to build the IMM we chose eight complete bacterial genomes from NCBI spanning the main gut microbiome phyla (Firmicutes, Bacteroidetes, Actinobacteria and Proteobacteria) and then extracted the reported ORFs to train the model.

Taxonomic classification was only performed on coding regions found by BLASTX, by using Blast2.lca (https://github.com/emepyc/Blast2lca#readme). This methodology is based on a Last Common Ancestor (LCA) algorithm, which retrieves the most specific taxon associated with the complete set of sequences that hit a certain query, instead of only considering the taxon associated with the closest BLASTX hit, thereby reducing false matches. Eukaryota-related coding regions were filtered out from the analysis based on superkingdom LCA annotation, or on BLASTN searches (0.001 e-value cutoff) against the NCBI-nt eukaryotic subset in the case of those regions identified by the *ab initio* approach. Finally, to functionally annotate the identified coding regions we used HMMER2 [81] against the TIGRFAMs (9.0 release) database of prokaryotic models [82], considering an e-value cutoff of 0.1. HMMER is a protein profile aligner based on hidden Markov models, with high sensitivity for classifying remote homologs [83].

### Microbiota richness and diversity

We assessed the taxonomic and functional richness and diversity of the microbiota by means of several estimators. In order to eliminate possible artifacts introduced by read count differences between samples, we first used QIIME [84] for resampling an equal number of reads per sample. The richness estimators N and Chao1 [63] and the Shannon diversity index [64] were then calculated using the library ‘vegan’ from the R package [85]. The Chao1 estimator was chosen because it has been shown to be one of the most reliable non-parametric estimators of species richness in species-rich samples [86]. The Shannon index was preferred for species diversity because of its use of natural logarithms of relative species abundances, which reduces the weight of the more abundant species and renders it sensitive to the changes in rare species, which are common in infant gut microbiota samples.

Linear regression analyses were executed to determine the statistical significance of the changes in richness and diversity through time, both over all timepoints and in pairwise comparisons for specific time intervals. Because taxon richness is assumed to follow a Poisson distribution, we employed the ‘glm’ function implemented in the ‘stats’ R package to fit generalized linear models. On the other hand, values of the Shannon diversity index were parameterized in the standard unit interval (0, 1) and assumed to follow a Beta distribution; therefore we applied the Beta regression model, as implemented in the ‘betareg’ [87] R package. Hierarchical clustering analysis of the temporal profiles of richness and diversity change for individual infants was performed in the ‘pvclust’ R package, which assesses clustering uncertainty by means of multiscale bootstrap resampling [88].

### Microbiota composition clustering, directionality and dynamics

The R package was employed for comparative analyses of taxonomic and functional microbiota composition. Heatmaps and clustering analyses were based on the Bray-Curtis distance as a measure of dissimilarity [65]. Directionality in taxonomic and functional composition change through time was assessed by means of various multivariate analyses. First, we employed global and pairwise analyses of similarities (ANOSIM) adjusted for multiple testing to detect whether there were significant differences between taxonomic or functional profiles per timepoint. ANOSIM tests whether there is a significant difference between two or more groups of samples by comparing distances between sample groups to those within groups. In addition, we also performed “Permutational Multivariate Analysis of Variance Using Distance Matrices” (PMANOVA or ADONIS), which yielded similar results to the ANOSIM (data not shown). Both analyses used the Bray-Curtis distance to measure dissimilarity in taxonomic or functional microbiota composition between samples. To explore further the pattern of similarities among timepoints we performed Canonical Correspondence Analysis (CCA) and Principal Coordinates Analysis (PCoA) using Gower distances [67], for both taxonomic and functional data sets. Once the PCoA analyses were executed, we drew convex hulls enclosing all samples pertaining to a particular timepoint and calculated the area of overlap of the polygons representing each timepoint.

The dynamics of individual genera and functions through time were also examined within R. The behavior of different genera was analyzed by means of regression analyses using the Poisson model and the ‘GeneFamilies.regression’ function from the ‘ShotgunFunctionalizeR’ library, and also through the drawing of Venn diagrams containing the taxa per individual, MIP or timepoint, using the ‘venn’ function in the ‘gplots’ library. Venn diagrams were also constructed to identify taxonomic and functional “timecores” containing the taxa or functions shared across all individuals at a given timepoint using the ‘compute.Venn’ function in the ‘Venerable’ library, and to identify those features restricted to single timecores or combinations thereof. Self-Organizing Maps (SOM) [89] were constructed for both taxonomic and functional data sets, using the function ‘som’ from the ‘som’ library. These maps are artificial neural networks that use a neighborhood function to separate a complex, high-dimensional input space into a reduced number of discrete groups with unique behaviors through time. In order to get reliable SOM-based clusters we used the bootstrap method. Firstly, we built 200 different sets of resampled temporal profiles for each feature (genus or function) by resampling entire profiles of randomly selected individuals. Then, we carried out a SOM-based clustering over this 200-fold-sized data set. To build clusters at different support levels, we retrieved only those features whose profiles were classified into the same cluster in at least 60% or 80% of the resampling sets.

### Constructing a dendrogram of genus-level functional profiles

Functional profiles were determined for those genera present in any sample at >1% abundance in addition to having genes representing at least 50% of the 108 TIGRFAM functional subroles detected in our complete dataset. Because not enough information was recovered in a sample per sample basis for each genus, the functional profile was established by pooling all the samples of a timepoint. Functional profiles were defined as vectors containing the relative abundances of each one of the 108 TIGRFAM subroles in a particular genus and timepoint. Bray-Curtis distances between functional profiles were computed using the ‘bcdist’ function from the R ‘ecodist’ library, and dendrograms based on these distances were drawn using the ‘hclust’ function from the R ‘stats’ library with the complete-linkage method.

### Extracting gut microbiota taxa co-occurrence networks from a parent network based on diverse environments

We analyzed the relations of the main gut microbiota genera detected in our study within a parent network previously constructed based on the presence/abscence of taxa across a large variety of environments (Pascual-García A, Tamames J, Bastolla U., personal communication). For each infant and maternal timepoint, we considered the group of *N* taxa observed in all samples of the timepoint (the timecore). For each group we had then *N(N-1)/2* putative interactions and we determined those that were present in the parent network, which includes all significant associations among 1187 different genera observed in 2322 samples from very different environments. Details about the environments and their classification can be found in [56]. The parent network was obtained from an adaptation of the null model proposed by Navarro-Alberto and Manly [90] where environmental preferences are considered in order to avoid trivial associations. The null model allows for the generation of random realizations of the original data assuming that taxa are not associated. The significance of putative associations can then be assessed by comparing the results obtained from the observed data *versus* those obtained from the random ensemble. As the random realizations do not contain information about real associations, any signal coming from the random ensemble is considered a false positive, serving to establish a restrictive threshold for the estimated false positive rate.

## Supporting Information

Figure S1 Heatmaps and clustering of MA and MB maternal samples according to taxonomic composition (A) and main TIGRFAM functional roles (B) based on Bray-Curtis distances. (A) Only the genera above 1% abundance in at least one sample are depicted. Each sample is identified at the bottom of the heatmaps by a code that specifies the MIP (Mother Infant Pair) to which it belongs and the corresponding timepoint.
(PDF)

Figure S2 Heatmaps and clustering of the samples for each timepoint according to taxonomic composition (A) and TIGRFAM main functional roles (B) (details as in Figure S1).
(PDF)

Figure S3 Canonical Correspondence Analyses (CCA) showing the effect of C-section on the taxonomic composition of the microbiota at different timepoints. The proportion of variability explained by C-section delivery is highest at I1 (16%), I2 (22%) and I3 (22%) and decreases at I4 (10%) and I5 (10%), and is always below the proportion of variability explained by the first unconstrained axis.
(PDF)

Figure S4 Linear regressions of richness (Chao1 estimator) and diversity (Shannon index) *vs.* time (A–B taxonomy, C–D function).
(PDF)

Figure S5 Heatmaps and clustering of the samples for each MIP according to taxonomic composition (A) and TIGRFAM main functional roles (B) (details as in Figure S1).
(PDF)

Figure S6 Self-Organizing Maps (SOM) of taxon and function dynamics. SOMs identify patterns of abundance dynamics in the infants throughout development at both taxonomic (A) and functional (B) levels. The number of genera (A) or functions (B) included in each represented cluster is indicated (cluster size). Clusters have 80% and 60% bootstrap support for taxa and functions, respectively. For each cluster, average values on each timepoint along with their corresponding 95% confidence intervals are shown, in a scale centered at the mean of all samples and scaled by the standard deviation.
(PDF)

Table S1 Information on consumption of different foods, obtained from questionnaires answered by the infants' parents.
(DOCX)

Table S2 Details of pyrosequencing reads and annotation per individual sample.
(DOCX)

Table S3 Taxon abundances per sample. Numbers correspond to raw sequence counts.
(TXT)

Table S4 Function abundances per sample. Numbers correspond to raw sequence counts.
(TXT)

Table S5 Variability explained by constrained (CCA1) and unconstrained (CA1 and CA2) axes in Canonical Correspondence Analyses when the constraining variable is delivery type, use of peripartum antibiotic or exclusivity of breastfeeding.
(DOCX)

Table S6 Richness (N and Chao1 estimator) and diversity (Shannon index) for taxonomic and functional data in individual samples.
(DOCX)

Table S7 p-values of ANOSIM pairwise comparisons between timepoints. Statistically significant values (p<0.05) are shown in red.
(DOCX)

Table S8 Taxonomic and functional dissimilarities between timepoints estimated as the non-overlapping areas of the convex hulls representing them in the PCoAs of Figure 4C–D. Dissimilarity values above 0.80 are shown in red.
(DOCX)

Table S9 Taxonomic timecores. Timecores are defined as lists of genera present in all individuals at a given timepoint. All genera present in at least one timecore are listed and their presence (1) or absence (0) at each timecore is reported.
(TXT)

Table S10 Functional timecores. Timecores are defined as lists of functions present in all individuals at a given timepoint. All functions present in at least one timecore are listed and their presence (1) or absence (0) at each timecore is reported.
(TXT)
