# Supplementary figures and images for: Microbial Succession in the Gut: Directional Trends of Taxonomic and Functional Change in a Birth Cohort of Spanish Infants
Source: PLoS Genet. 2014 Jun 5;10(6):e1004406. doi: 10.1371/journal.pgen.1004406 (PMC4046925; doi:10.1371/journal.pgen.1004406)

# Mothers

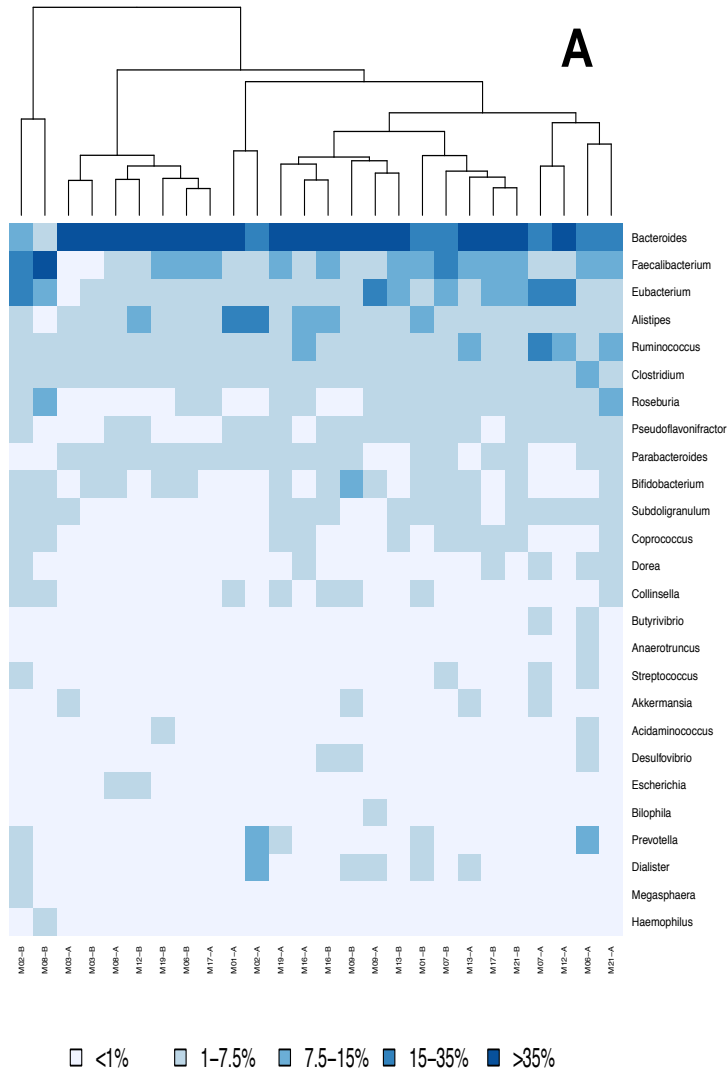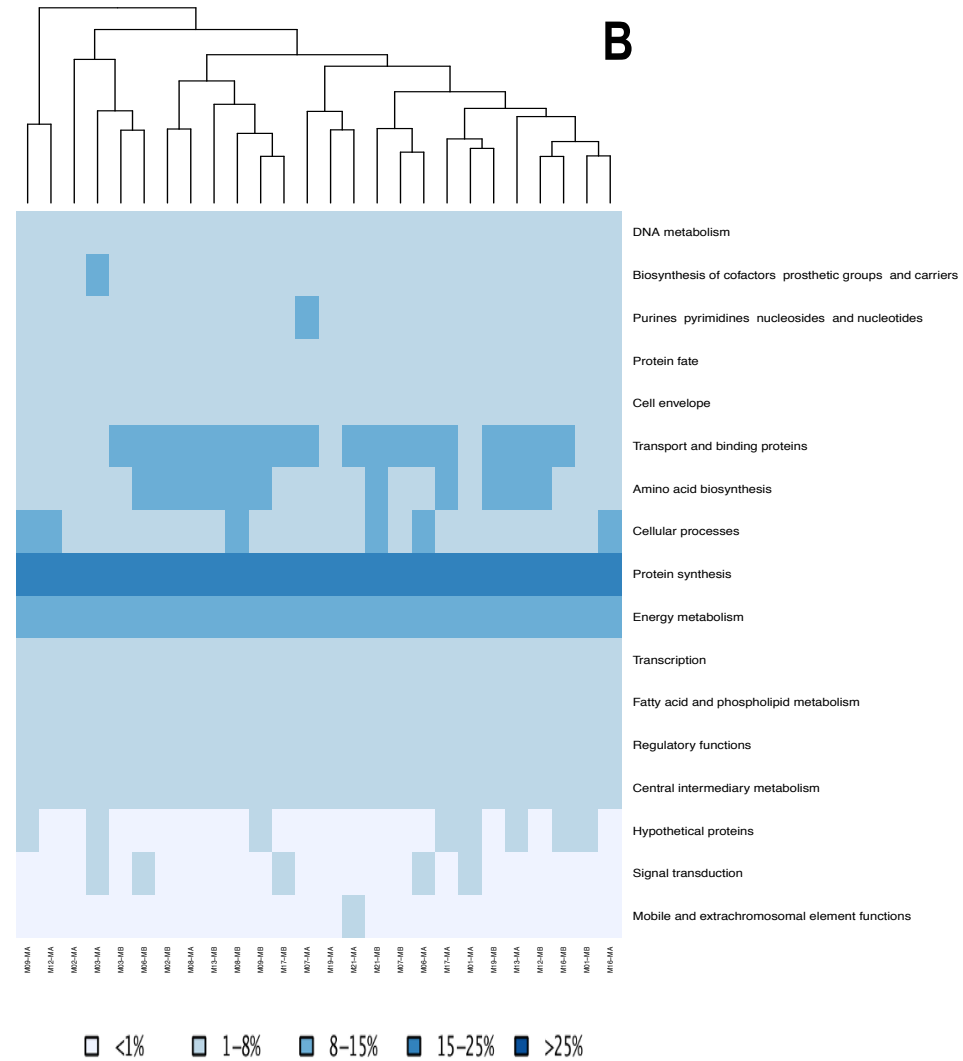

Supplement: Figure S1 — Heatmaps and clustering of MA and MB maternal samples according to taxonomic composition (A) and main TIGRFAM functional roles (B) based on Bray-Curtis distances. (A) Only the genera above 1% abundance in at least one sample are depicted. Each sample is identified at the bottom of the heatmaps by a code that specifies the MIP (Mother Infant Pair) to which it belongs and the corresponding timepoint. (PDF) [file pgen.1004406.s001.pdf]

# TP-I1

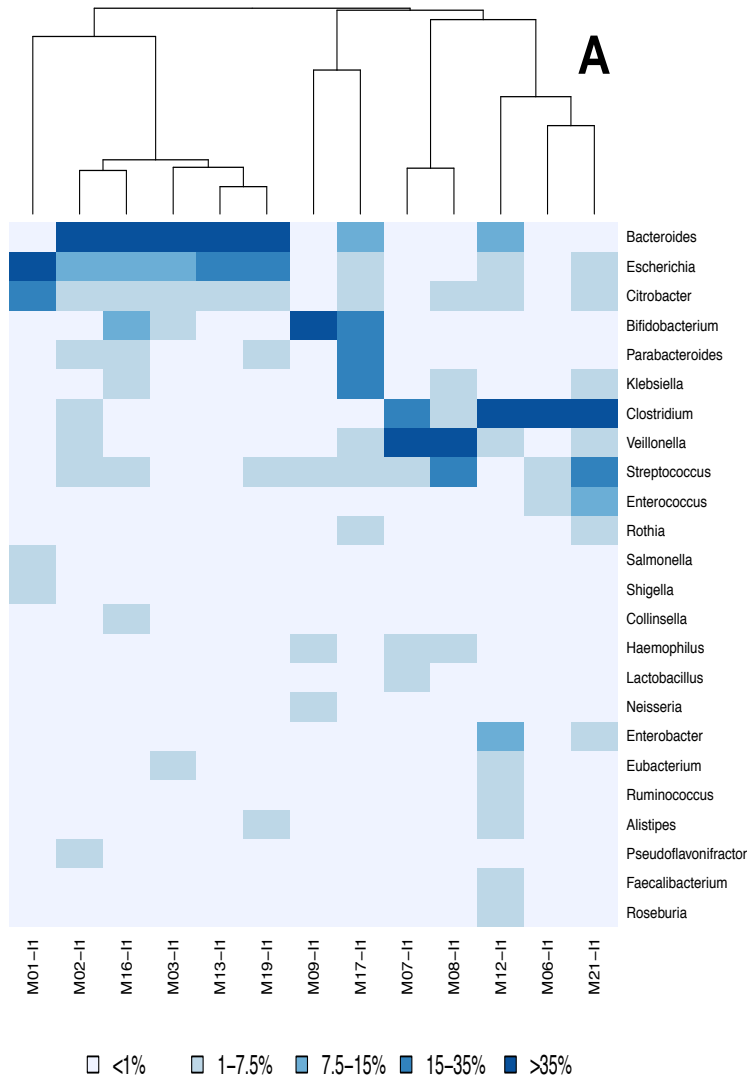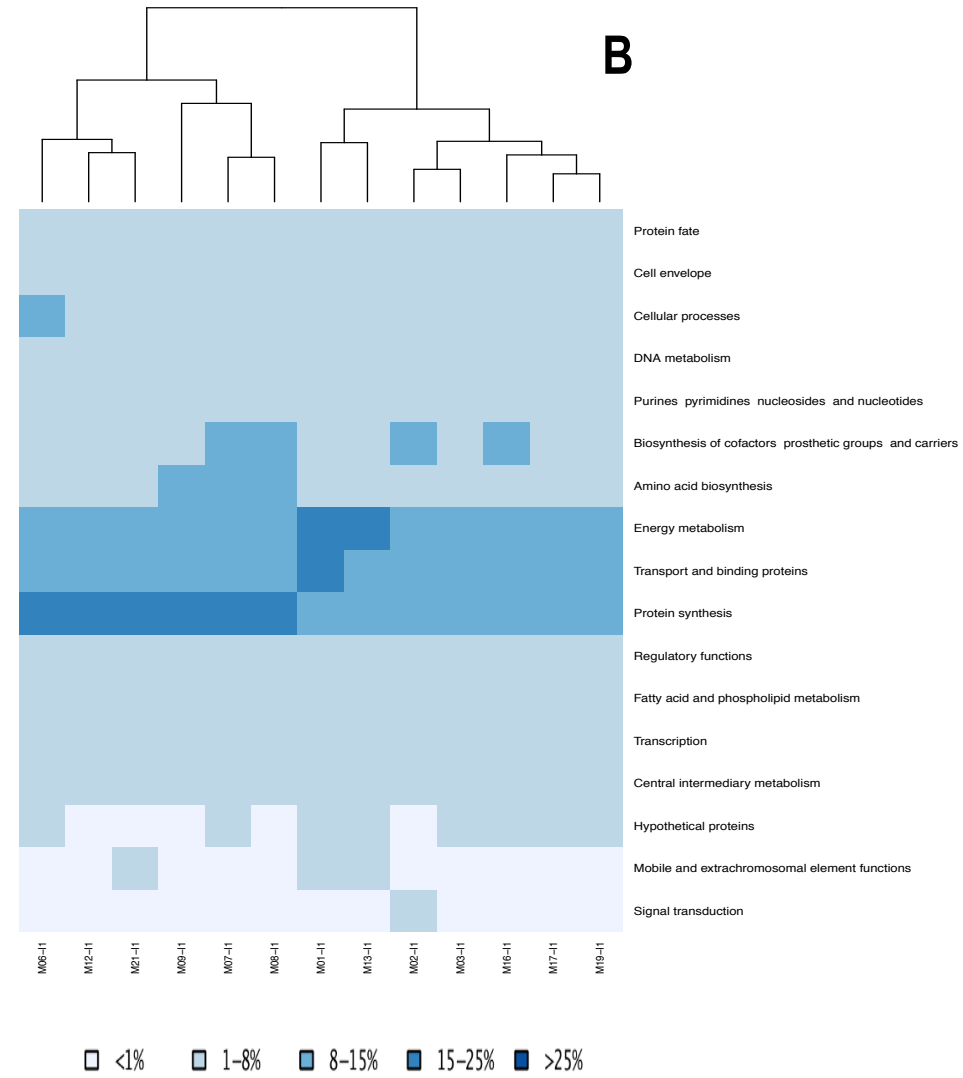

# TP-12

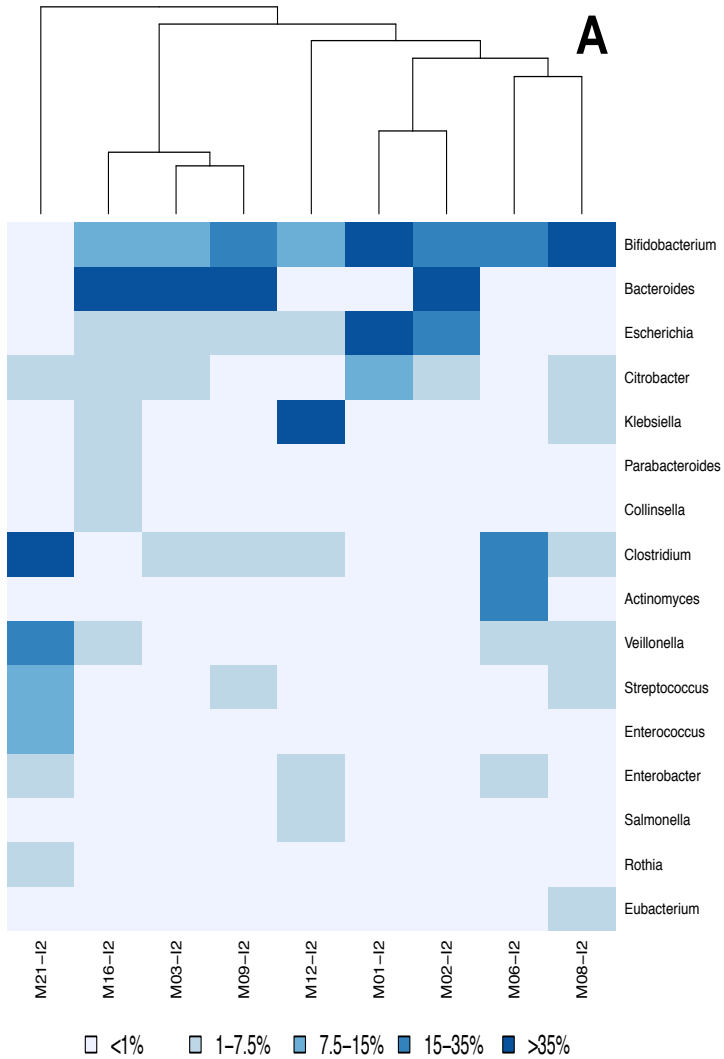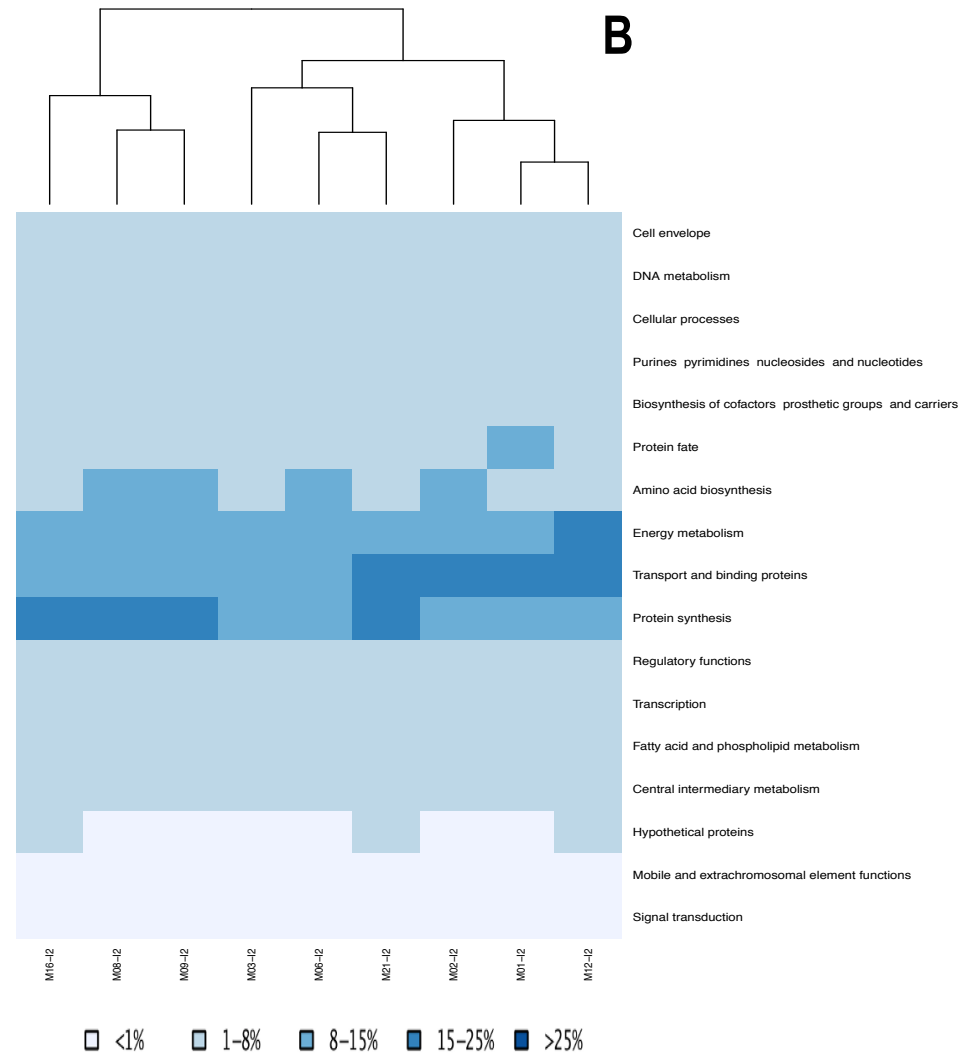

# TP-13

**A**

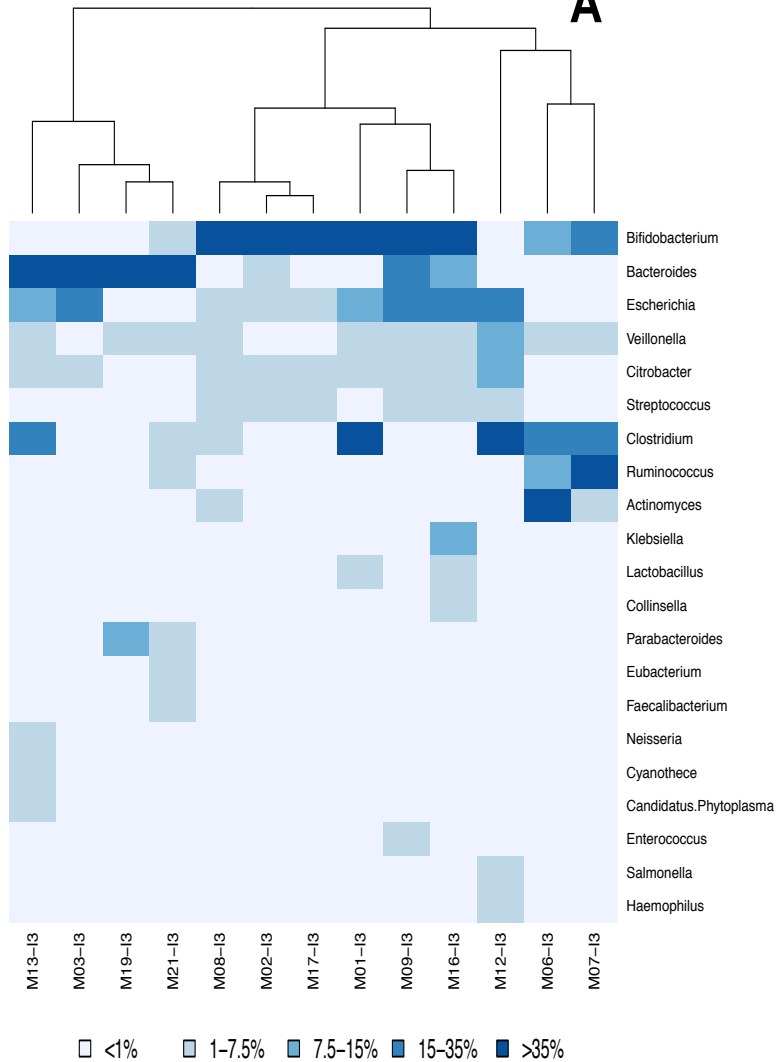

**B**

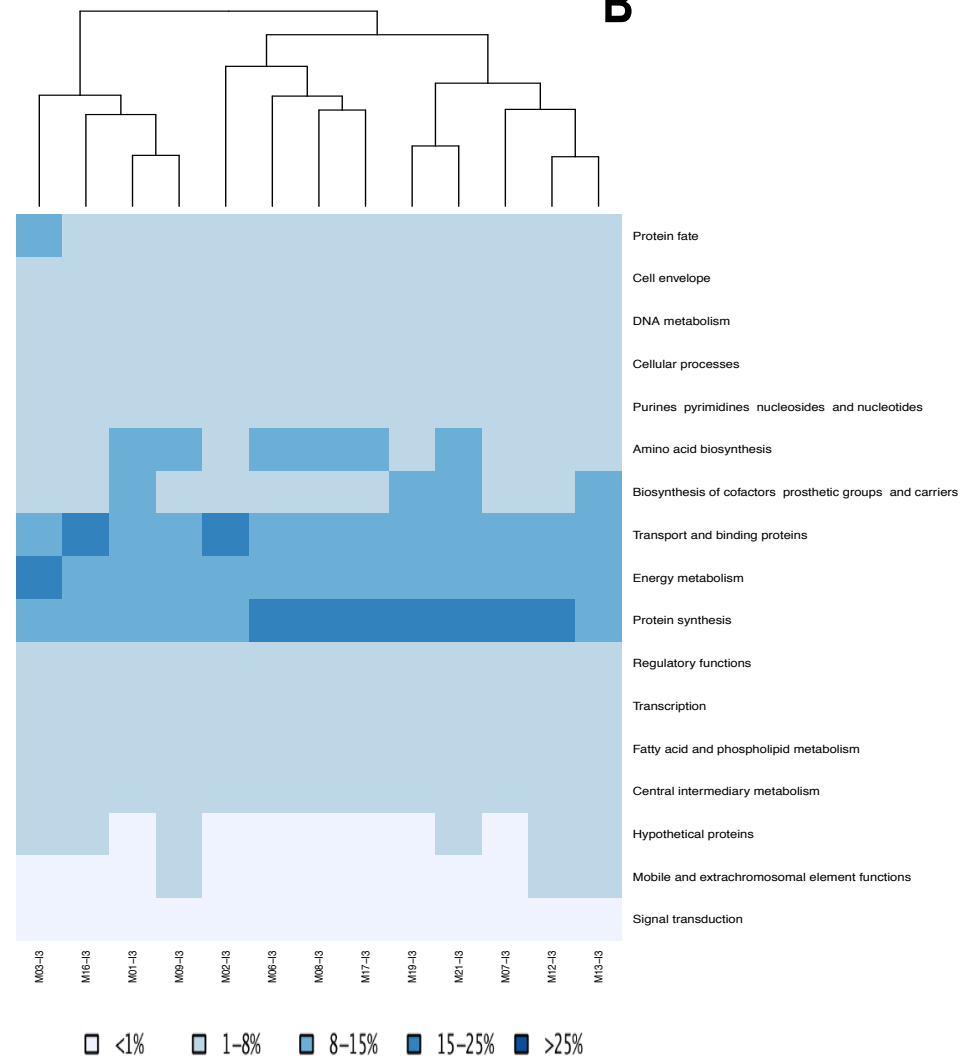

# TP-14

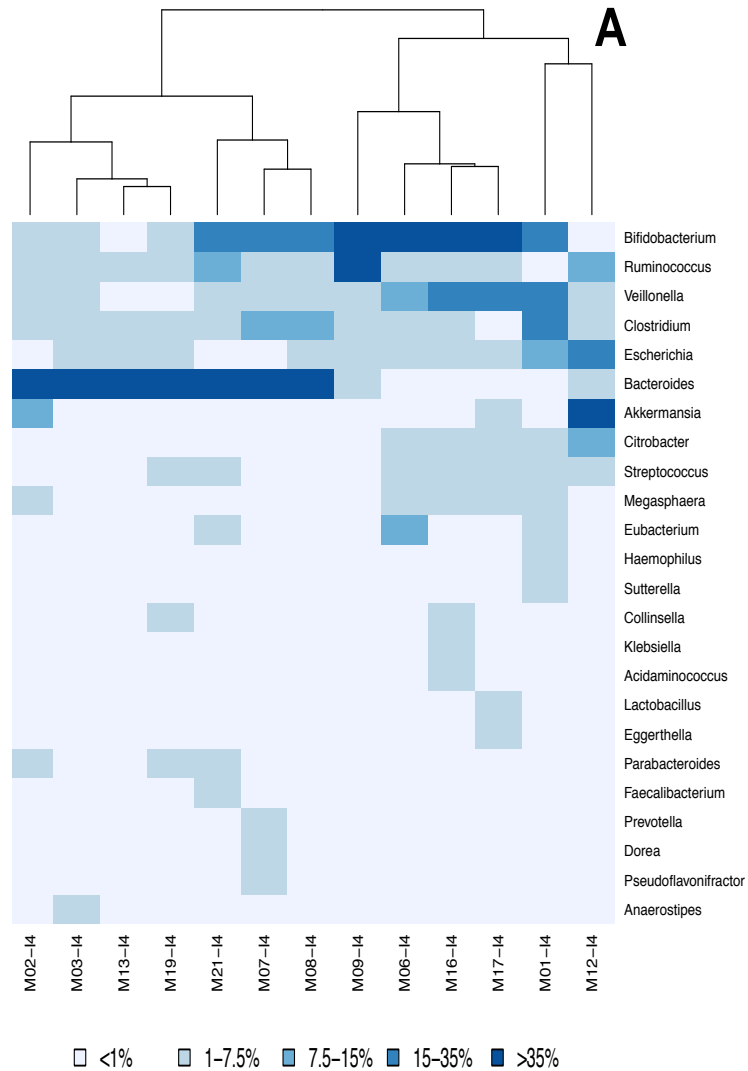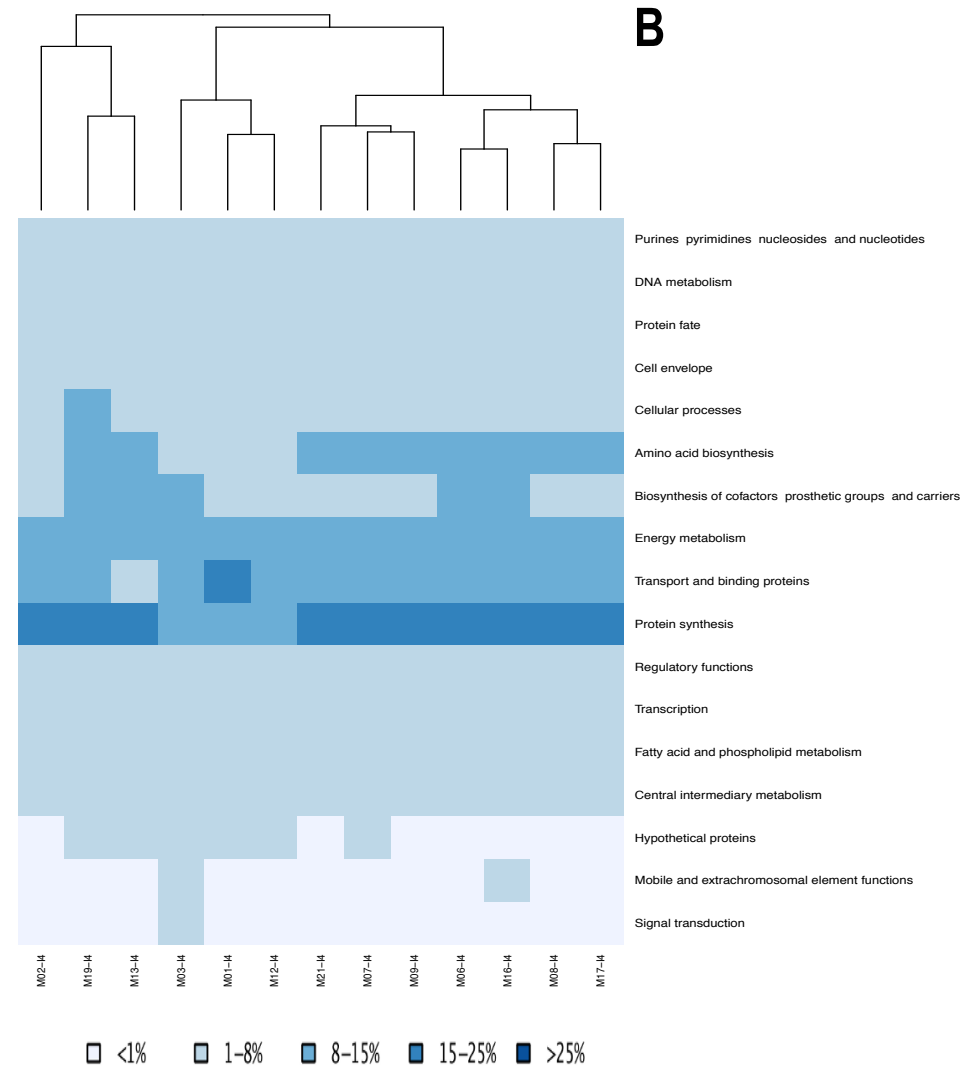

# TP-I5

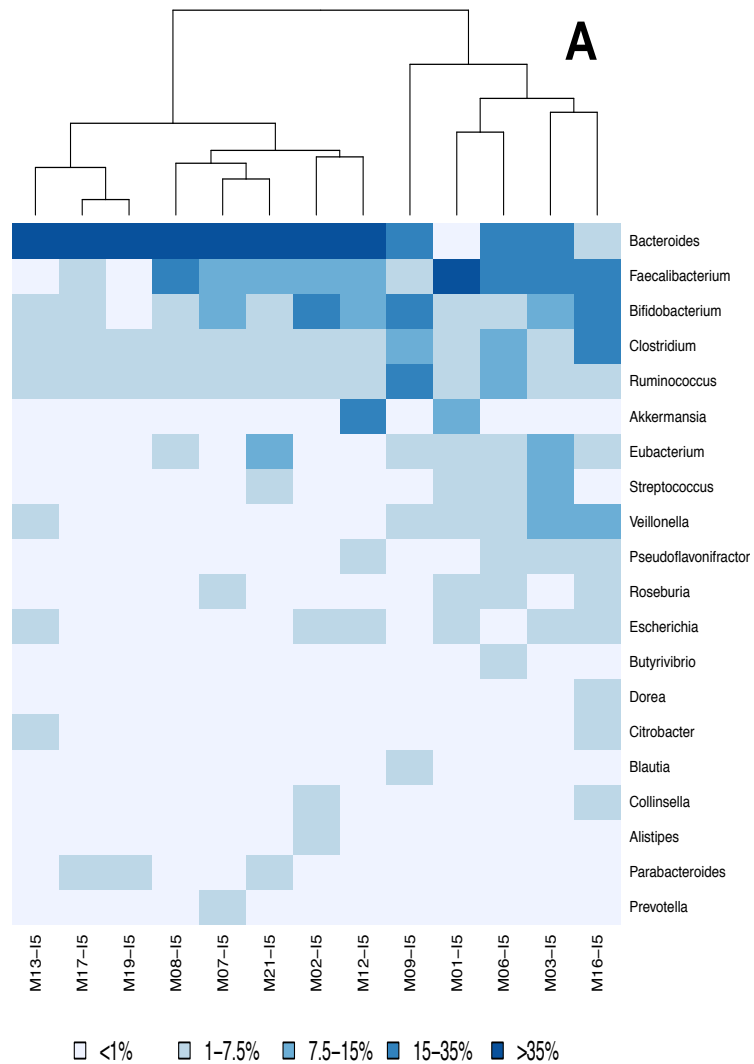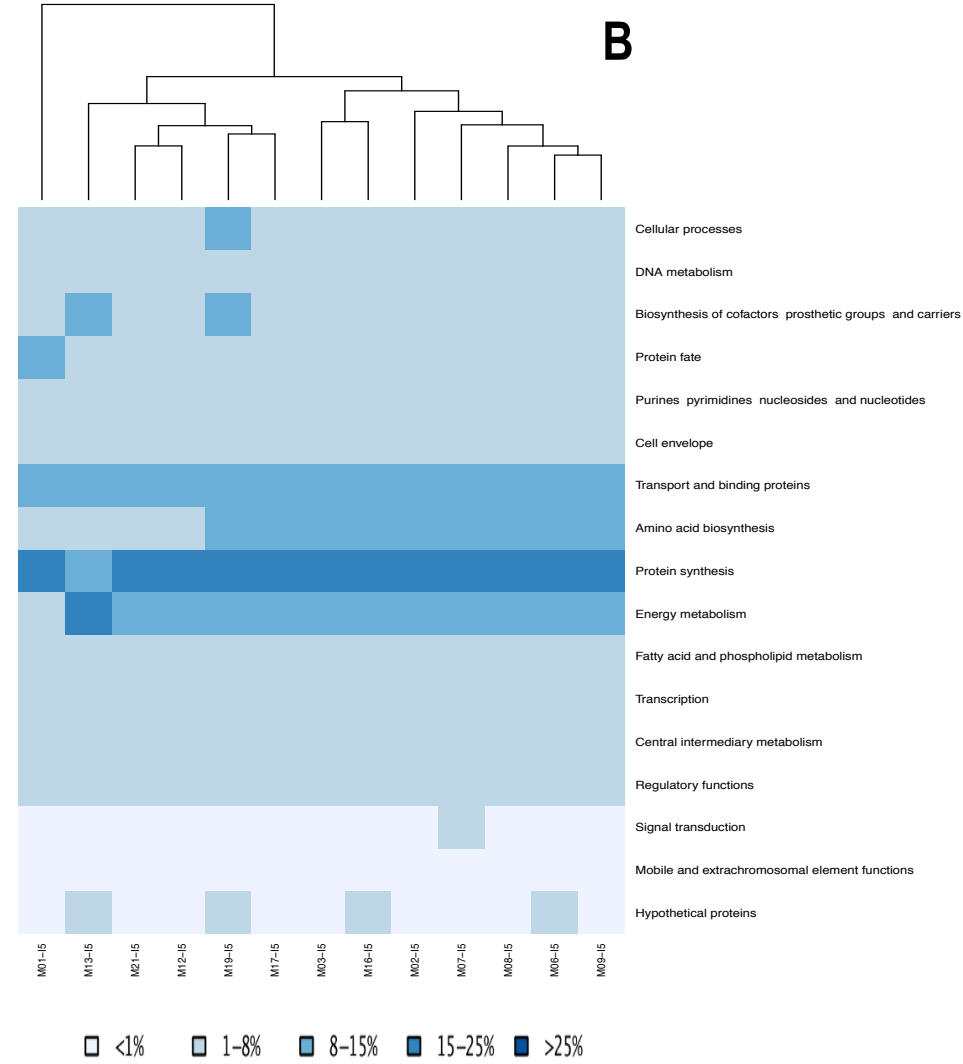

# TP-MA

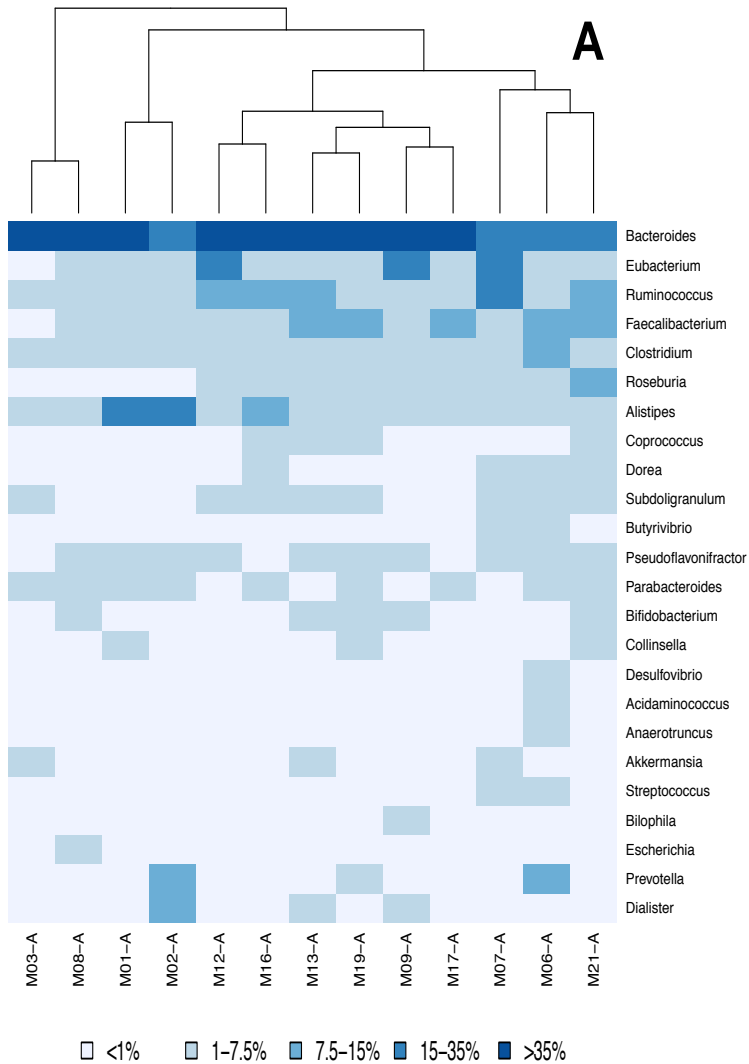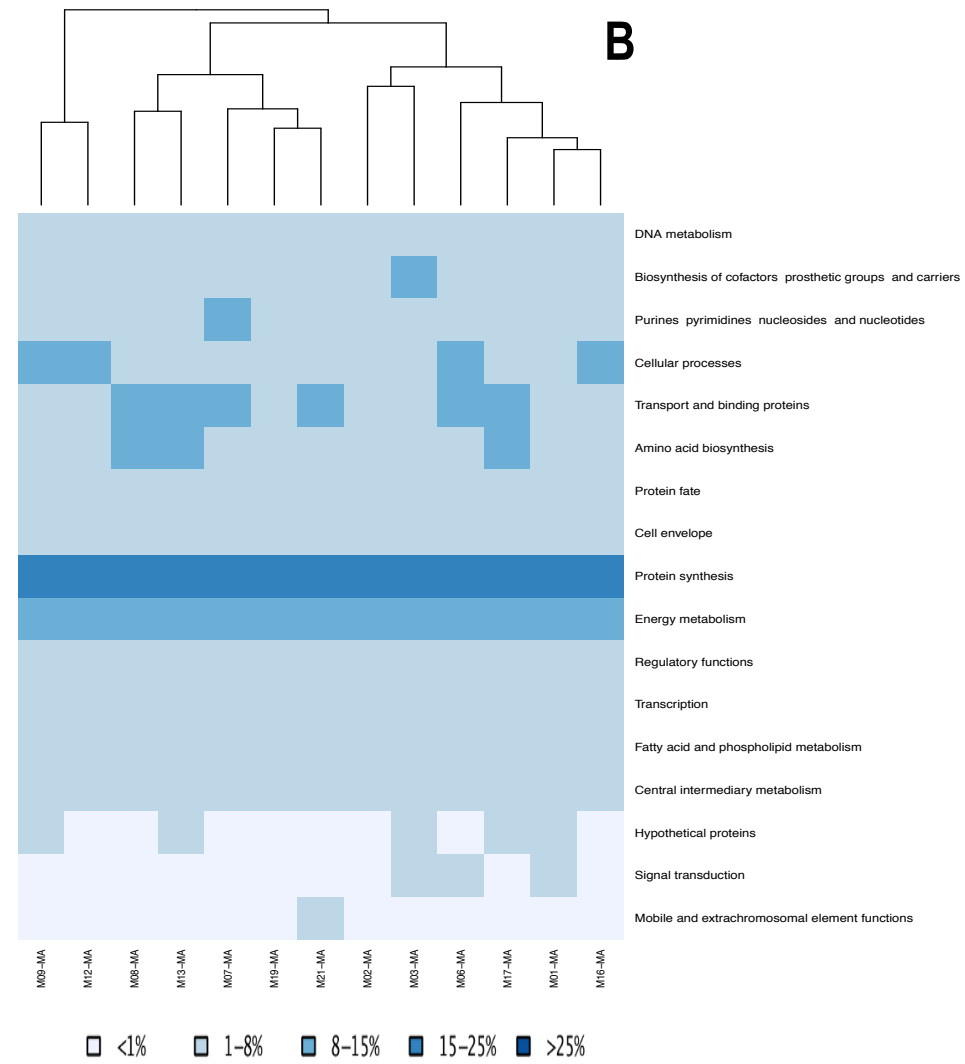

# TP-MB

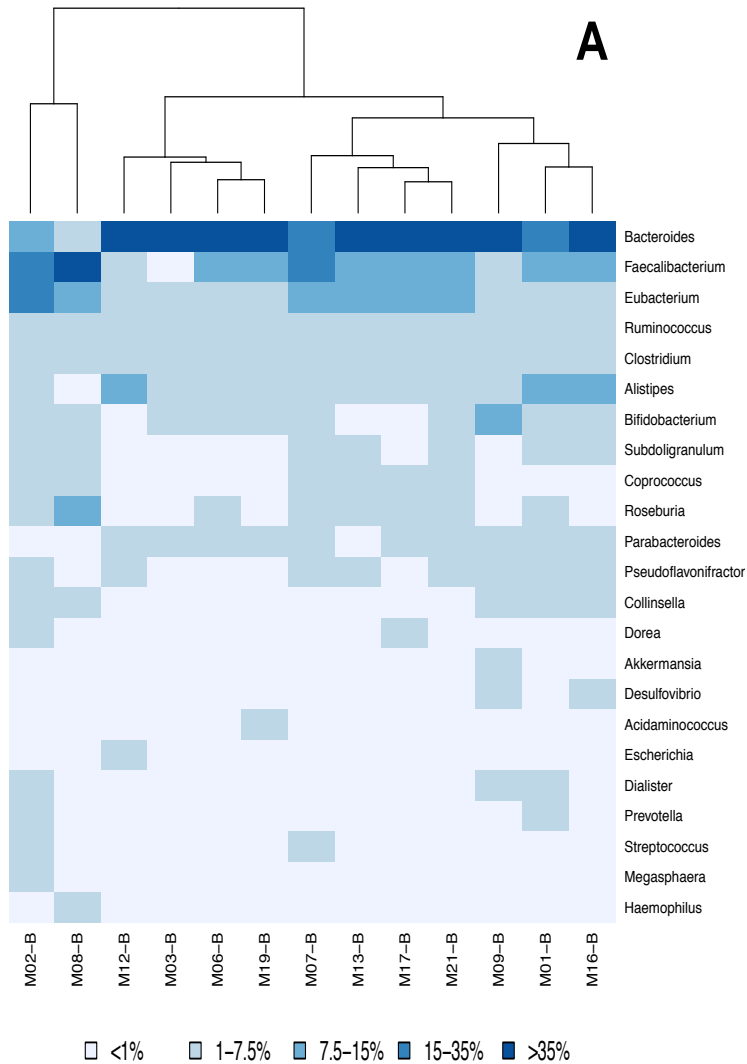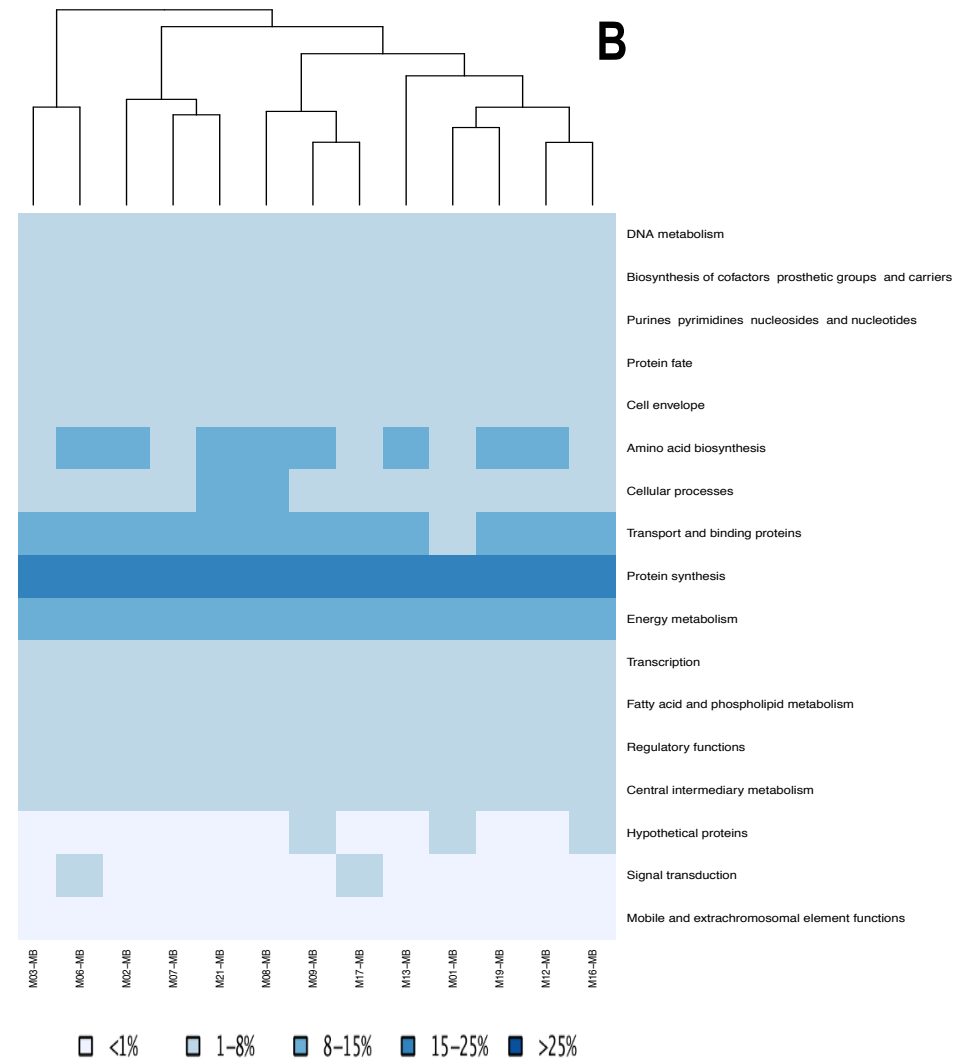

Supplement: Figure S2 — Heatmaps and clustering of the samples for each timepoint according to taxonomic composition (A) and TIGRFAM main functional roles (B) (details as in Figure S1). (PDF) [file pgen.1004406.s002.pdf]

# Linear Regression

## Taxonomic

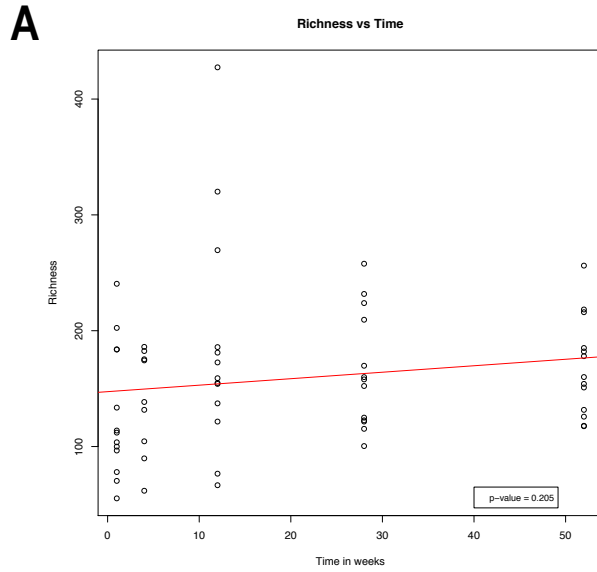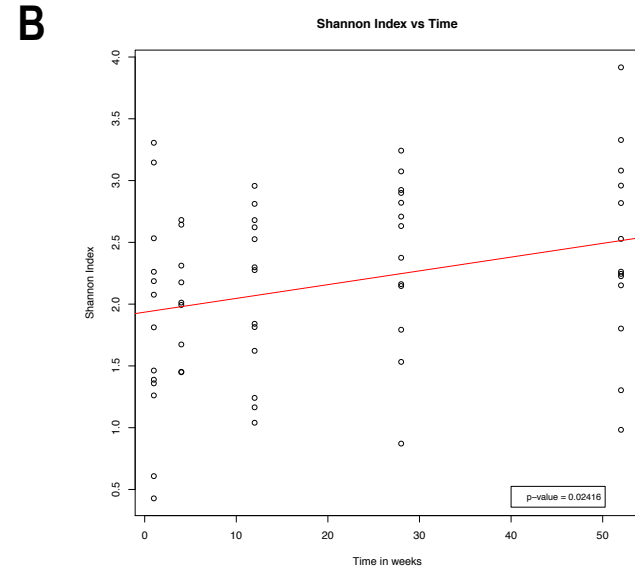

## Functional

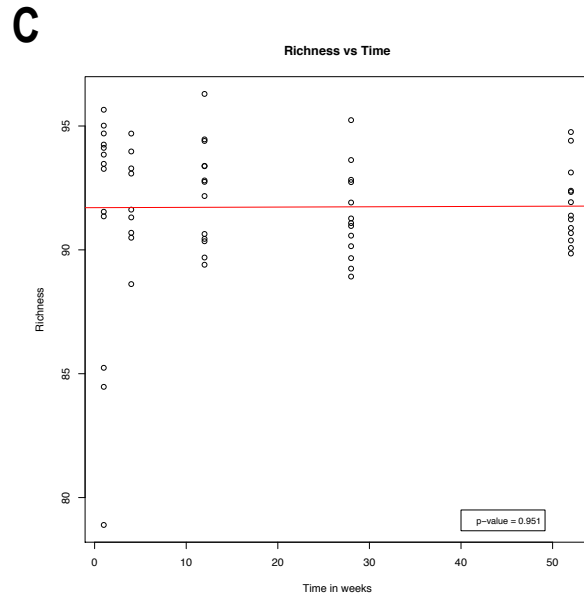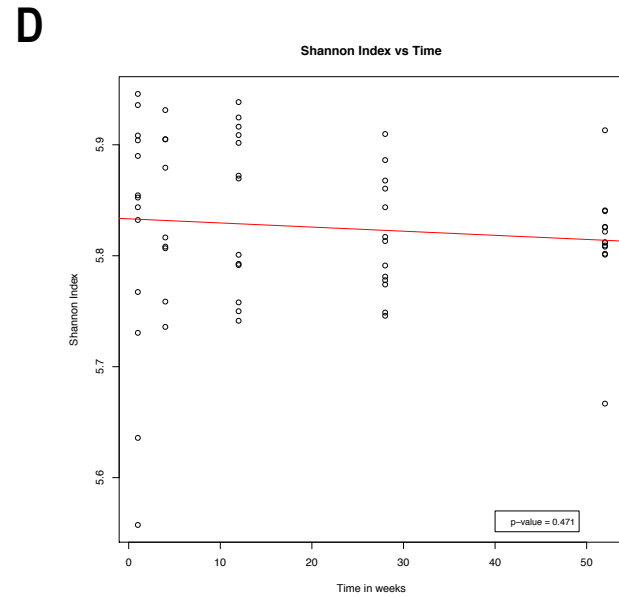

Supplement: Figure S4 — Linear regressions of richness (Chao1 estimator) and diversity (Shannon index) vs. time (A–B taxonomy, C–D function). (PDF) [file pgen.1004406.s004.pdf]

# MIP-01

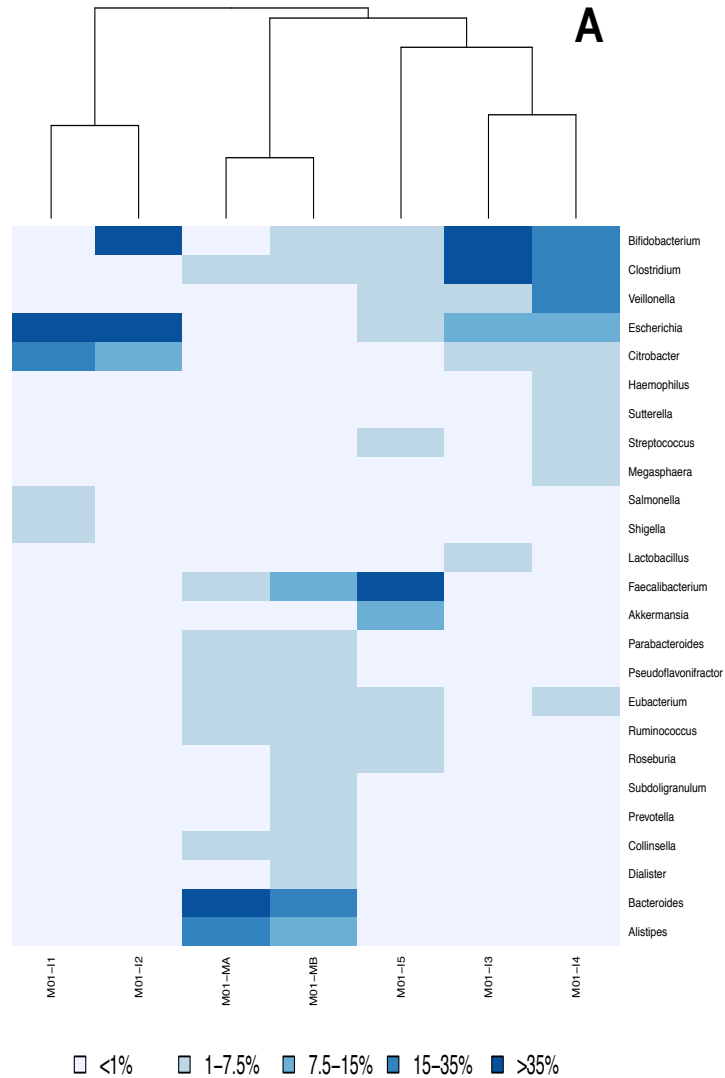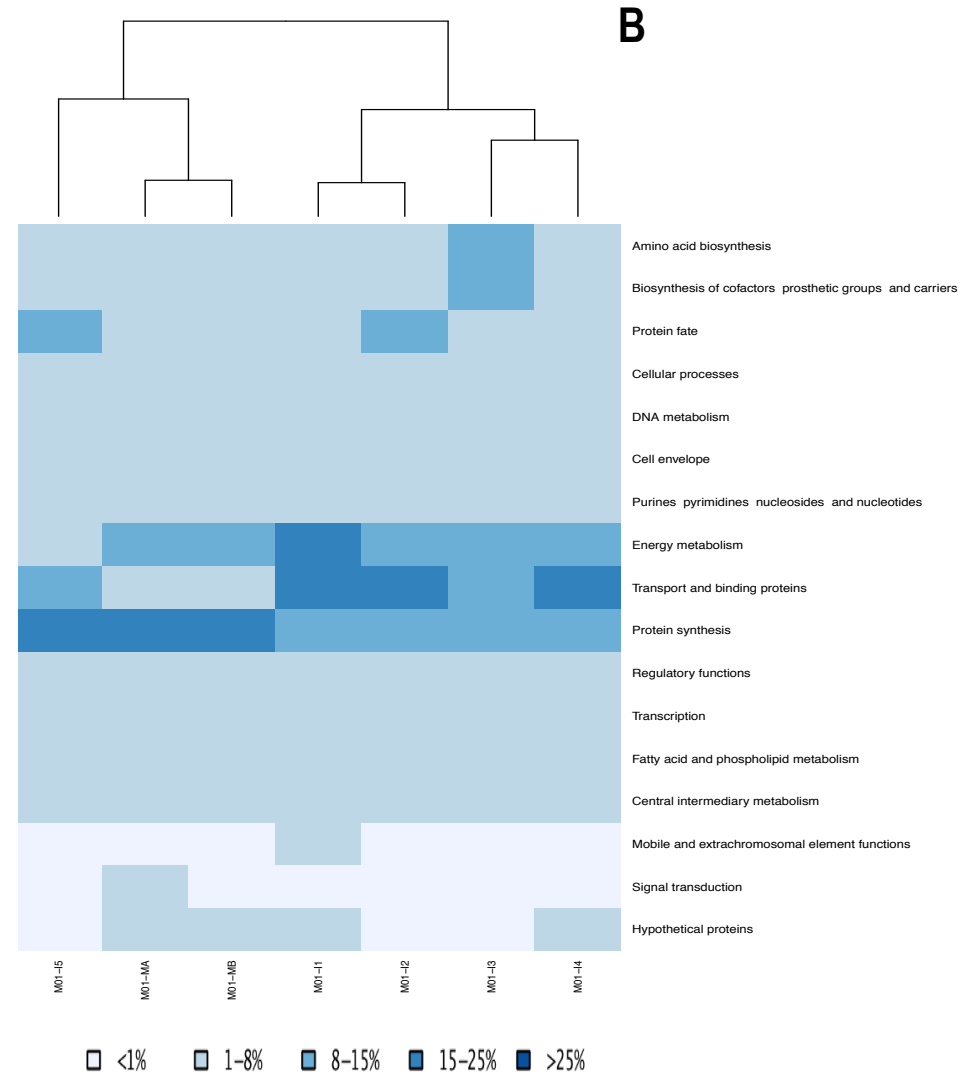

# MIP-02

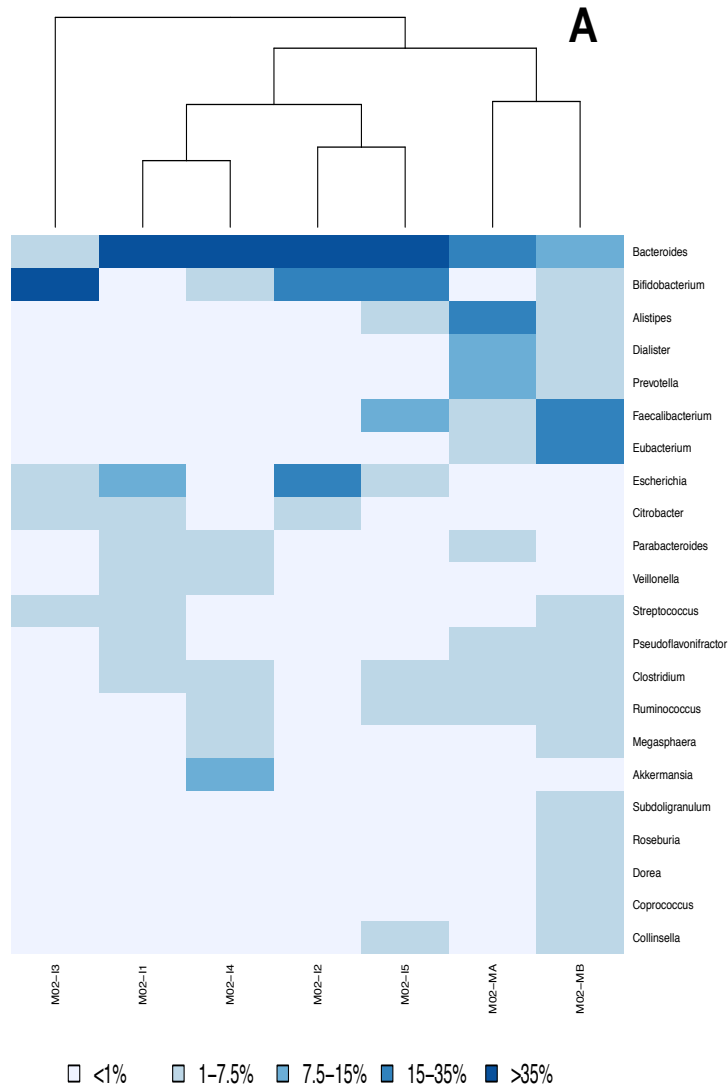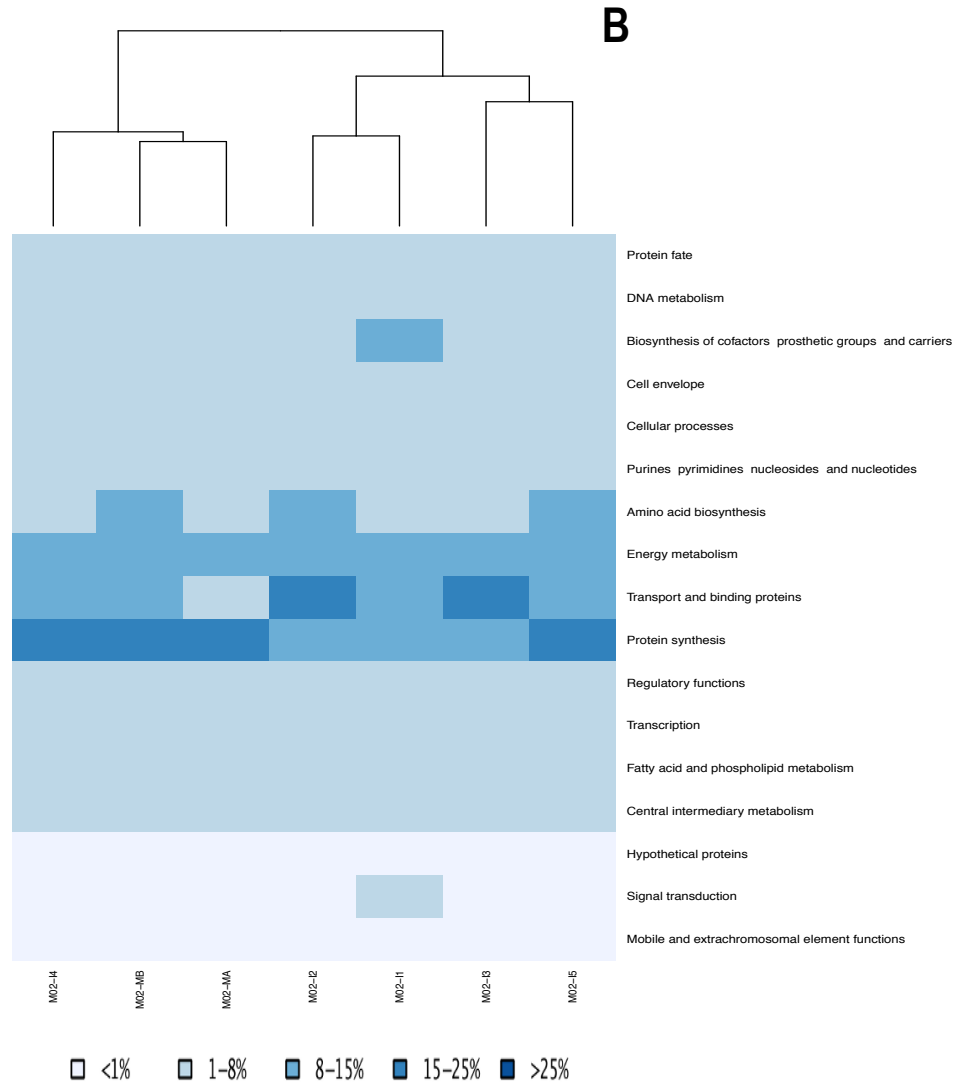

# MIP-03

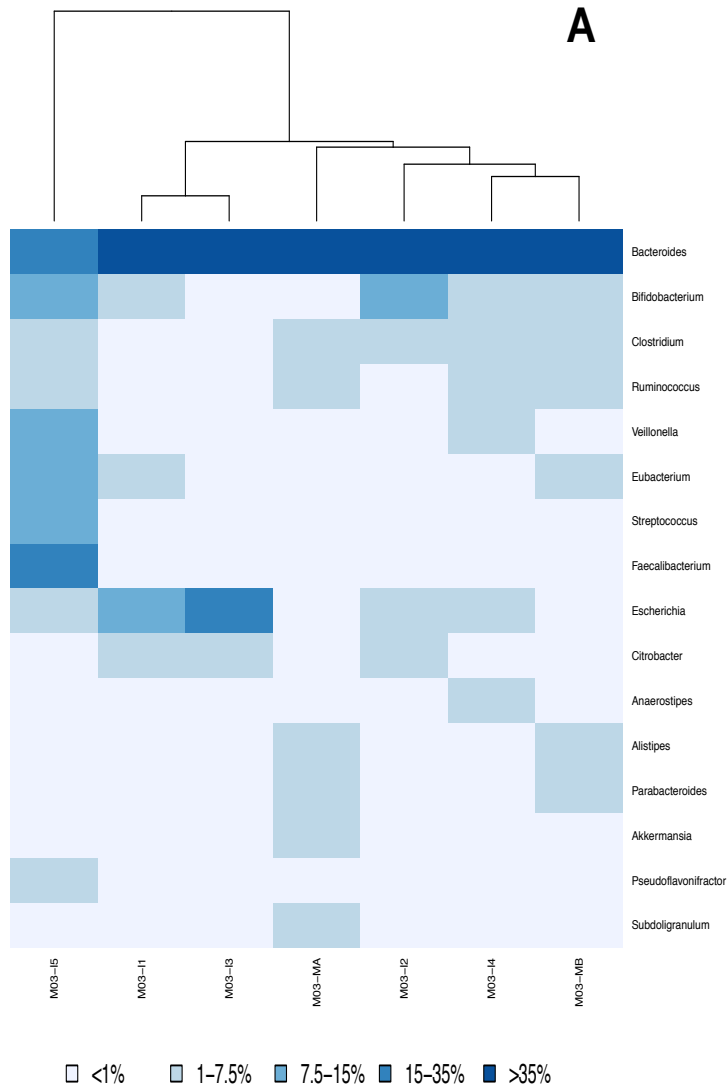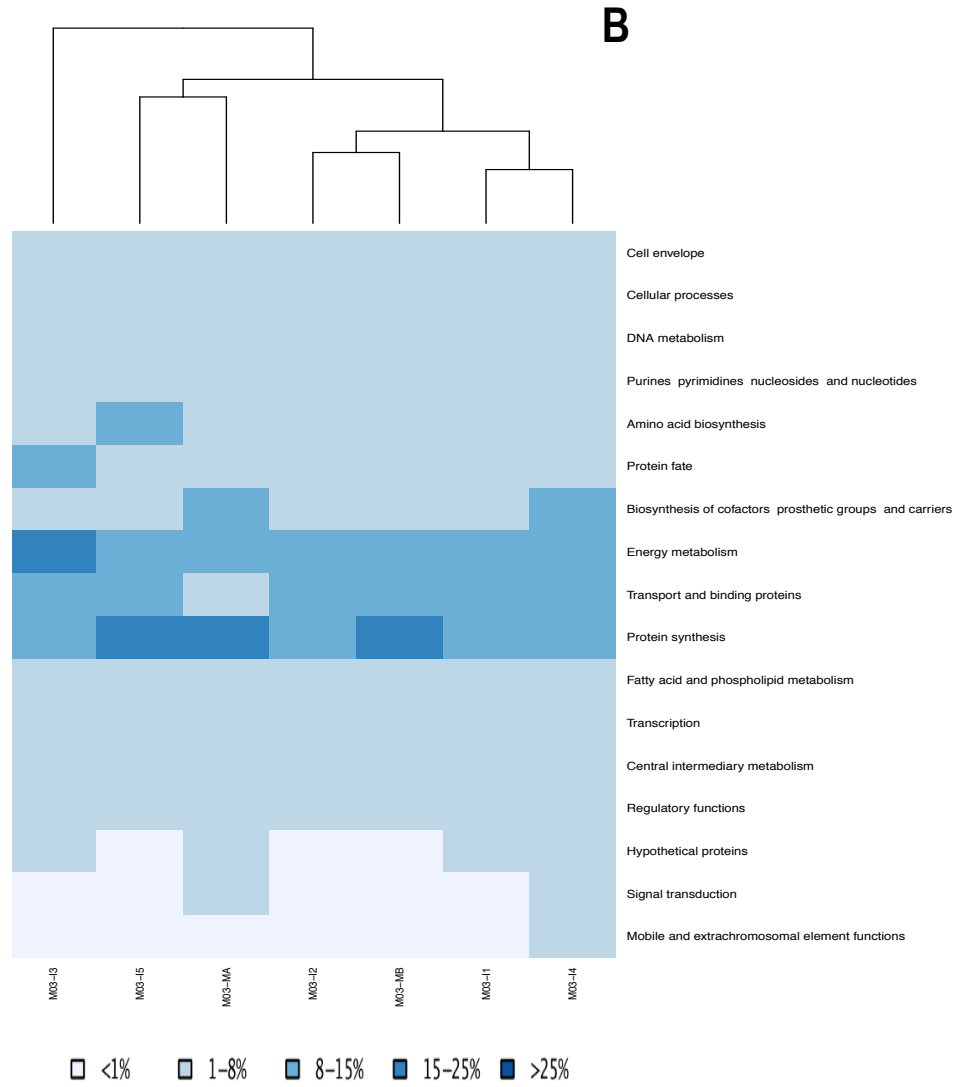

# MIP-06

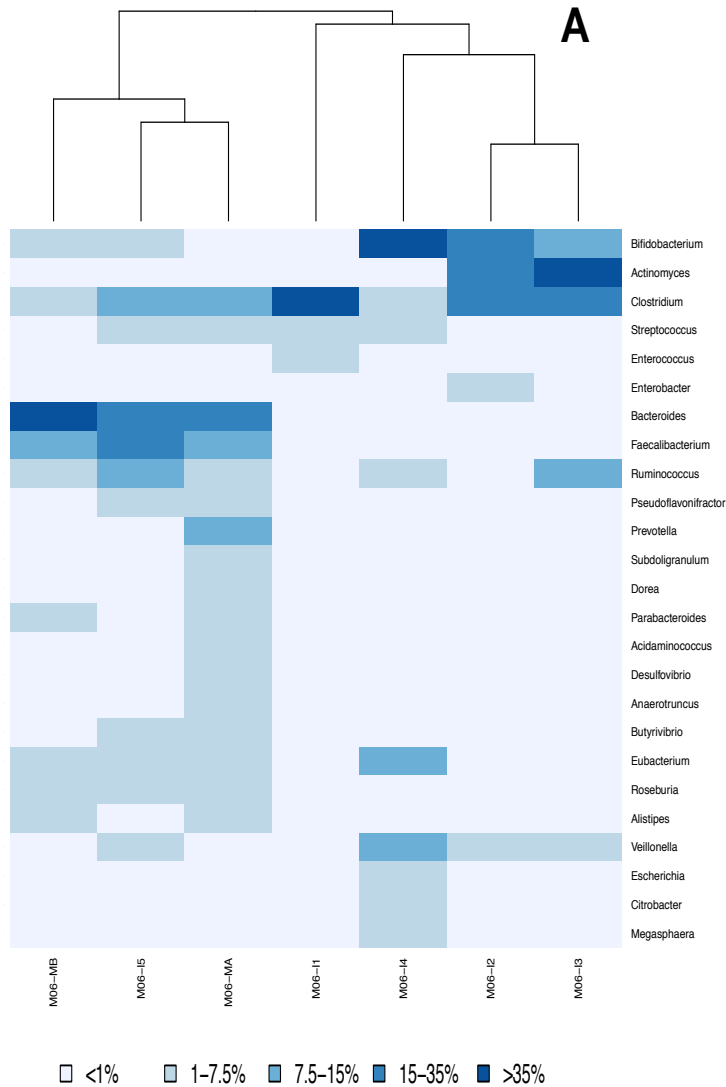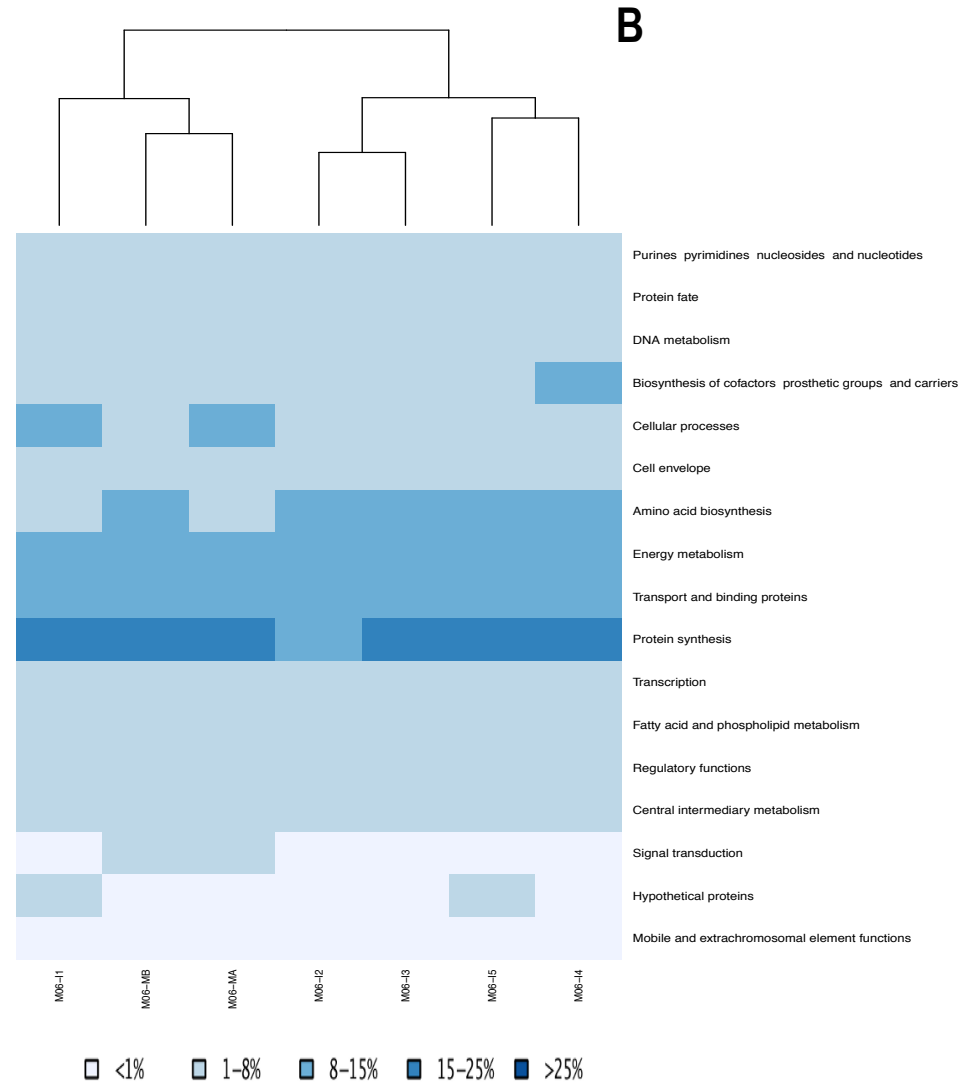

# MIP-07

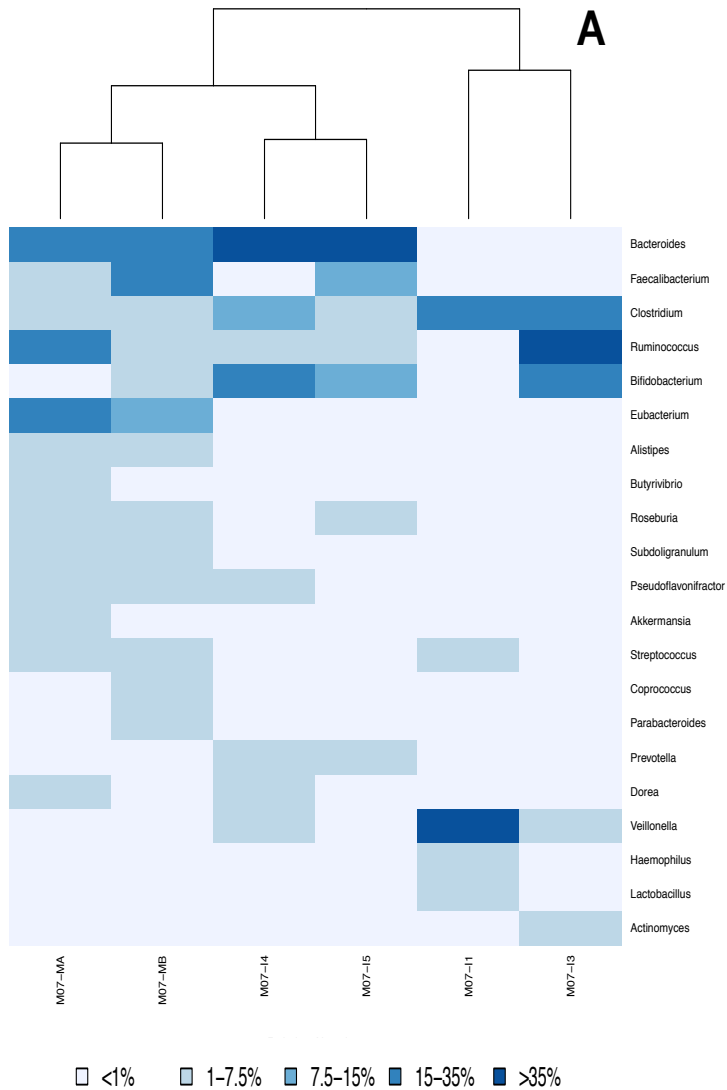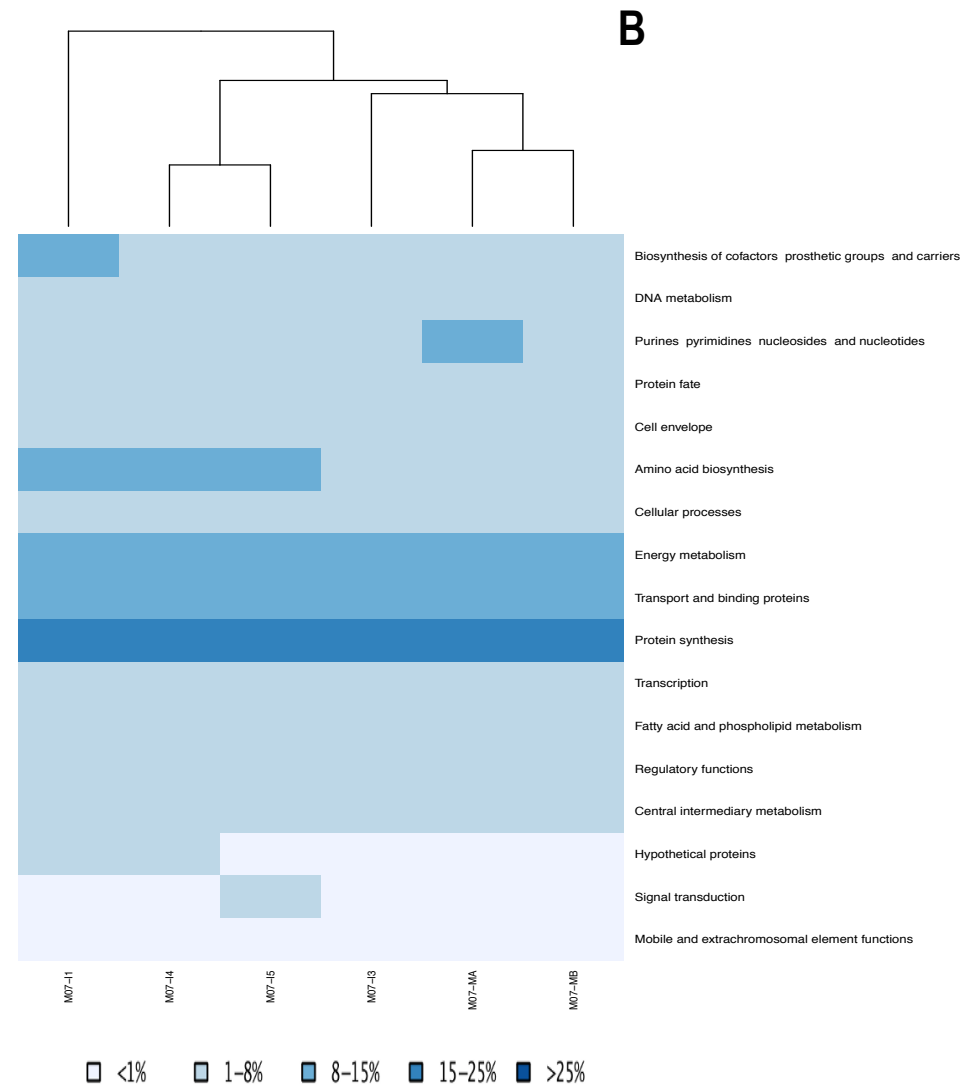

# MIP-08

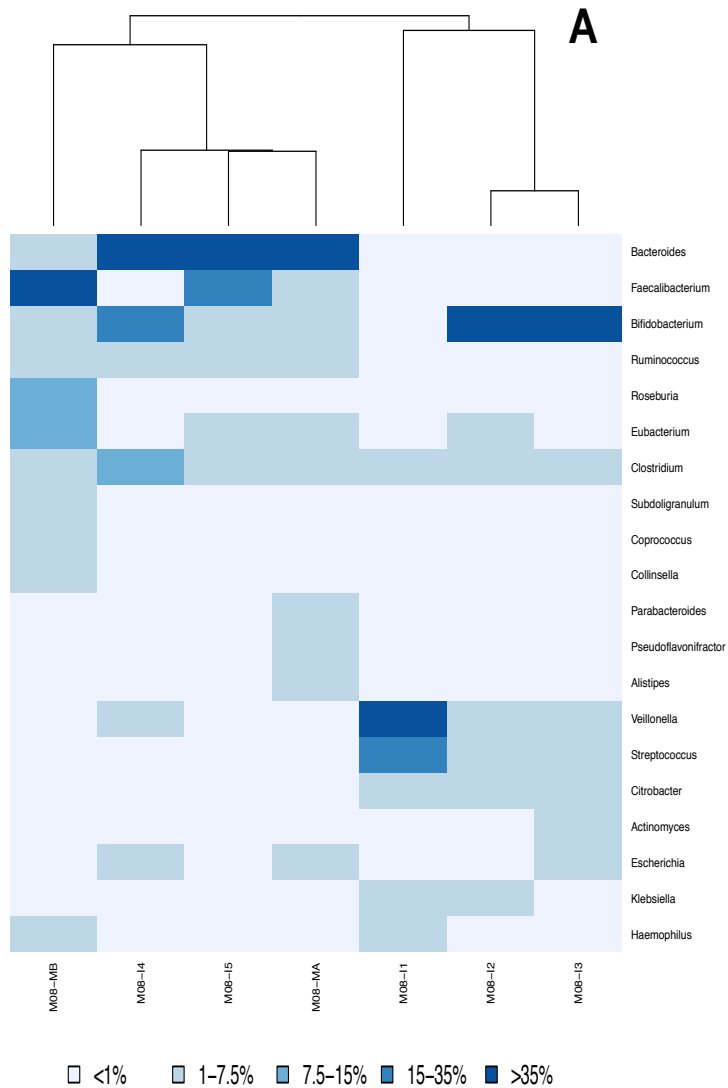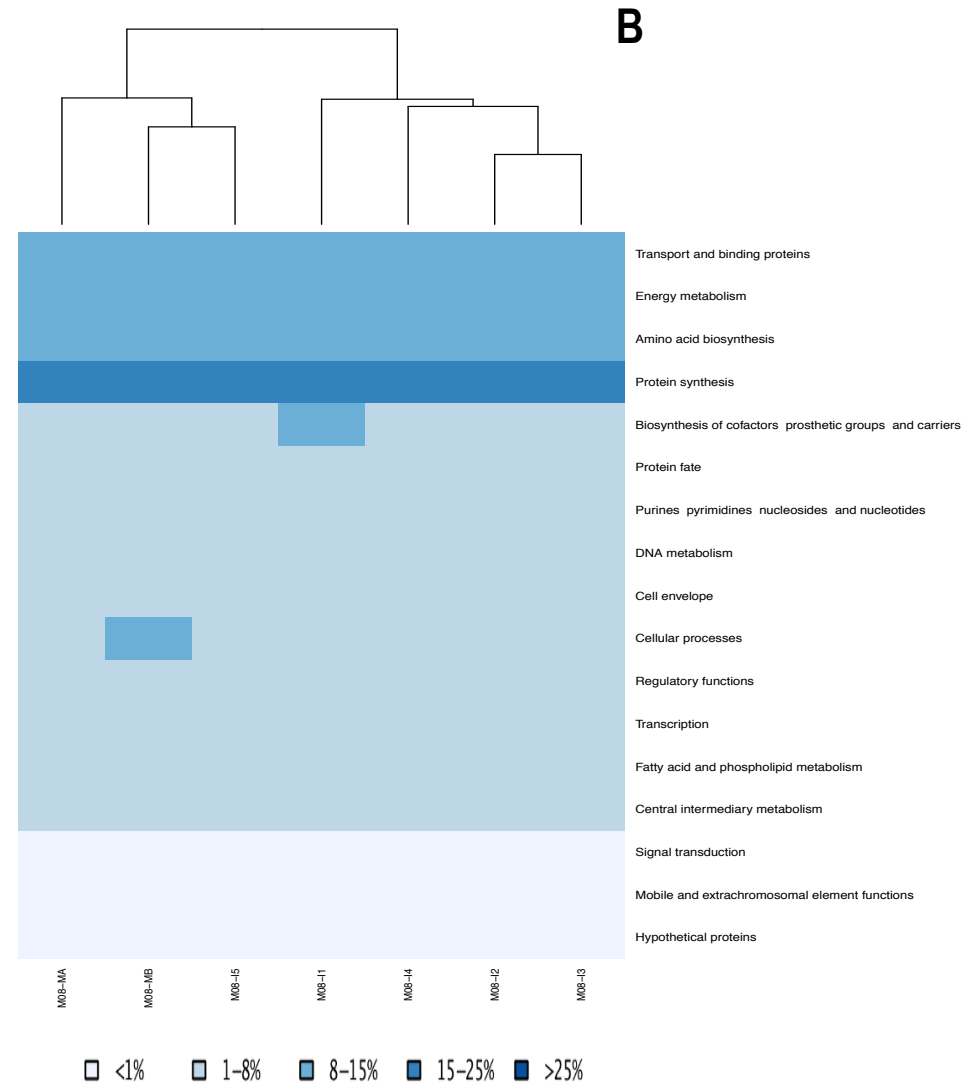

# MIP-09

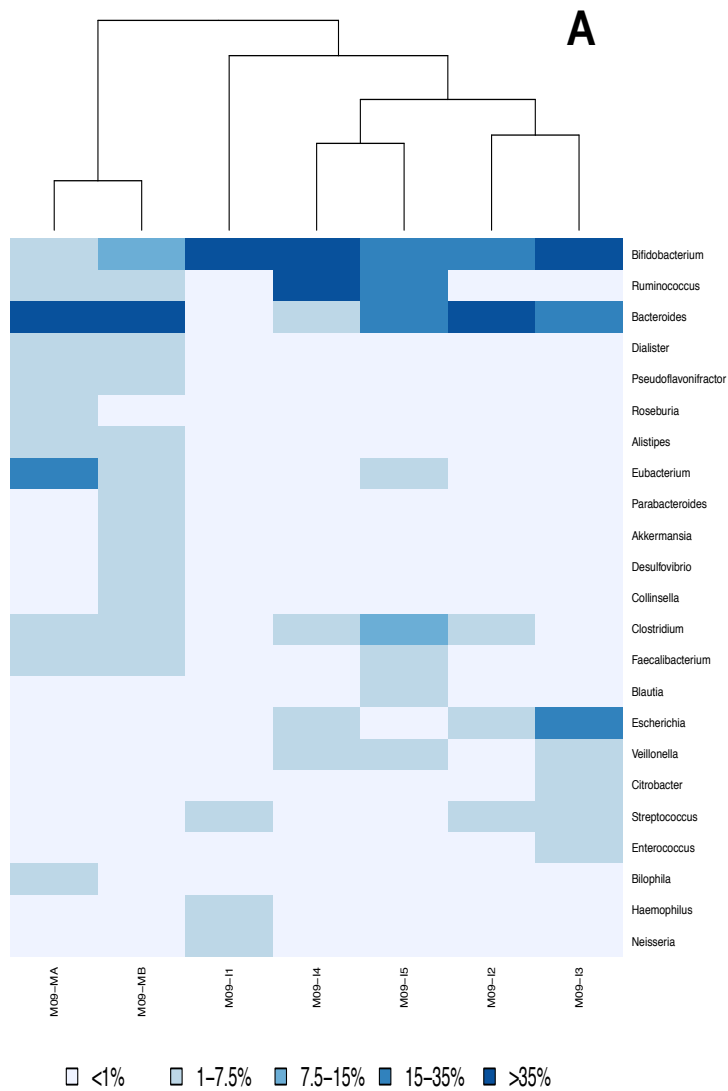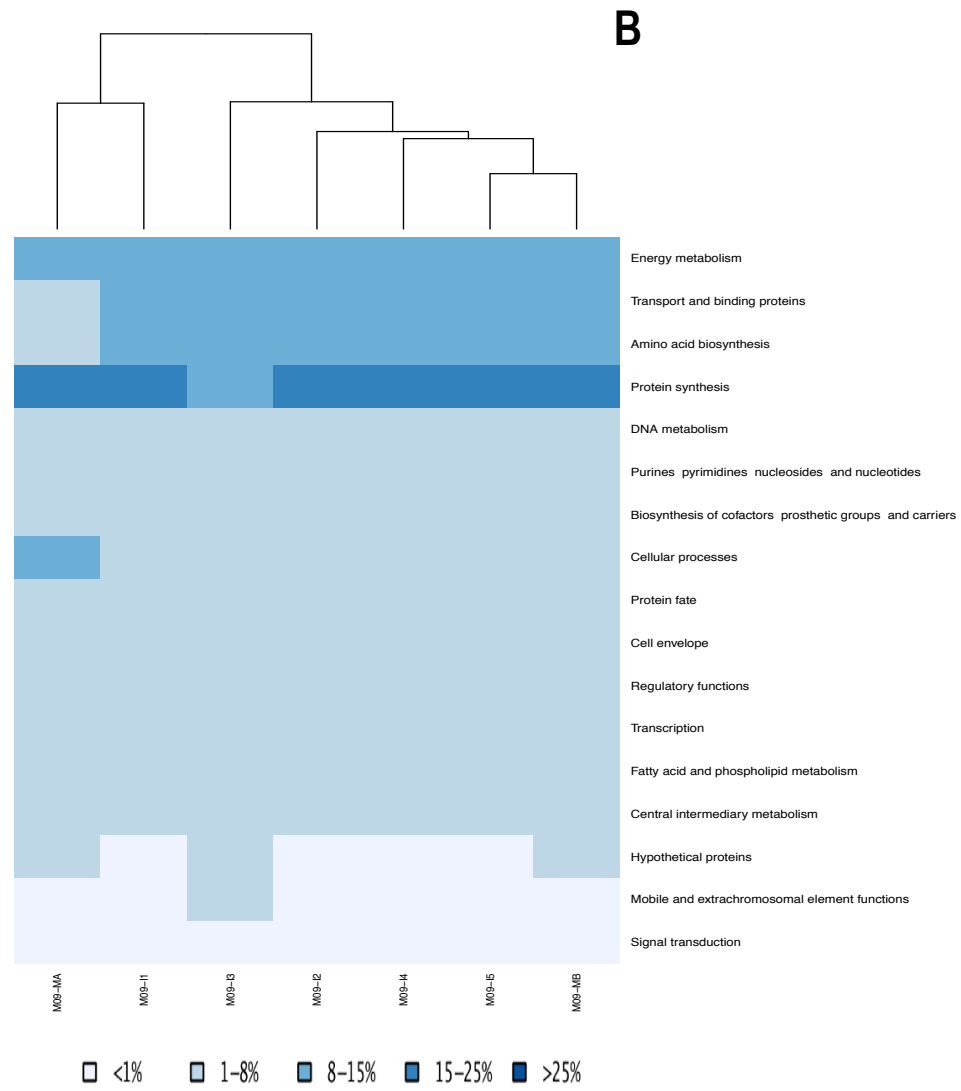

# MIP-12

A

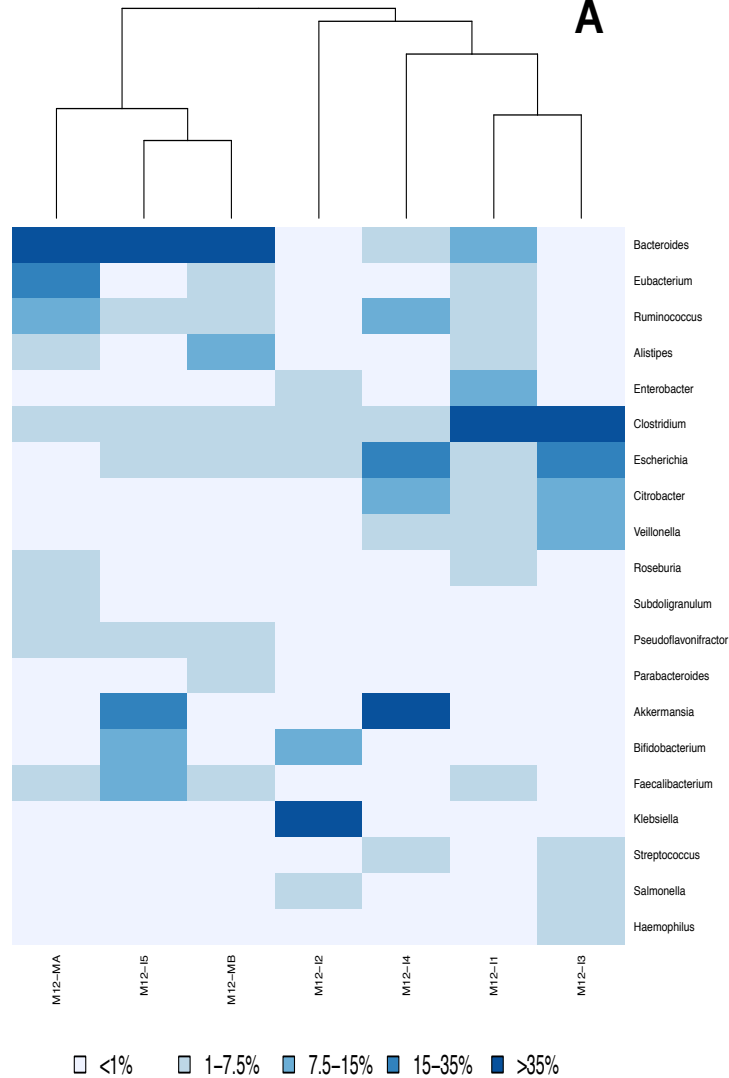

B

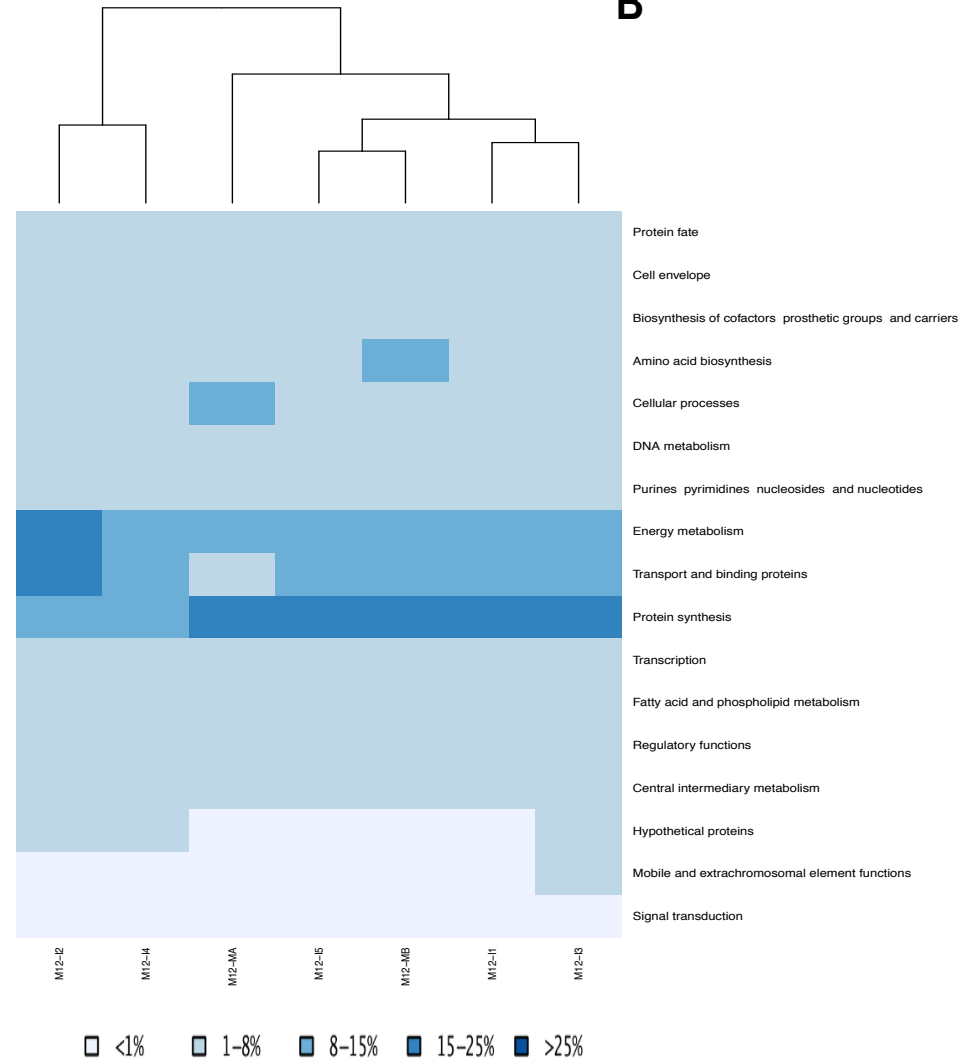

# MIP-13

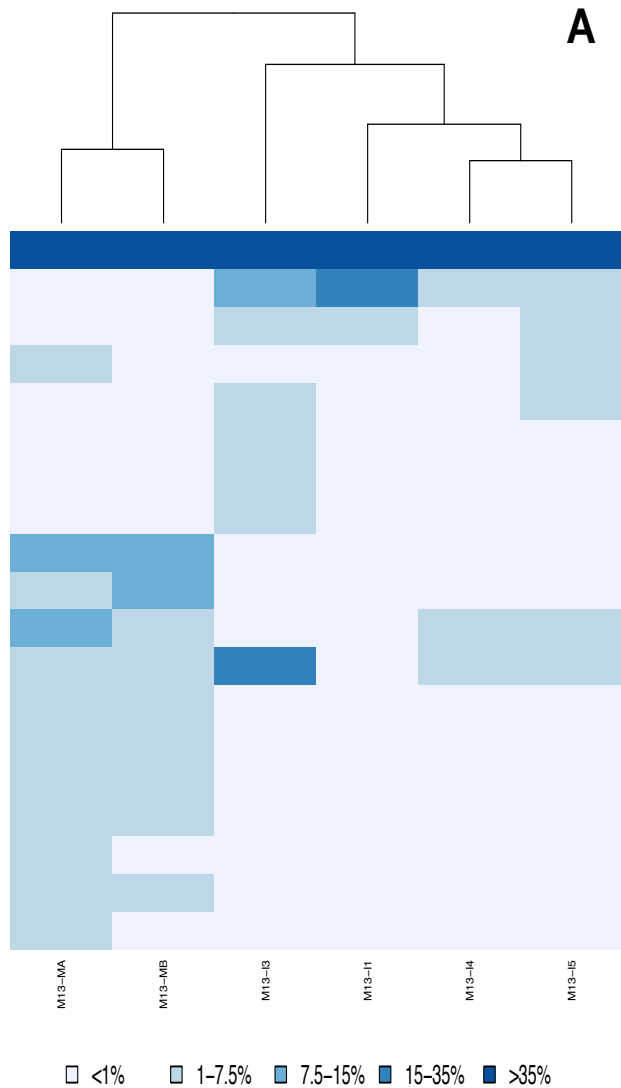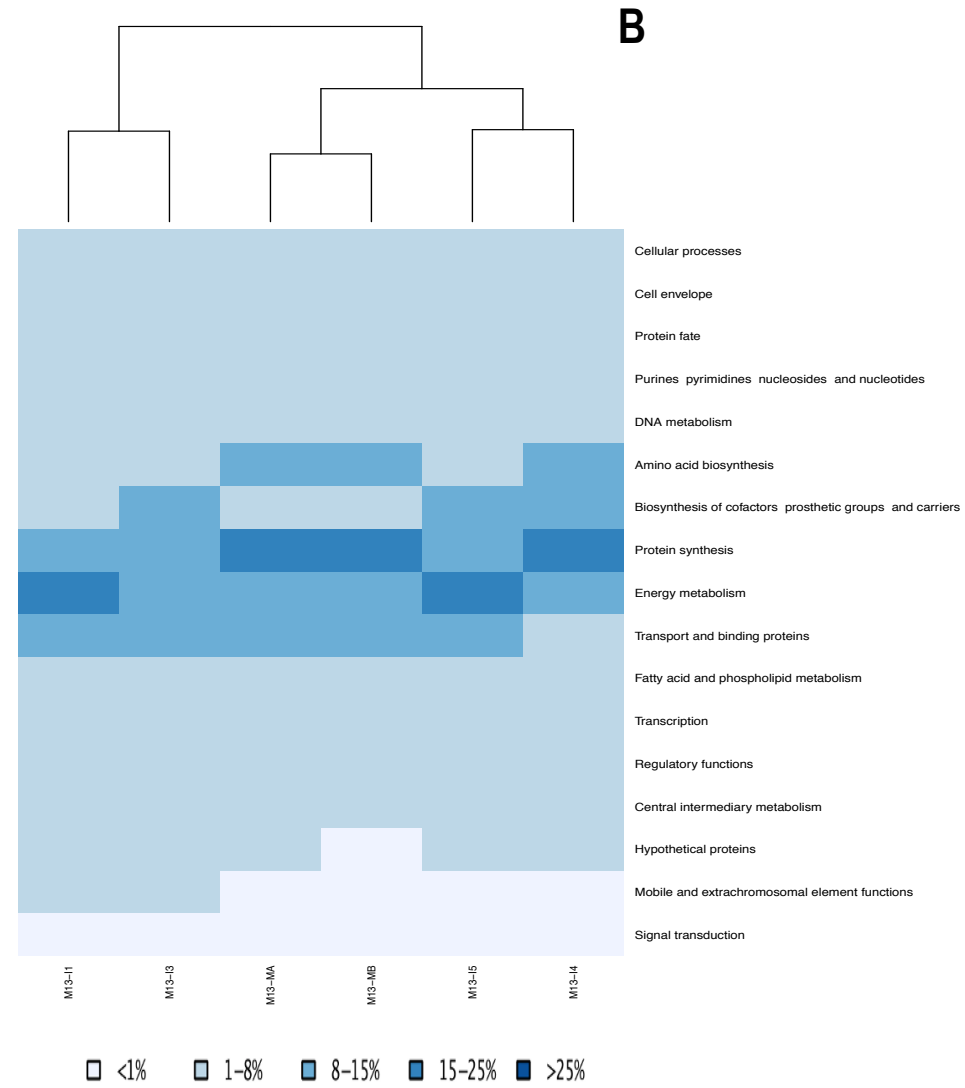

# MIP-16

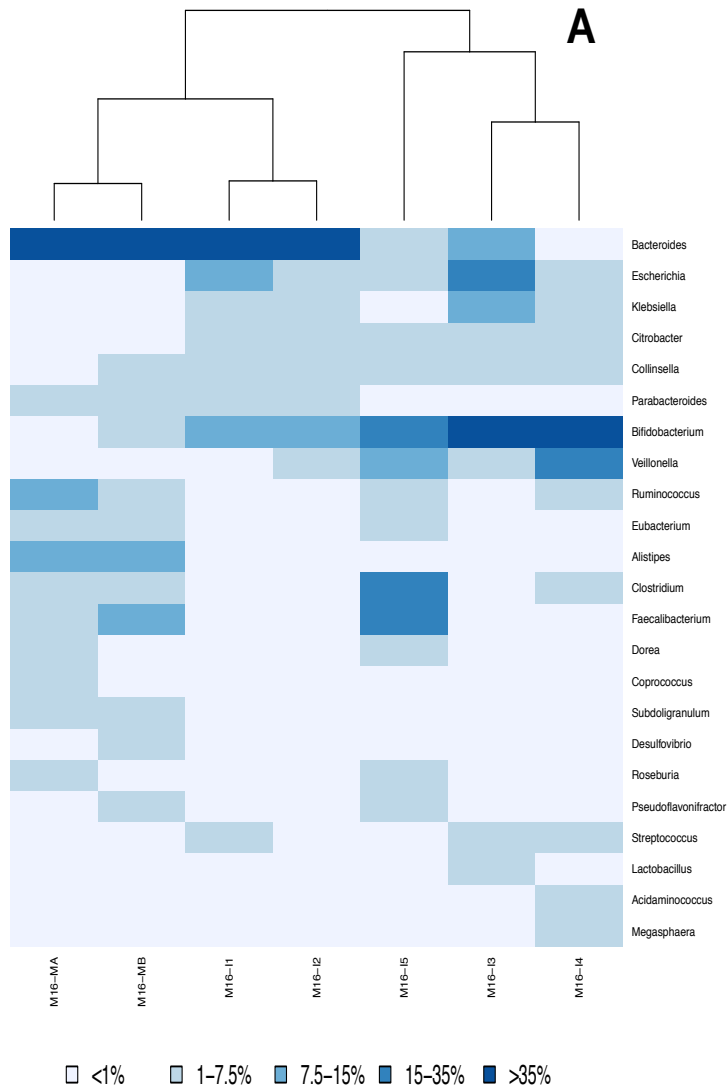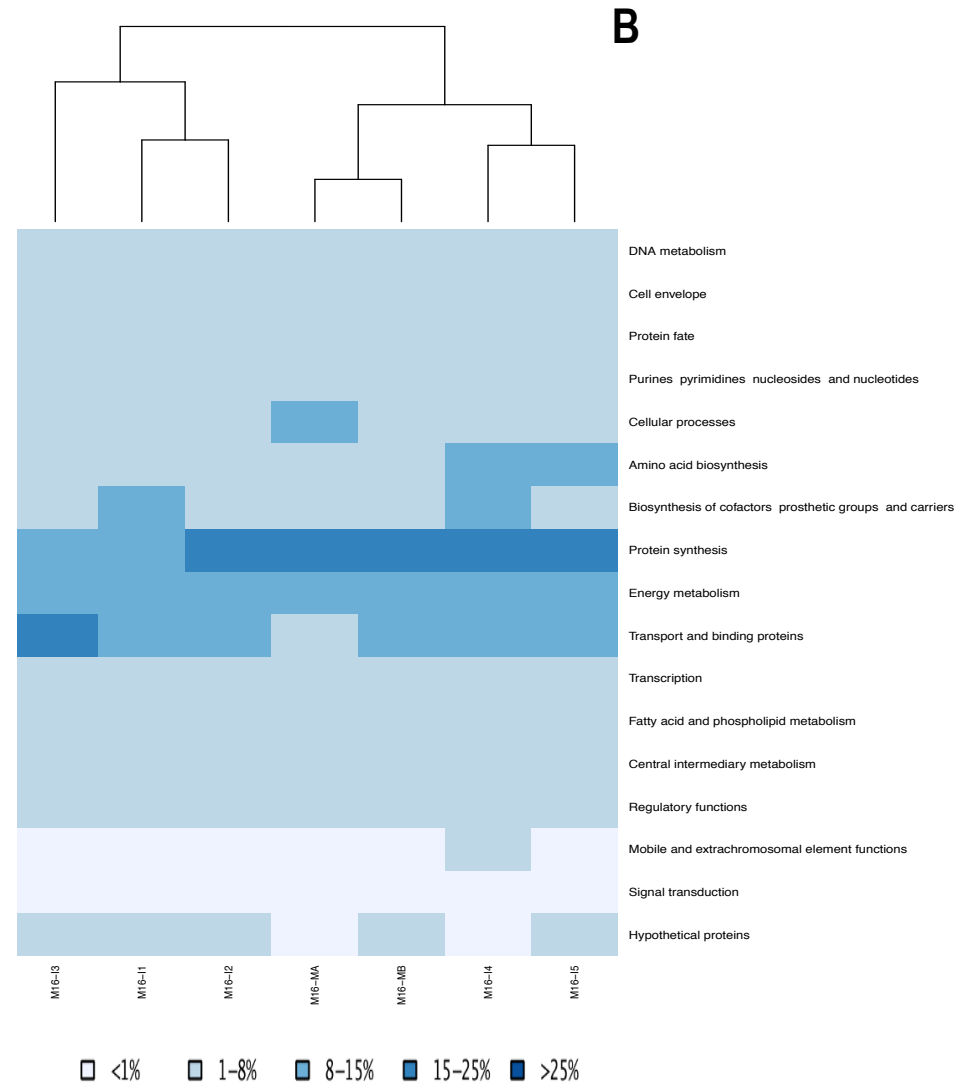

# MIP-17

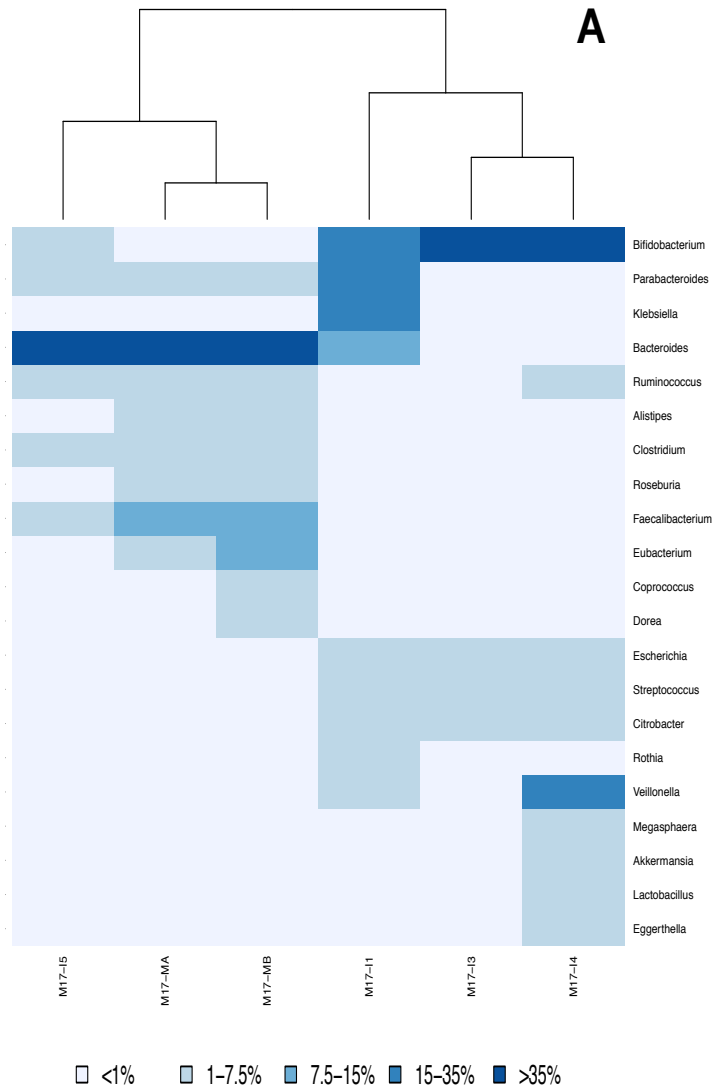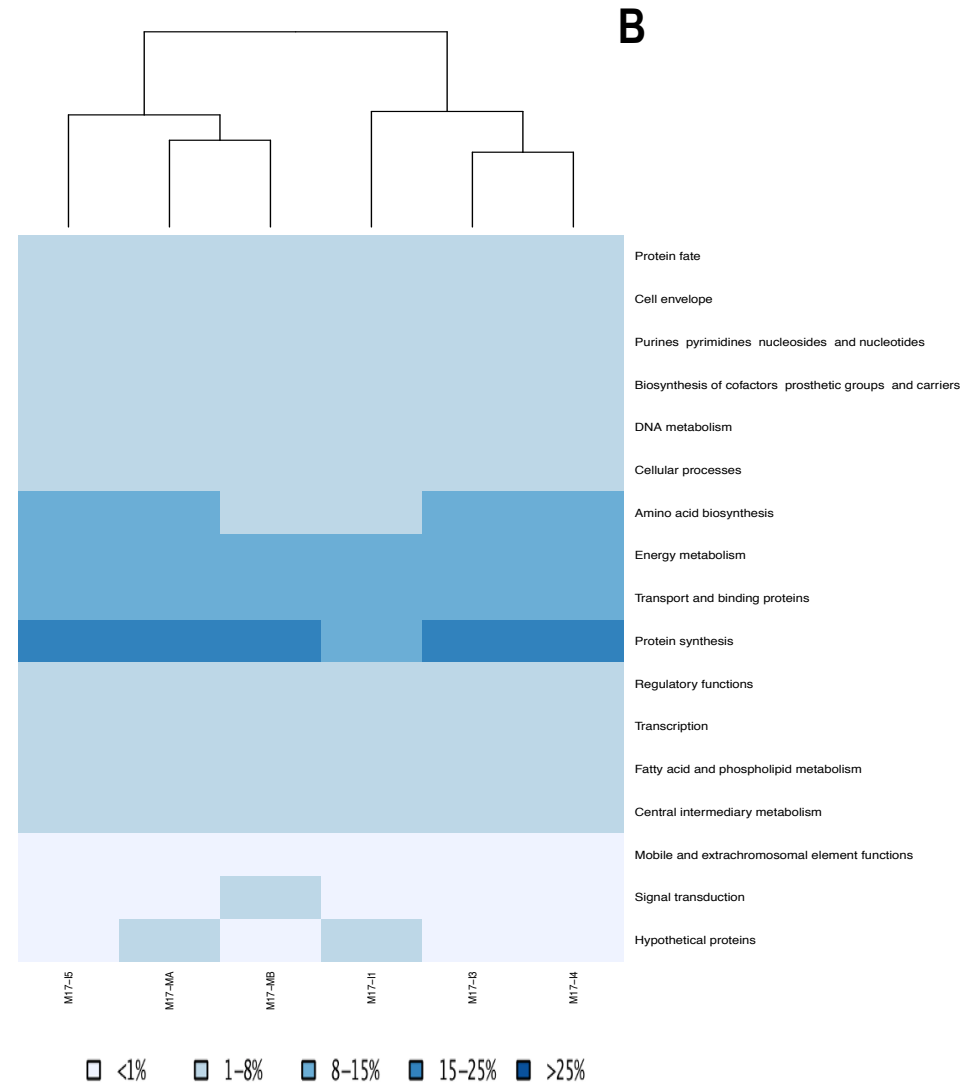

# MIP-19

A

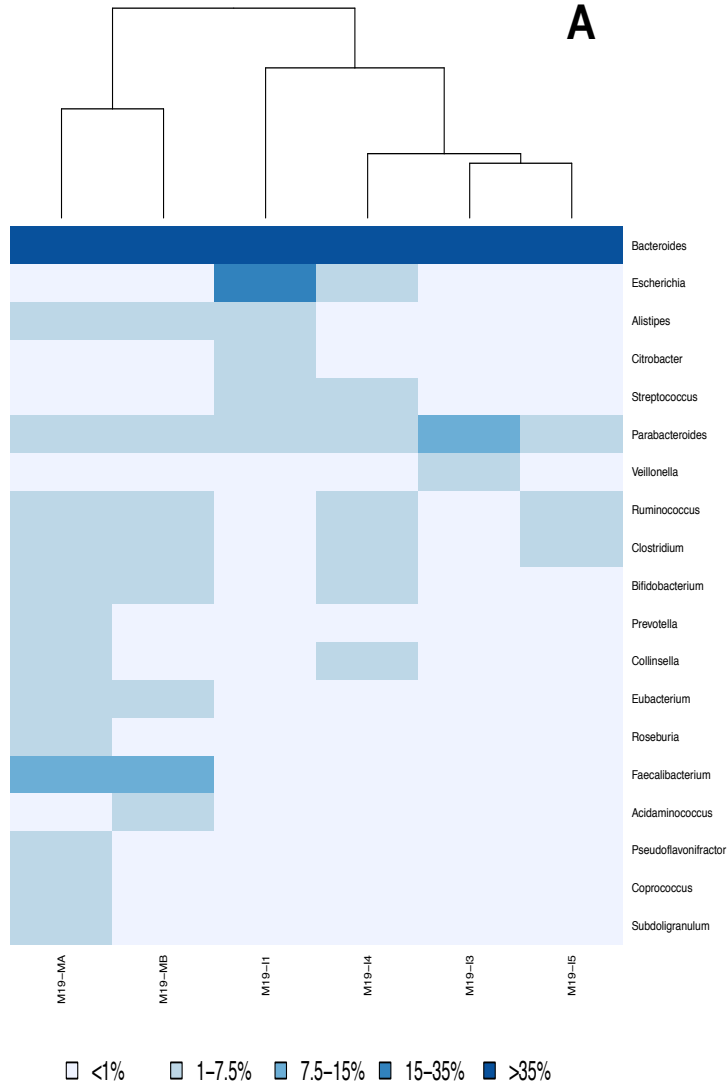

B

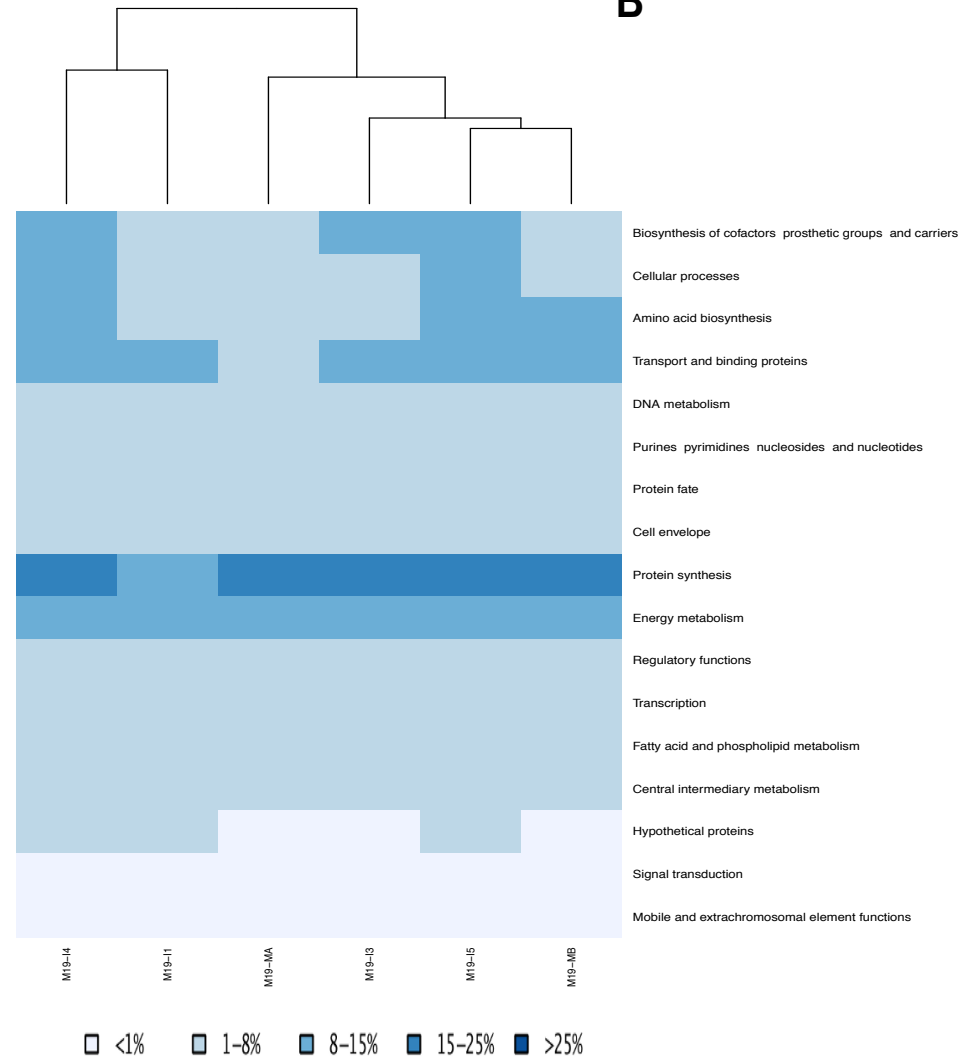

# MIP-21

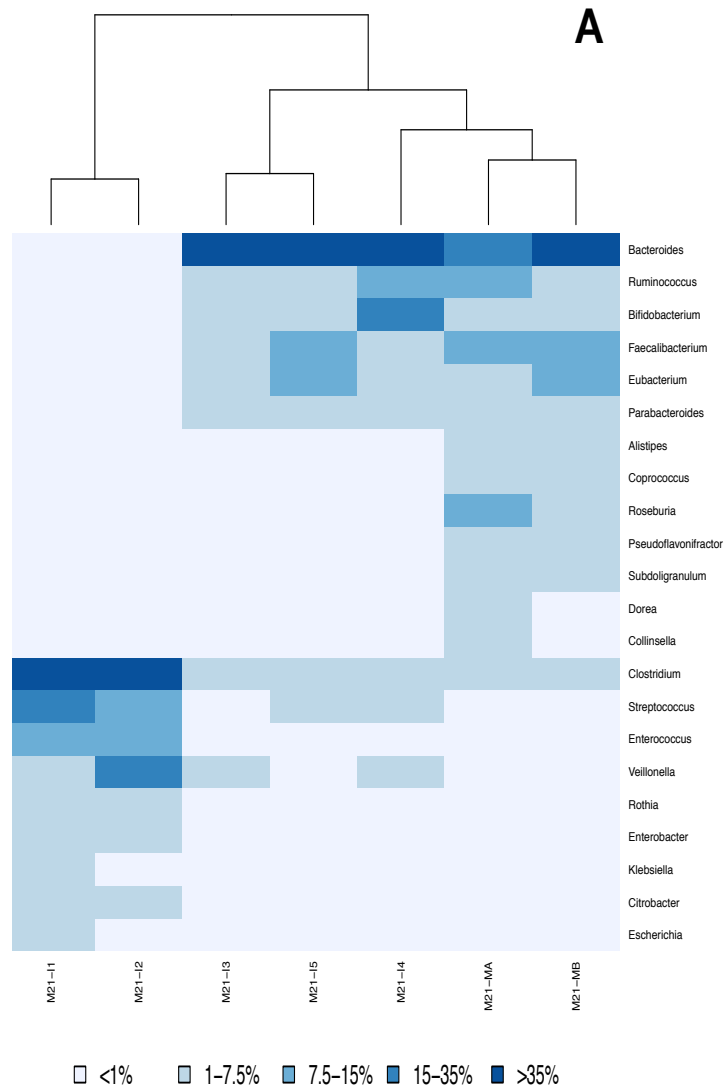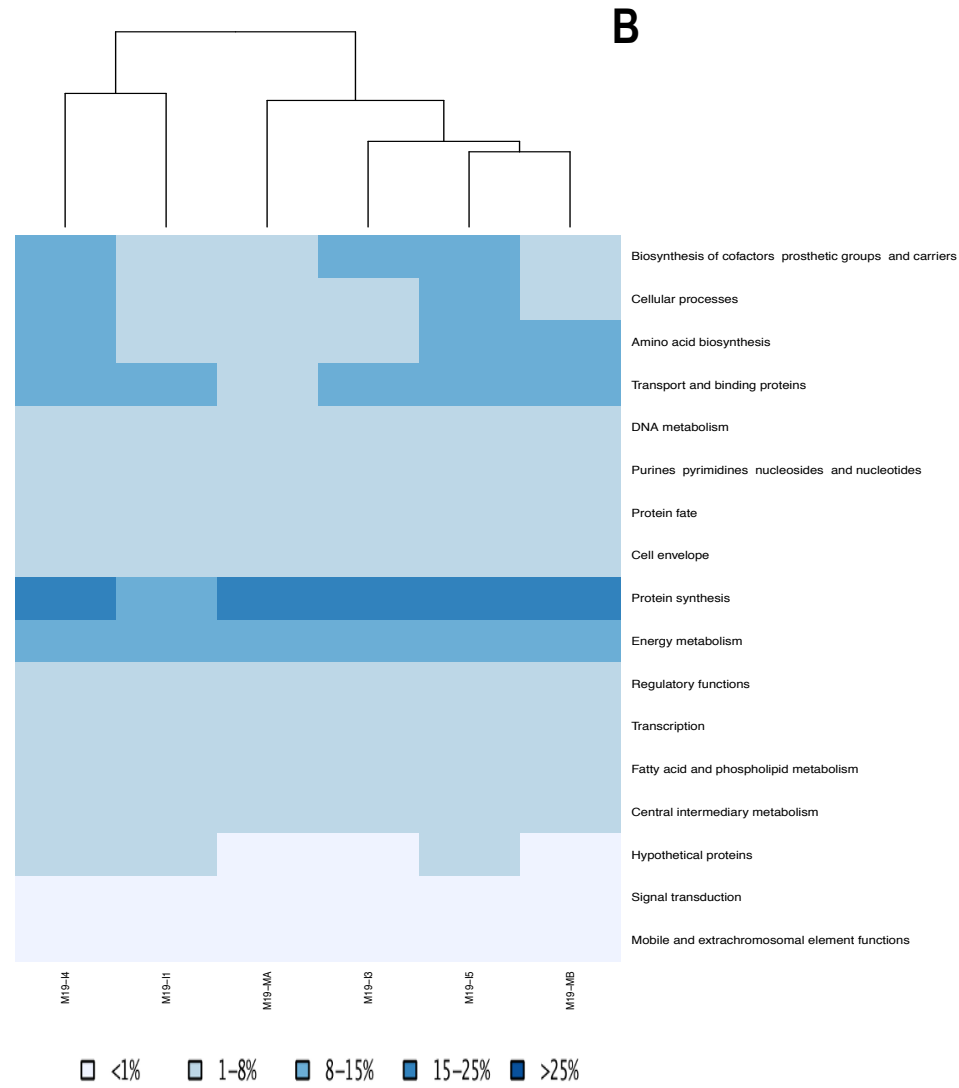

Supplement: Figure S5 — Heatmaps and clustering of the samples for each MIP according to taxonomic composition (A) and TIGRFAM main functional roles (B) (details as in Figure S1). (PDF) [file pgen.1004406.s005.pdf]

**A**

Taxonomic SOM (80% Bootstrap support)

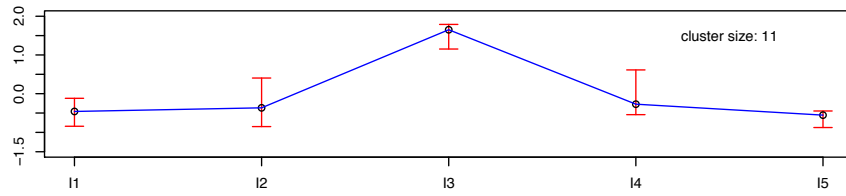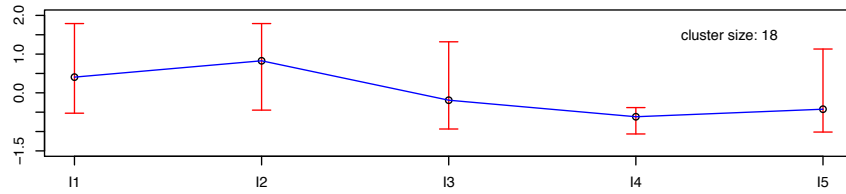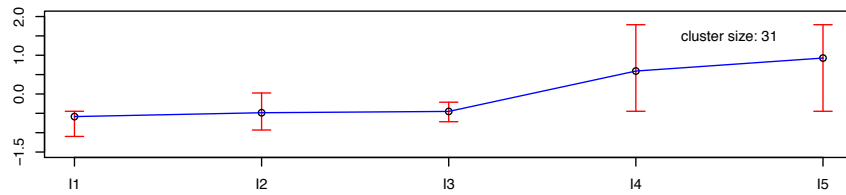**B**

Functional SOM (60% Bootstrap support)

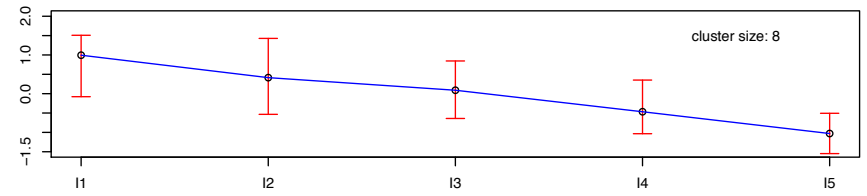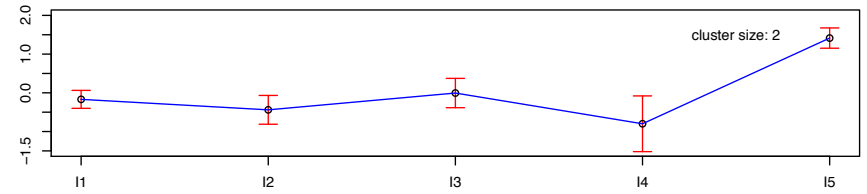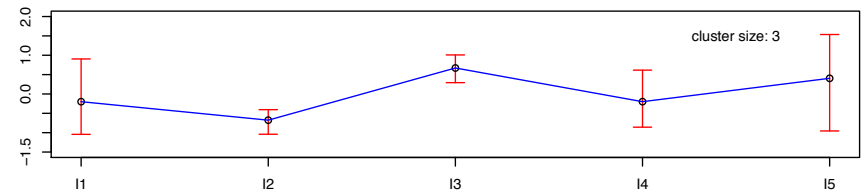

Supplement: Figure S6 — Self-Organizing Maps (SOM) of taxon and function dynamics. SOMs identify patterns of abundance dynamics in the infants throughout development at both taxonomic (A) and functional (B) levels. The number of genera (A) or functions (B) included in each represented cluster is indicated (cluster size). Clusters have 80% and 60% bootstrap support for taxa and functions, respectively. For each cluster, average values on each timepoint along with their corresponding 95% confidence intervals are shown, in a scale centered at the mean of all samples and scaled by the standard deviation. (PDF) [file pgen.1004406.s006.pdf]
