# Supplementary material for: Microbial Succession in the Gut: Directional Trends of Taxonomic and Functional Change in a Birth Cohort of Spanish Infants
Source: PLoS Genet. 2014 Jun 5;10(6):e1004406. doi: 10.1371/journal.pgen.1004406 (PMC4046925; doi:10.1371/journal.pgen.1004406)
Supplement: Figure S3 — Canonical Correspondence Analyses (CCA) showing the effect of C-section on the taxonomic composition of the microbiota at different timepoints. The proportion of variability explained by C-section delivery is highest at I1 (16%), I2 (22%) and I3 (22%) and decreases at I4 (10%) and I5 (10%), and is always below the proportion of variability explained by the first unconstrained axis. (PDF) [file pgen.1004406.s003.pdf]

Canonical Correspondence Analysis: Delivery mode

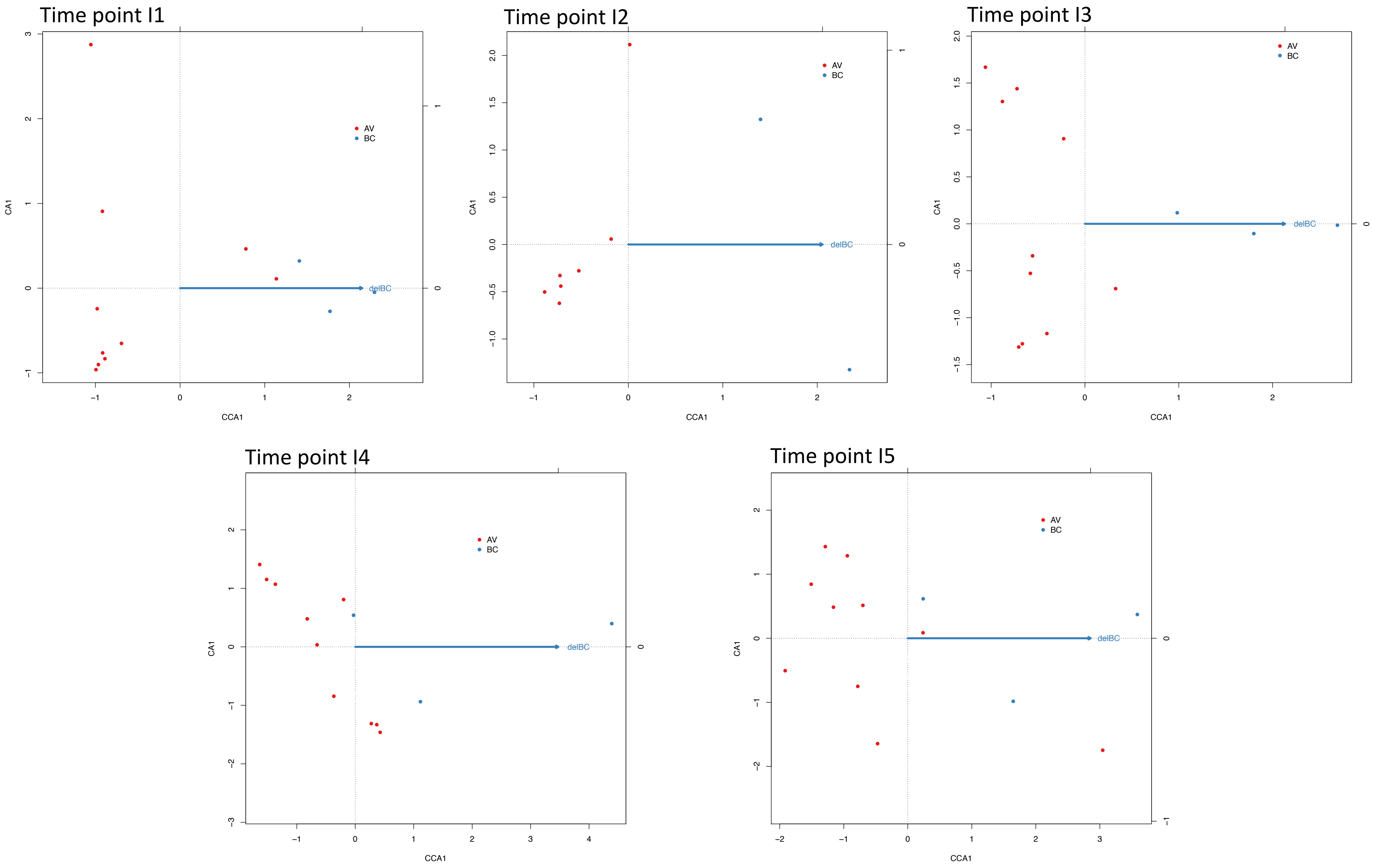

AV: Vaginal Delivery; BC: C-section
