# Supplementary material for: Microbial Succession in the Gut: Directional Trends of Taxonomic and Functional Change in a Birth Cohort of Spanish Infants
Source: PLoS Genet. 2014 Jun 5;10(6):e1004406. doi: 10.1371/journal.pgen.1004406 (PMC4046925; doi:10.1371/journal.pgen.1004406)
Supplement: Table S1 — Information on consumption of different foods, obtained from questionnaires answered by the infants' parents. (DOCX) [file pgen.1004406.s007.docx]

|  | ***Consumers at 7 months*** | ***Consumers at 1 year*** |
| --- | --- | --- |
| **Breast milk** | 92% | 70% |
| **Formula** | 77% | 91% |
| **Yogurt** | 23% | 73% |
| **Cheese** | 8% | 45% |
| **Vegetable oil** | 38% | 55% |
| **Cereal** | 92% | 100% |
| **Rice** | 31% | 55% |
| **Bread** | 8% | 73% |
| **Pasta** | 8% | 27% |
| **Biscuits** | 15% | 100% |
| **Fruit** | 85% | 91% |
| **Fruit juice** | 31% | 55% |
| **Vegetables** | 85% | 100% |
| **Legumes** | 15% | 73% |
| **Potatoes** | 62% | 82% |
| **Egg** | 8% | 82% |
| **Meat** | 15% | 82% |
| **Chicken** | 62% | 100% |
| **Fish** | 8% | 91% |

**Table S1.** Information on consumption of different foods, obtained from questionnaires answered by the infants’ parents.
