# Supplementary material for: Microbial Succession in the Gut: Directional Trends of Taxonomic and Functional Change in a Birth Cohort of Spanish Infants
Source: PLoS Genet. 2014 Jun 5;10(6):e1004406. doi: 10.1371/journal.pgen.1004406 (PMC4046925; doi:10.1371/journal.pgen.1004406)
Supplement: Table S2 — Details of pyrosequencing reads and annotation per individual sample. (DOCX) [file pgen.1004406.s008.docx]

**Table S2** Details of pyrosequencing reads and annotation per individual sample.

| **Sample** | **Unique Reads** | **Genes Found** | **LCA Taxonomic Annotation** | **Functional Annotation** | **Average Read Length (bp)** |
| --- | --- | --- | --- | --- | --- |
| **MIP01-MA** | 63733 | 131925 | 101825 | 7809 | 446.27 |
| **MIP01-MB** | 129617 | 236016 | 209895 | 13793 | 366.00 |
| **MIP01-I1** | 48997 | 97623 | 86339 | 10697 | 437.46 |
| **MIP01-I2** | 34112 | 68793 | 60799 | 7382 | 443.14 |
| **MIP01-I3** | 49682 | 88309 | 71847 | 5666 | 322.77 |
| **MIP01-I4** | 37929 | 68759 | 60984 | 5740 | 365.07 |
| **MIP01-I5** | 32133 | 56102 | 49236 | 2211 | 268.34 |
| **MIP02-MA** | 63106 | 126902 | 100760 | 7079 | 401.80 |
| **MIP02-MB** | 36969 | 64422 | 57950 | 4226 | 362.00 |
| **MIP02-I1** | 54134 | 103844 | 94393 | 6852 | 417.47 |
| **MIP02-I2** | 68139 | 127653 | 113665 | 8852 | 353.88 |
| **MIP02-I3** | 56541 | 106410 | 96719 | 6626 | 333.28 |
| **MIP02-I4** | 33202 | 57333 | 51459 | 1956 | 279.81 |
| **MIP02-I5** | 41627 | 83319 | 76018 | 3924 | 366.55 |
| **MIP03-MA** | 67047 | 139735 | 119563 | 6992 | 442.18 |
| **MIP03-MB** | 76388 | 152863 | 138868 | 6405 | 391.78 |
| **MIP03-I1** | 32328 | 65924 | 58888 | 4349 | 437.30 |
| **MIP03-I2** | 52535 | 99544 | 89982 | 4749 | 370.02 |
| **MIP03-I3** | 36075 | 72013 | 64569 | 5473 | 437.90 |
| **MIP03-I4** | 42326 | 84398 | 76481 | 4449 | 417.74 |
| **MIP03-I5** | 37469 | 60176 | 54945 | 3371 | 319.00 |
| **MIP06-MA** | 34111 | 73111 | 64168 | 4585 | 437.36 |
| **MIP06-MB** | 47640 | 94927 | 86741 | 4277 | 389.25 |
| **MIP06-I1** | 47759 | 89228 | 85382 | 8108 | 421.48 |
| **MIP06-I2** | 94642 | 177917 | 151961 | 9877 | 354.62 |
| **MIP06-I3** | 97160 | 181821 | 158989 | 10084 | 353.23 |
| **MIP06-I4** | 35807 | 59904 | 51316 | 3076 | 278.47 |
| **MIP06-I5** | 74506 | 146868 | 129785 | 8811 | 389.70 |
| **MIP07-MA** | 40319 | 69365 | 57888 | 3025 | 294.00 |
| **MIP07-MB** | 65320 | 134656 | 121253 | 6433 | 371.83 |
| **MIP07-I1** | 60200 | 94237 | 88174 | 5722 | 265.95 |
| **MIP07-I3** | 48583 | 95613 | 85361 | 6075 | 386.60 |
| **MIP07-I4** | 29459 | 61089 | 55047 | 3242 | 387.49 |
| **MIP07-I5** | 34201 | 71762 | 66134 | 3180 | 377.86 |
| **MIP08-MA** | 41754 | 73911 | 61909 | 2745 | 312.46 |
| **MIP08-MB** | 75031 | 131836 | 118646 | 8649 | 354.00 |
| **MIP08-I1** | 38916 | 62653 | 57890 | 4504 | 297.32 |
| **MIP08-I2** | 114327 | 205008 | 193055 | 9912 | 293.13 |
| **MIP08-I3** | 83221 | 144388 | 137021 | 7226 | 273.92 |
| **MIP08-I4** | 28537 | 57999 | 52108 | 2932 | 396.00 |
| **MIP08-I5** | 93884 | 196536 | 176186 | 9752 | 389.87 |
| **MIP09-MA** | 60669 | 100871 | 84598 | 3877 | 277.88 |
| **MIP09-MB** | 66391 | 132896 | 118376 | 7060 | 404.14 |
| **MIP09-I1** | 46528 | 79729 | 73106 | 3431 | 268.68 |
| **MIP09-I2** | 126587 | 217620 | 204820 | 8272 | 279.84 |
| **MIP09-I3** | 31783 | 63218 | 59017 | 4325 | 372.87 |
| **MIP09-I4** | 37809 | 70452 | 63221 | 4821 | 366.93 |
| **MIP09-I5** | 120955 | 238510 | 215785 | 14577 | 392.42 |
| **MIP12-MA** | 39942 | 66384 | 56882 | 2183 | 263.05 |
| **MIP12-MB** | 53639 | 89972 | 82332 | 4800 | 337.00 |
| **MIP12-I1** | 54887 | 92667 | 85917 | 4864 | 285.67 |
| **MIP12-I2** | 22579 | 40509 | 40509 | 5432 | 473.21 |
| **MIP12-I3** | 96917 | 162886 | 148430 | 10109 | 269.72 |
| **MIP12-I4** | 73669 | 137758 | 127494 | 9298 | 345.00 |
| **MIP12-I5** | 56937 | 94242 | 86560 | 4757 | 353.00 |
| **MIP13-MA** | 70102 | 143379 | 143248 | 7937 | 388.18 |
| **MIP13-MB** | 61745 | 102231 | 92679 | 5655 | 337.00 |
| **MIP13-I1** | 102227 | 176230 | 167010 | 7721 | 268.38 |
| **MIP13-I3** | 63444 | 107888 | 94242 | 3345 | 275.84 |
| **MIP13-I4** | 98643 | 164024 | 154475 | 4654 | 266.40 |
| **MIP13-I5** | 38394 | 78507 | 73064 | 3631 | 375.33 |
| **MIP16-MA** | 33329 | 64377 | 53184 | 2925 | 356.23 |
| **MIP16-MB** | 57764 | 105315 | 93722 | 6322 | 379.00 |
| **MIP16-I1** | 82551 | 138189 | 126865 | 5170 | 265.35 |
| **MIP16-I2** | 35252 | 59308 | 55964 | 2121 | 266.73 |
| **MIP16-I3** | 70961 | 136620 | 119139 | 10358 | 365.32 |
| **MIP16-I4** | 67331 | 116413 | 108094 | 7034 | 296.80 |
| **MIP16-I5** | 33520 | 58093 | 51386 | 3773 | 357.00 |
| **MIP17-MA** | 210618 | 362405 | 322300 | 11952 | 280.82 |
| **MIP17-MB** | 71636 | 123963 | 111783 | 9956 | 420.00 |
| **MIP17-I1** | 165617 | 290614 | 271713 | 15378 | 323.00 |
| **MIP17-I3** | 24185 | 52277 | 46813 | 2796 | 389.58 |
| **MIP17-I4** | 89276 | 158357 | 148190 | 11622 | 336.00 |
| **MIP17-I5** | 61334 | 130648 | 119894 | 4487 | 365.93 |
| **MIP19-MA** | 71131 | 123677 | 104642 | 4800 | 293.98 |
| **MIP19-MB** | 70910 | 120540 | 110065 | 6789 | 346.00 |
| **MIP19-I1** | 86245 | 151364 | 142931 | 7196 | 283.70 |
| **MIP19-I3** | 75041 | 125158 | 118067 | 4018 | 272.19 |
| **MIP19-I4** | 59830 | 104530 | 97570 | 4569 | 334.00 |
| **MIP19-I5** | 86758 | 155460 | 145141 | 7309 | 372.00 |
| **MIP21-MA** | 27108 | 47356 | 45521 | 3644 | 396.00 |
| **MIP21-MB** | 28473 | 45688 | 41010 | 2794 | 342.15 |
| **MIP21-I1** | 44706 | 76115 | 69945 | 6039 | 371.00 |
| **MIP21-I2** | 63979 | 106768 | 98125 | 7155 | 357.00 |
| **MIP21-I3** | 160907 | 261002 | 241569 | 12793 | 343.00 |
| **MIP21-I4** | 65065 | 94067 | 86209 | 4486 | 279.62 |
| **MIP21-I5** | 53944 | 85607 | 78253 | 4186 | 331.38 |
