# Supplementary material for: Microbial Succession in the Gut: Directional Trends of Taxonomic and Functional Change in a Birth Cohort of Spanish Infants
Source: PLoS Genet. 2014 Jun 5;10(6):e1004406. doi: 10.1371/journal.pgen.1004406 (PMC4046925; doi:10.1371/journal.pgen.1004406)
Supplement: Table S5 — Variability explained by constrained (CCA1) and unconstrained (CA1 and CA2) axes in Canonical Correspondence Analyses when the constraining variable is delivery type, use of peripartum antibiotic or exclusivity of breastfeeding. (DOCX) [file pgen.1004406.s011.docx]

**Table S5.** Variability explained by constrained (CCA1) and unconstrained (CA1 and CA2) axes in Canonical Correspondence Analyses when the constraining variable is delivery type, use of peripartum antibiotic or exclusivity of breastfeeding.

| **Constraining variable** | **Timepoint** | **Variability explained by CCA1 (%)** | **Variability explained by CA1 (%)** | **Variability explained by CA2 (%)** |
| --- | --- | --- | --- | --- |
| ***Delivery type*** |  |  |  |  |
|  | **I1** | 16 | 23 | 20 |
|  | **I2** | 22 | 28 | 20 |
|  | **I3** | 22 | 31 | 18 |
|  | **I4** | 10 | 33 | 23 |
|  | **I5** | 4 | 38 | 18 |
| ***Peripartum antibiotic*** |  |  |  |  |
|  | **I1** | 11 | 24 | 21 |
|  | **I2** | 19 | 27 | 23 |
|  | **I3** | 12 | 32 | 21 |
|  | **I4** | 4 | 34 | 27 |
|  | **I5** | 6 | 37 | 17 |
| ***Exclusive breastfeeding*** |  |  |  |  |
|  | **I1** | 7 | 26 | 22 |
|  | **I2** | 9 | 29 | 20 |
|  | **I3** | 5 | 32 | 26 |
|  | **I4** | 12 | 34 | 22 |
|  | **I5** | 11 | 38 | 17 |
