# Supplementary material for: Microbial Succession in the Gut: Directional Trends of Taxonomic and Functional Change in a Birth Cohort of Spanish Infants
Source: PLoS Genet. 2014 Jun 5;10(6):e1004406. doi: 10.1371/journal.pgen.1004406 (PMC4046925; doi:10.1371/journal.pgen.1004406)
Supplement: Table S6 — Richness (N and Chao1 estimator) and diversity (Shannon index) for taxonomic and functional data in individual samples. (DOCX) [file pgen.1004406.s012.docx]

**Table S6** Richness (N and Chao1 estimator) and diversity (Shannon index) for taxonomic and functional data in individual samples.

|  | **Taxonomical Data** | | | **Functional Data** | | |
| --- | --- | --- | --- | --- | --- | --- |
| **Sample** | **N** | **Chao1** | **Shannon** | **N** | **Chao1** | **Shannon** |
| **MIP01-I1** | 55.25 | 68.75 | 1.39 | 95.02 | 96.62 | 5.94 |
| **MIP01-I2** | 61.83 | 78.42 | 1.99 | 93.97 | 95.99 | 5.93 |
| **MIP01-I3** | 185.85 | 295.08 | 2.28 | 93.38 | 95.16 | 5.91 |
| **MIP01-I4** | 157.97 | 205.55 | 3.24 | 95.24 | 98.49 | 5.91 |
| **MIP01-I5** | 215.99 | 300.78 | 2.82 | 91.93 | 96.63 | 5.67 |
| **MIP01-MA** | 253.02 | 340.11 | 2.92 | 91.17 | 96.57 | 5.78 |
| **MIP01-MB** | 304.45 | 420.43 | 3.81 | 91.05 | 96.27 | 5.77 |
| **MIP02-I1** | 112.02 | 136.18 | 2.19 | 94.25 | 98.48 | 5.91 |
| **MIP02-I2** | 89.77 | 118.67 | 2.18 | 93.29 | 95.69 | 5.91 |
| **MIP02-I3** | 76.58 | 110.94 | 1.04 | 90.64 | 93.05 | 5.79 |
| **MIP02-I4** | 209.48 | 300.75 | 2.71 | 90.96 | 93.17 | 5.75 |
| **MIP02-I5** | 150.98 | 232.16 | 2.23 | 91.38 | 96.78 | 5.81 |
| **MIP02-MA** | 255.75 | 338.16 | 3.33 | 90.17 | 94.76 | 5.69 |
| **MIP02-MB** | 231.25 | 318.38 | 4.13 | 90.91 | 95.62 | 5.73 |
| **MIP03-I1** | 78.00 | 104.48 | 1.26 | 93.84 | 97.89 | 5.95 |
| **MIP03-I2** | 104.52 | 146.33 | 1.45 | 91.62 | 97.33 | 5.88 |
| **MIP03-I3** | 66.62 | 85.70 | 1.16 | 93.39 | 97.95 | 5.92 |
| **MIP03-I4** | 100.42 | 144.92 | 1.79 | 92.73 | 97.94 | 5.89 |
| **MIP03-I5** | 117.55 | 171.99 | 3.08 | 92.34 | 94.20 | 5.84 |
| **MIP03-MA** | 168.06 | 235.56 | 1.59 | 92.05 | 98.28 | 5.83 |
| **MIP03-MB** | 220.90 | 333.59 | 2.02 | 93.09 | 99.64 | 5.84 |
| **MIP06-I1** | 70.36 | 110.51 | 0.43 | 84.47 | 87.16 | 5.73 |
| **MIP06-I2** | 182.46 | 265.88 | 2.68 | 90.49 | 95.50 | 5.81 |
| **MIP06-I3** | 159.00 | 247.24 | 2.30 | 89.40 | 95.06 | 5.79 |
| **MIP06-I4** | 169.75 | 269.37 | 2.63 | 89.67 | 96.10 | 5.78 |
| **MIP06-I5** | 256.22 | 364.16 | 3.33 | 91.23 | 96.29 | 5.80 |
| **MIP06-MA** | 336.80 | 400.24 | 4.48 | 93.60 | 100.73 | 5.74 |
| **MIP06-MB** | 219.84 | 322.21 | 2.29 | 93.82 | 101.06 | 5.81 |
| **MIP07-I1** | 99.94 | 153.31 | 1.46 | 85.24 | 91.48 | 5.64 |
| **MIP07-I3** | 269.54 | 398.11 | 2.68 | 89.69 | 93.81 | 5.76 |
| **MIP07-I4** | 223.87 | 291.39 | 2.82 | 90.15 | 94.11 | 5.87 |
| **MIP07-I5** | 182.05 | 283.34 | 2.25 | 92.39 | 98.97 | 5.82 |
| **MIP07-MA** | 345.39 | 442.88 | 4.06 | 89.58 | 93.68 | 5.71 |
| **MIP07-MB** | 275.40 | 393.18 | 3.34 | 90.69 | 94.51 | 5.77 |
| **MIP08-I1** | 133.61 | 190.65 | 1.81 | 91.35 | 95.27 | 5.77 |
| **MIP08-I2** | 174.39 | 301.80 | 1.45 | 90.69 | 97.03 | 5.74 |
| **MIP08-I3** | 172.62 | 295.39 | 1.81 | 90.35 | 94.32 | 5.74 |
| **MIP08-I4** | 159.95 | 238.05 | 2.38 | 92.83 | 96.09 | 5.84 |
| **MIP08-I5** | 185.09 | 303.90 | 2.15 | 89.85 | 94.93 | 5.80 |
| **MIP08-MA** | 241.48 | 340.23 | 2.32 | 90.23 | 93.77 | 5.76 |
| **MIP08-MB** | 193.93 | 298.26 | 3.30 | 88.48 | 93.28 | 5.73 |
| **MIP09-I1** | 96.57 | 172.68 | 0.61 | 78.90 | 82.80 | 5.56 |
| **MIP09-I2** | 131.71 | 252.24 | 1.67 | 88.62 | 94.05 | 5.76 |
| **MIP09-I3** | 137.28 | 200.67 | 2.62 | 96.30 | 100.27 | 5.94 |
| **MIP09-I4** | 115.30 | 153.53 | 2.16 | 91.27 | 98.68 | 5.77 |
| **MIP09-I5** | 178.02 | 275.31 | 2.96 | 90.08 | 94.18 | 5.81 |
| **MIP09-MA** | 276.10 | 392.94 | 3.13 | 89.72 | 94.19 | 5.74 |
| **MIP09-MB** | 318.65 | 430.36 | 3.31 | 91.50 | 96.76 | 5.80 |
| **MIP12-I1** | 240.53 | 355.59 | 3.31 | 91.54 | 95.53 | 5.85 |
| **MIP12-I2** | 138.47 | 156.72 | 2.01 | 94.70 | 98.10 | 5.90 |
| **MIP12-I3** | 181.09 | 268.24 | 2.53 | 94.40 | 99.19 | 5.90 |
| **MIP12-I4** | 231.73 | 322.02 | 2.92 | 93.63 | 97.49 | 5.86 |
| **MIP12-I5** | 160.00 | 232.45 | 2.53 | 93.12 | 98.20 | 5.84 |
| **MIP12-MA** | 235.17 | 336.20 | 3.22 | 88.30 | 91.39 | 5.67 |
| **MIP12-MB** | 232.67 | 329.23 | 2.48 | 90.01 | 93.98 | 5.79 |
| **MIP13-I1** | 103.67 | 203.88 | 1.36 | 95.66 | 100.04 | 5.90 |
| **MIP13-I3** | 427.39 | 520.69 | 2.81 | 92.80 | 95.43 | 5.87 |
| **MIP13-I4** | 124.96 | 216.92 | 0.87 | 90.57 | 95.04 | 5.79 |
| **MIP13-I5** | 131.68 | 210.23 | 1.80 | 94.76 | 103.07 | 5.91 |
| **MIP13-MA** | 404.34 | 474.51 | 3.70 | 90.42 | 94.60 | 5.78 |
| **MIP13-MB** | 207.42 | 301.91 | 2.65 | 92.52 | 97.37 | 5.80 |
| **MIP16-I1** | 113.83 | 175.58 | 2.26 | 94.12 | 98.79 | 5.84 |
| **MIP16-I2** | 186.06 | 303.11 | 2.31 | 91.31 | 96.73 | 5.81 |
| **MIP16-I3** | 121.56 | 172.25 | 2.96 | 94.46 | 97.78 | 5.92 |
| **MIP16-I4** | 257.85 | 366.98 | 3.07 | 91.91 | 96.69 | 5.82 |
| **MIP16-I5** | 218.32 | 260.62 | 3.92 | 94.41 | 99.45 | 5.81 |
| **MIP16-MA** | 210.23 | 277.55 | 3.20 | 91.45 | 95.79 | 5.75 |
| **MIP16-MB** | 257.85 | 364.65 | 3.34 | 92.41 | 98.64 | 5.82 |
| **MIP17-I1** | 202.43 | 334.84 | 3.15 | 94.70 | 99.42 | 5.89 |
| **MIP17-I3** | 320.09 | 489.22 | 1.62 | 92.17 | 97.44 | 5.75 |
| **MIP17-I4** | 121.60 | 183.83 | 2.15 | 91.08 | 96.23 | 5.78 |
| **MIP17-I5** | 154.09 | 235.97 | 1.30 | 90.68 | 95.73 | 5.83 |
| **MIP17-MA** | 236.25 | 381.53 | 2.58 | 89.10 | 93.05 | 5.76 |
| **MIP17-MB** | 241.38 | 330.03 | 3.28 | 90.79 | 94.18 | 5.84 |
| **MIP19-I1** | 183.89 | 310.42 | 2.08 | 93.27 | 98.69 | 5.83 |
| **MIP19-I3** | 154.09 | 264.28 | 1.24 | 90.45 | 95.21 | 5.80 |
| **MIP19-I4** | 122.60 | 186.55 | 1.53 | 88.92 | 93.91 | 5.81 |
| **MIP19-I5** | 117.91 | 180.42 | 0.98 | 90.89 | 98.32 | 5.83 |
| **MIP19-MA** | 322.37 | 435.16 | 3.45 | 89.38 | 91.36 | 5.68 |
| **MIP19-MB** | 210.80 | 311.56 | 2.38 | 92.33 | 96.43 | 5.81 |
| **MIP21-I1** | 183.76 | 255.60 | 2.53 | 93.47 | 97.32 | 5.85 |
| **MIP21-I2** | 175.34 | 240.54 | 2.64 | 93.08 | 97.32 | 5.82 |
| **MIP21-I3** | 154.90 | 249.06 | 1.84 | 92.75 | 97.70 | 5.87 |
| **MIP21-I4** | 152.28 | 223.53 | 2.90 | 89.24 | 93.54 | 5.75 |
| **MIP21-I5** | 125.79 | 182.88 | 2.26 | 90.38 | 93.60 | 5.81 |
| **MIP21-MA** | 228.03 | 249.36 | 3.98 | 92.26 | 96.67 | 5.76 |
| **MIP21-MB** | 172.99 | 195.29 | 3.27 | 92.30 | 97.58 | 5.71 |
