# Supplementary material for: Microbial Succession in the Gut: Directional Trends of Taxonomic and Functional Change in a Birth Cohort of Spanish Infants
Source: PLoS Genet. 2014 Jun 5;10(6):e1004406. doi: 10.1371/journal.pgen.1004406 (PMC4046925; doi:10.1371/journal.pgen.1004406)
Supplement: Table S7 — p-values of ANOSIM pairwise comparisons between timepoints. Statistically significant values (p<0.05) are shown in red. (DOCX) [file pgen.1004406.s013.docx]

**Table S7** p-values of ANOSIM pairwise comparisons of microbiota composition between timepoints. Statistically significant values (p < 0.05) are shown in red.

| **Taxonomic composition comparisons** | | | | | | |
| --- | --- | --- | --- | --- | --- | --- |
|  | MB | I1 | I2 | I3 | I4 | I5 |
| MA | 0.2241 | 1e-04 | 0.0001 | 0.0001 | 0.0001 | 0.0003 |
| MB | NA | 1e-04 | 0.0001 | 0.0001 | 0.0001 | 0.0092 |
| I1 | NA | NA | 0.6392 | 0.4657 | 0.0893 | 0.0019 |
| I2 | NA | NA | NA | 0.9049 | 0.2144 | 0.0015 |
| I3 | NA | NA | NA | NA | 0.3113 | 0.0015 |
| I4 | NA | NA | NA | NA | NA | 0.0184 |

| **Functional composition comparisons** | | | | | | |
| --- | --- | --- | --- | --- | --- | --- |
|  | MB | I1 | I2 | I3 | I4 | I5 |
| MA | 0.0798 | 1e-04 | 0.0001 | 0.0001 | 0.0001 | 0.0002 |
| MB | NA | 1e-04 | 0.0001 | 0.0001 | 0.0001 | 0.0036 |
| I1 | NA | NA | 0.6911 | 0.4381 | 0.0135 | 0.0001 |
| I2 | NA | NA | NA | 0.9544 | 0.2044 | 0.0006 |
| I3 | NA | NA | NA | NA | 0.1989 | 0.0005 |
| I4 | NA | NA | NA | NA | NA | 0.0899 |
