# Supplementary material for: Microbial Succession in the Gut: Directional Trends of Taxonomic and Functional Change in a Birth Cohort of Spanish Infants
Source: PLoS Genet. 2014 Jun 5;10(6):e1004406. doi: 10.1371/journal.pgen.1004406 (PMC4046925; doi:10.1371/journal.pgen.1004406)
Supplement: Table S8 — Taxonomic and functional dissimilarities between timepoints estimated as the non-overlapping areas of the convex hulls representing them in the PCoAs of Figure 4C–D. Dissimilarity values above 0.80 are shown in red. (DOCX) [file pgen.1004406.s014.docx]

**Table S8** Taxonomic and functional dissimilarities between timepoints estimated as the non-overlapping areas of the convex hulls representing them in the PCoAs of Figure 6C-D. Dissimilarity values above 0.80 are shown in red.

| **Taxonomic dissimilarities** | | | | | | |
| --- | --- | --- | --- | --- | --- | --- |
|  | I2 | I3 | I4 | I5 | MA | MB |
| I1 | 0.6498659 | 0.6144533 | 0.7273806 | 0.8635998 | 0.9811679 | 0.9710544 |
| I2 | NA | 0.5097943 | 0.7129247 | 0.8689479 | 0.9803610 | 0.9685655 |
| I3 | NA | NA | 0.5253540 | 0.7743676 | 0.9659283 | 0.9486293 |
| I4 | NA | NA | NA | 0.6853472 | 0.9593822 | 0.9469883 |
| I5 | NA | NA | NA | NA | 0.8619352 | 0.7877890 |
| MA | NA | NA | NA | NA | NA | 0.4500280 |

| **Functional dissimilarities** | | | | | | |
| --- | --- | --- | --- | --- | --- | --- |
|  | I2 | I3 | I4 | I5 | MA | MB |
| I1 | 0.4838081 | 0.4739469 | 0.6852406 | 0.9868082 | 1.0000000 | 1.0000000 |
| I2 | NA | 0.4195351 | 0.5605117 | 0.9058996 | 1.0000000 | 1.0000000 |
| I3 | NA | NA | 0.5650762 | 0.8208541 | 0.9940537 | 0.9994443 |
| I4 | NA | NA | NA | 0.6805861 | 0.8990785 | 0.9126285 |
| I5 | NA | NA | NA | NA | 0.9809216 | 0.9521181 |
| MA | NA | NA | NA | NA | NA | 0.4792706 |
